# Global long non-coding RNA expression in the rostral anterior cingulate cortex of depressed suicides

**DOI:** 10.1038/s41398-018-0267-7

**Published:** 2018-10-18

**Authors:** Yi Zhou, Pierre-Eric Lutz, Yu Chang Wang, Jiannis Ragoussis, Gustavo Turecki

**Affiliations:** 1 McGill Group for Suicide Studies, McGill University, 6875 LaSalle Boulevard, Montreal, QC H4H 1R3 Canada; 2 Centre National de la Recherche Scientifique, Université de Strasbourg, Institut des Neurosciences Cellulaires et Intégratives, Strasbourg, France; 3 Fédération de Médecine Translationnelle de Strasbourg, Strasbourg, France; 4 McGill University and Génome Québec Innovation Centre, 740 Dr. Penfield Avenue, Room 7104, Montréal, QC H3A 0G1 Canada

## Abstract

Long non-coding RNAs (lncRNAs) are an emerging class of regulatory RNA that may be implicated in psychiatric disorders. Here we performed RNA-sequencing in the rostral anterior cingulate cortex of 26 depressed suicides and 24 matched controls. We first performed differential lncRNA expression analysis, and then conducted Weighted Gene Co-expression Network Analysis (WGCNA) to identify co-expression modules associating with depression and suicide. We identified 23 differentially expressed lncRNAs (FDR < 0.1) as well as their differentially expressed overlapping and antisense protein-coding genes. Several of these overlapping or antisense genes were associated with interferon signaling, which is a component of the innate immune response. Using WGCNA, we identified modules of highly co-expressed genes associated with depression and suicide and found protein-coding genes highly connected to differentially expressed lncRNAs within these modules. These protein-coding genes were located distal to their associated lncRNAs and were found to be part of several GO terms enriched in the significant modules, which include: cytoskeleton organization, plasma membrane, cell adhesion, nucleus, DNA-binding, and regulation of dendrite development and morphology. Altogether, we report that lncRNAs are differentially expressed in the brains of depressed individuals who died by suicide and may represent regulators of important molecular functions and biological processes.

## Introduction

Depression is a leading cause of disability affecting 300 million people worldwide, according to estimates by World Health Organization^1^, and the single most important risk factor for suicide^2^. Various biological systems are believed to underlie depression^3^ and in order to gain insight into its possible underlying pathways, studies have investigated gene expression patterns that are altered in the depressed brain. More recently, there has been growing interest in the investigation of the epigenetic factors explaining gene dysregulation in this disorder^4^. Among these, non-coding RNAs have been identified as important regulators of gene expression. Importantly, an emerging class of non-coding RNAs, called long non-coding RNAs (lncRNAs), have recently become identified as important players involved in the mechanisms underlying disease, including mental health disorders^5^.

LncRNAs are defined as RNA molecules greater than 200 bp in length with low protein-coding potential. They are found throughout the genome and are generally categorized based on their relation to other known genes. For example, lncRNAs can be antisense, sense overlapping, intronic, and intergenic^6^. Early in their investigation, it was unclear whether lncRNAs were functional or products of transcriptional noise. Recent investigations revealed that lncRNAs exhibit sequence conservation near their promoters, and high secondary structural conservation^7^. In addition, they show high tissue-specific^8^ and activity-dependent^9,10^ expression, therefore supporting the hypothesis that they fulfill biological functions.

LncRNAs are enriched in nuclear fractions^11^, suggesting that they interact with chromatin and DNA. Indeed, the expression of protein-coding genes has been shown to be regulated by overlapping or antisense lncRNAs^12^. For example, the antisense lncRNA *KCNQ1OT1* interacts with histone methyl-transferases to form a repressive domain around the *KCNQ1* gene, thereby regulating its expression^13^. While such local *cis* regulatory mechanisms represent an important mode of lncRNA function, their interaction with protein complexes regulating the expression of more distal genes in *trans*, has also been described. For example, the lncRNA *HOTAIR* found in the HOXC locus on chromosome 12, interacts with PRC2 to regulate the expression of HOXD locus genes on chromosome 2^14,15^. As well, the process of lncRNA transcription per se may serve to mediate certain regulatory processes, such as the recruitment of transcription factors and other regulatory proteins, affecting the expression of target genes^16^. Finally, owing to their ability to fold into modular secondary structures, lncRNAs have also been reported to act as molecular sinks for particular proteins and to act as scaffolds upon which various protein complexes can be assembled^17^.

While there have already been some reports of associations between lncRNAs and psychiatric phenotypes^18,19^, little is known about their role in depression and suicide. The neuropathology of suicide broadly implicates a dysregulated stress response, immune, and neurotransmitter/neuromodulator systems that exhibit altered gene expression in the brains of depressed individuals who died by suicide^20^. Our group and others have previously reported changes in the expression of polyamine-related genes in suicide brains^21^, some of which are regulated by miRNAs, another class of non-coding RNAs^22,23^. Given that lncRNAs have also been shown to fulfill important regulatory functions, we aim to explore whether they may also be important in contributing to gene dysregulation in the brains of depressed suicides. While a handful of studies have reported changes in lncRNA expression in the peripheral blood of depressed patients^24^, none have broadly characterized lncRNAs in the brains of depressed individuals who died by suicide, to our knowledge. Thus, there is a great need for the characterization of lncRNA expression and for the identification of dysregulated genes targeted by lncRNAs in the brain and in the context of depression and suicide.

Here we report a transcriptome-wide lncRNA expression profiling in the rostral anterior cingulate cortex (rACC) of depressed suicides and controls, using RNA-sequencing (RNA-seq). We focused on the ACC as it is a critical structure for the assessment of emotional salience and the processing of motivational information^25^. ACC dysfunction is implicated in depression as altered gray matter volume as well as increased metabolic activity have been found in depressed subjects which are ameliorated with antidepressant treatment^26^. Furthermore, several studies have previously identified differentially expressed genes in the ACC of suicides, including stress^27^ and myelin^28^ related genes. In the present study, we identified and validated several differentially expressed lncRNAs, as well as their potential proximally regulated *cis* target genes by identifying antisense or overlapping dysregulated protein-coding genes. Furthermore, we combined Weighted Gene Co-expression Network Analysis (WGCNA)^29^ with Gene Ontology (GO) in order to identify potential lncRNA targets located distal to the lncRNA gene and to determine their biological and molecular significance in the brains of depressed individuals who died by suicide.

## Materials and methods

### Cohort and overlap with previous studies

Ethical approval was obtained from The Institutional Review Board of the Douglas Mental Health Institute, and written informed consent was obtained from the family. Individuals who died by suicide during a major depressive episode (*N* = 26), as well as control subjects who died naturally or by accident, with no history of psychopathology (*N* = 24), were included in this study. The sample size was chosen based on previous studies conducted by our group to investigate molecular changes in the brain of depressed individuals^30,31^. Psychological autopsies were performed by trained clinicians on both controls and cases, as described previously and validated by our group and others^32,33^. Diagnoses were assigned based on DSM-IV criteria by panels blind to group status. There were no significant differences in age, post mortem interval (PMI), brain pH, and RNA-integrity number (RIN) values between the two groups (Supplementary Table 1). Both male and female subjects were used in this study and there were no significant differences in the proportion of males and females in each group (Supplementary Table 1). A few depressed subjects had substance use disorders, a factor that was explored and controlled for in downstream analyses. Of note, the RNA-sequencing data that was generated using this cohort was part of a previous study (see^28^) that focused on coding RNA transcripts. We have included the details of this analysis in the supplementary materials and methods. However, here we have conducted and report separate studies specifically devoted to the investigation of lncRNAs.

### Tissue dissection

Brain tissue was obtained from the Douglas-Bell Canada Brain Bank (douglasbrainbank.ca/). 200 mg of tissue was dissected from the rACC on 0.5 cm-thick coronal sections, as described in supplementary material (Supplementary Figure 1).

### RNA-sequencing

RNA was extracted from rACC tissue using the RNeasy Lipid Tissue Mini Kit (Qiagen, Valencia, CA, USA). RNA quality (RIN values) was measured using an Agilent 2100 Bioanalyzer (Agilent Technologies, Germany), and only samples with RIN values greater than 5 were used. RNA-seq libraries were prepared by McGill University’s Innovation Centre, as described previously^34^, using the Ribo-Zero Gold rRNA Removal Kit (Illumina, MRZG12324, USA) and the TruSeq Stranded Total RNA Library Prep Kit (Illumina, RS-122-2201, USA). DNA libraries were pooled into groups of 3–4 per lane of an Illumina’s HiSeq 2000 (Illumina, USA), and loaded across 2 flow cells to generate 100 base pair (bp) paired-end reads.

### Data processing and RNA-seq differential gene expression analysis

As described previously^35^, quality control was first performed. Trimmed reads were aligned to the human hg19 reference genome, using TopHat2^36^. We used a lncRNA-specific gene annotation from GENCODE (Release 19 (GRCh37.p13) Long non-coding RNA gene annotation), which contains 13,000 annotated lncRNA genes, to count reads aligning with lncRNA genes using HTSeq-Count^37^. We first focused only on lncRNA gene expression to increase our ability and power to detect significantly differentially expressed lncRNAs using DESeq2 and to overcome normalization bias^38^. While this approach can lead to increased assignment of sequenced reads to lncRNA gene features, we followed up these results with qPCR experiments using primers carefully designed to target RNA sequences specific for regions unique to these lncRNAs. LncRNAs with an average of <20 reads per library were discarded from further analysis as they are more likely to be transcriptional noise. Fifteen genes were removed due to having sequenced reads accounting for >0.4% of the total number of reads, which could skew the data normalization. These were as follows: *BCYRN1*, *KCNQ1OT1*, *LINC00657*, *MALAT1*, *MEG3*, *MIAT*, *NEAT1*, *NEFL*, *OIP5-AS1*, *RMRP*, *RN7SL1*, *RPPH1*, *SNHG14*, *SNORD3A*, *SYN2*. A principal component analysis (PCA) was performed to identify and evaluate effects of covariates. Age, brain pH, PMI, RIN, sex, and substance use disorder (SUD) status were controlled for in the differential lncRNA expression analysis. The top lncRNAs passing an adjusted p-value (FDR < 0.1) were selected for further investigation. The expression of protein-coding genes was similarly characterized from our RNA-seq dataset using the Comprehensive gene annotation from GENCODE (Release 19, GRCh37.p13).

### cDNA synthesis

Invitrogen’s M-MLV reverse transcriptase (ThermoFischer, 28025013, USA) and random hexamer primers were used for cDNA synthesis. For the Biomark HD (Fluidigm, USA) high throughput qPCR validation, spike in RNAs (C1™ RNA Standard Assays, RNA spikes 1, 4, and 7, Fluidigm, USA) were incorporated into the sample RNAs to serve as controls during cDNA synthesis.

### Primer design

We designed and tested qPCR primers (Integrated DNA Technologies, USA) for differentially expressed lncRNA, potential lncRNA target genes, and housekeeping genes (HKG) (Supplementary Table 2). PCR products amplified from pooled sample cDNA were run on a PerkinElmer’s microfluidic LabChip to check the accuracy and specificity of each primer pair.

### BioMark HD high throughput RT-qPCR validation

#### Pre-amplification

LncRNA and HKG primers were pooled and 15 cycles of PCR pre-amplification, using SsoAdvanced PreAmp Supermix (Bio-Rad, 1725160, USA), were done for each sample. Samples were then diluted 5×.

#### High throughput qPCR

We used Fluidigm’s Biomark HD to validate lncRNA expression. Pre-amplified samples and primers were loaded onto a 96 by 96 integrated fluidic circuit (IFC) chip (Fluidigm, USA). Gene expression was quantified by the relative standard curve method^31^, and using the SsoFast EvaGreen Supermix (Bio-Rad, 1725210, USA). LncRNA expression was normalized against the geometric mean of 3 HKG’s: *ARHGEF12*, *B-ACTIN*, and *TUBA1A*. Each of these 3 housekeeping genes was stable across conditions in both the RNA-seq and qPCR data (Supplementary Table 3). To assess their stability in qPCR data, each housekeeping gene was normalized to the geometric mean of the other 2 housekeeping genes.

### qPCR

RT-qPCR was done to validate the differential expression of potential lncRNA cis-targets as described previously^31^.

### Weighted gene co-expression network analysis and identification of depression modules

We used WGCNA^29^ to identify co-expression modules using RNA-seq expression data from controls and depressed suicides combined. A 1-step network construction and module detection method was used along with the Dynamic Tree Cut method. A soft thresholding power of 5 was selected according to an analysis of network topology, the TOMType was set to the default parameter “unsigned”, and the branch mergeCutHeight was set at 0.4. Module detection was done in a single block with a maximum block size of 25,000 genes. We then searched for associations between modules and various sample traits. By default, the WGCNA package correlates a module eigengene with each external sample trait to identify significant associations. Such associations, however, do not take into account potential confounding contributions of other sample traits. To overcome this, we investigated associations between depression and module eigengenes while controlling for co-variates, as described previously:^39^ eigengenes were used in a general linear model (GLM) using depression as a fixed factor while controlling for sample traits (Sex, SUD, age, brain pH, PMI, and RIN) as covariates. The significance of module eigengene associations with depression were corrected by the Benjamini-Hochberg procedure (FDR < 0.1).

### TopGO gene ontology analysis

To determine the functional significance of modules associated with depression and suicide, we performed gene ontology enrichment analysis on genes within each depression and suicide associated module using the topGO package^40^. We chose to use the *elim* algorithm and Fisher test statistic to identify significantly enriched gene ontologies. The org.Hs.eg.db annotation package was used for gene mapping to GO terms and we specified a gene universe consisting of all the genes identified by our RNA-seq analysis.

### qPCR statistical analysis

Statistical analysis for qPCR data was done using the SPSS software. A general linear model was used to determine the significance of the Group factor (depressed suicides vs control) on gene expression while taking into account Sex, SUD, brain pH, PMI, age, and RIN as co-variates. Some qPCR lncRNA expression data sets were log transformed, to ensure the normality of the data and equal variances, in order to carry out parametric statistical testing. Grubb’s outlier test was used to identify sample outliers.

## Results

### Exploratory analysis

RNA-sequencing produced an average of 56 million reads per subject with 43 million reads aligning to the human genome. The expression data for 2670 lncRNAs were included in downstream analysis, after filtering, with an average of 432 000 reads aligning to lncRNA genes per library. Sex was a significant covariate that clearly differentiated samples in the PCA (Supplementary Figure 2A). This was due, as expected, to the 1000-fold higher expression of lncRNA *XIST* in females compared to males, which contributed significantly to sample variations in lncRNA expression. After removing reads aligning to the *XIST* gene, much of the variation due to sex was eliminated (Supplementary Figure 2B). However, sex was still a significant factor that could account for variations in gene expression and was included as a co-variate in subsequent analyses.

At the moment of death, 22% of the subjects had toxicological evidence of benzodiazepines and 14% had evidence of acetaminophen. Other medications were present in less than 10% of the total sample and were not evaluated (Supplementary Table 4A). Presence of neither medication was associated with the top 4 principal components (each accounting for greater than 5% of the total expression variation) indicating they were not likely to be significant factors affecting lncRNA expression (Supplementary Table 4B). Furthermore, including either medication as a covariate in our downstream qPCR analyses did not have a significant effect on lncRNA differential expression (Supplementary Table 5A and 5B), confirming these medications were not significant factors contributing to lncRNA expression. As there was significant overlap between samples exhibiting presence of both medications (4 out of 7 subjects with acetaminophen also had benzodiazepines), we evaluated the effects of each medication individually.

Hierarchical clustering was done to visualize between-sample variability, and their relation to sample traits (Supplementary Figure 2C). No significant outliers were identified in these analyses, and all libraries were included in differential expression analysis.

### Differential lncRNA expression analysis

**Fig. 1:**
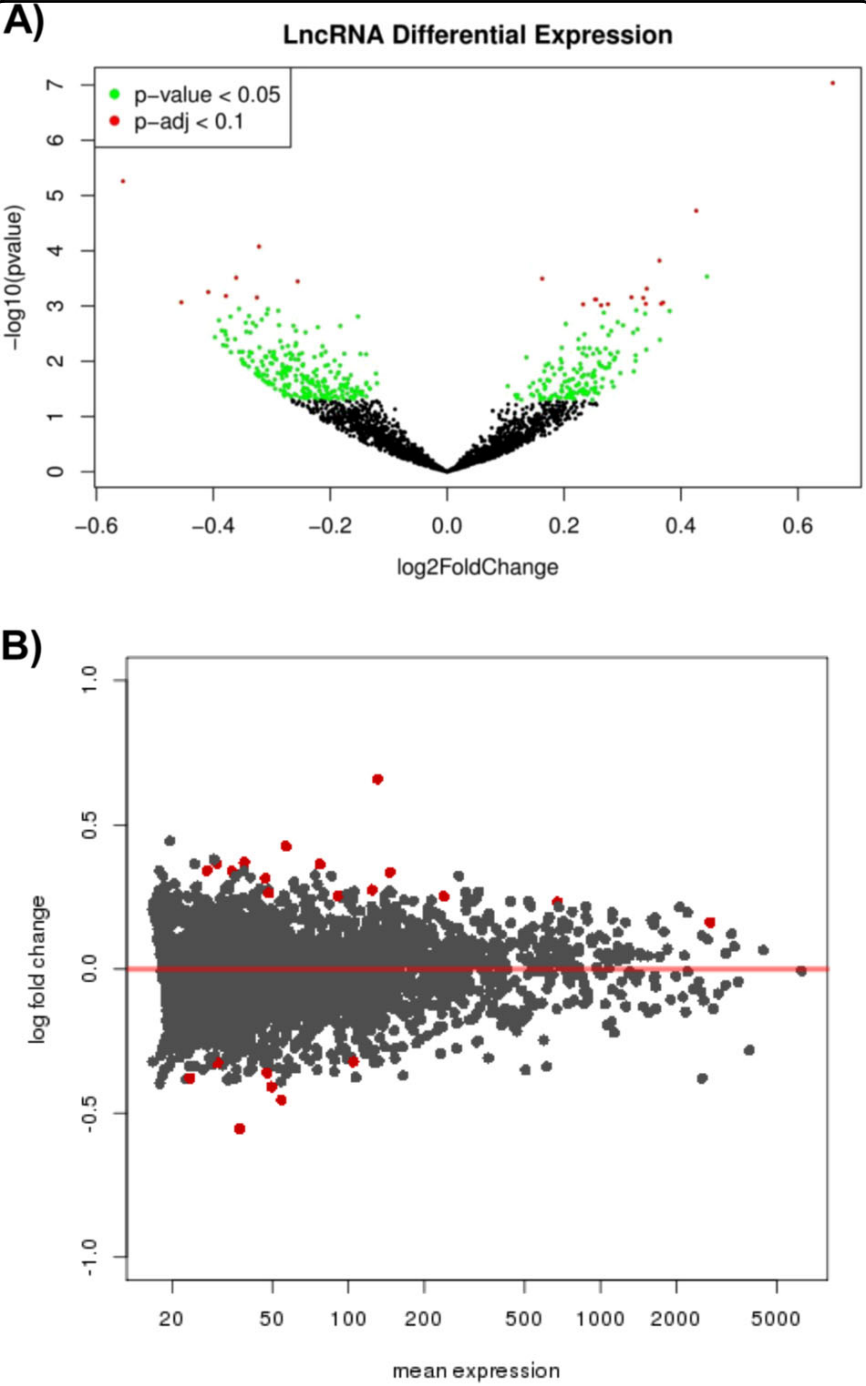
**Transcriptome wide lncRNA differential expression analysis**. **a** A volcano plot summarizing the differential expression of 2670 long non-coding RNAs (lncRNAs) with *p* values plotted against their fold changes. **b** An MA plot with LncRNA fold changes plotted against their mean expression levels (normalized read counts). The red points indicate lncRNAs with p-adj (FDR) < 0.1

Differential expression analysis was performed for the aforementioned 2670 lncRNAs. When considering nominal *p* values (*p* < 0.05), approximately 13% of lncRNAs (364/2670) were differentially expressed, with 60% of these being downregulated in depressed suicides (217/364) (Fig. 1a, and Supplementary Table 10).

23 differentially expressed lncRNAs passed genome-wide multiple testing corrections (Fig. 1b). Interestingly, of these, more were upregulated (15/23) than downregulated (Table 1). Of note, 2 of these 23 lncRNAs were identified as processed transcripts (*AC004019.18* and *DYX1C1-CCPG1)* that almost perfectly overlapped with protein-coding genes located on the same strand. As it would be extremely challenging to discriminate expression of these two lncRNAs from the expression of their corresponding protein-coding genes, they were excluded from further analyses.

**Table 1:**
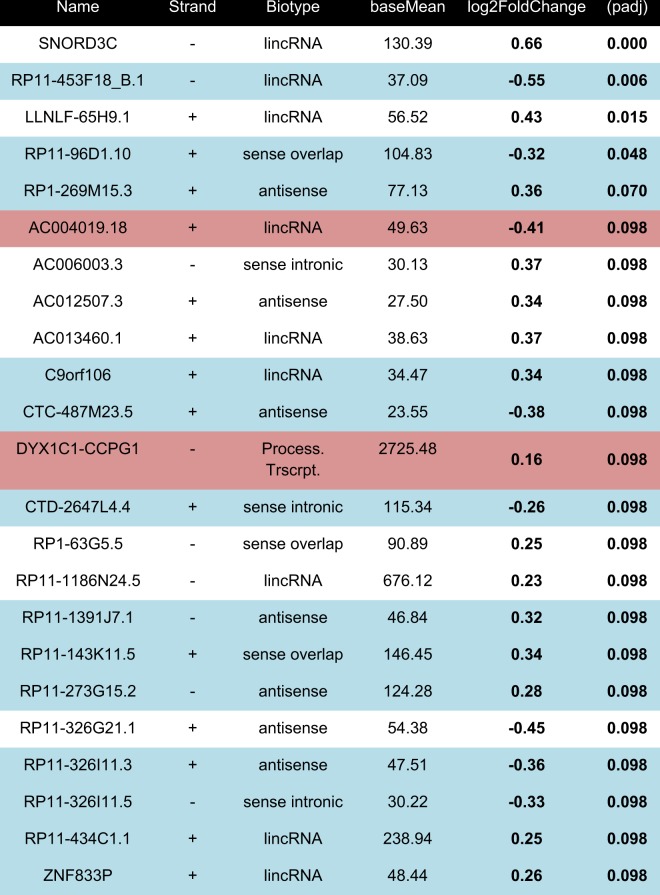
Top 23 differentially expressed long non-coding RNAs (p-adj < 0.1)

qPCR primers were successfully designed for lncRNAs highlighted in blue. LncRNAs highlighted in red were processed transcripts, which contained exons on the same strand and overlapping significantly with protein-coding genes and were omitted from further analyses

### Validating differentially expressed lncRNA with RT-qPCR

We then conducted qPCR experiments to validate the most significant findings, and designed primer pairs for 21 lncRNAs. Only 13 primer pairs, however, allowed for accurate and efficient quantification of the corresponding lncRNAs (Table 1). This was expected, as several factors contribute to the technical difficulty in designing primers to quantify lncRNA expression, such as low expression levels when compared to mRNA, overlapping organization with protein-coding genes, or poorly characterized splicing patterns^41^.

We found highly significant correlations between RNA-seq and RT-qPCR expression values for each lncRNA across the 50 samples (Supplementary Table 6), indicating high reliability across these experimental approaches. Furthermore, we validated significant differential expression for 9 out of the 13 lncRNAs that we could reliably quantify by qPCR (Supplementary Figure 3). Of these, 4 were antisense (*CTC-487M23.5, RP11-273G15.2, RP11-326I11.3*, and *RP1-269M15.3*), 1 was sense-overlapping (*RP11-96D1.10*), 1 was sense-intronic (*CTD-2647L4.4*), and 3 were intergenic (*RP11-453F18_B.1, RP11-434C1.1*, and *ZNF833P*).

### Identifying potential antisense or overlapping lncRNA cis-targets

**Fig. 2:**
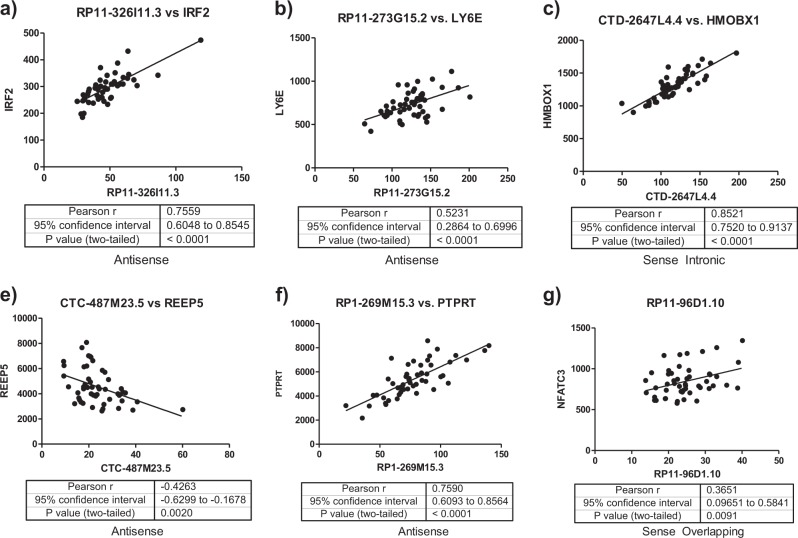
**LncRNAassociationswithpotentialcisgenetargets.** **a**–**g** Correlations (in terms of RNA-seq normalized read counts) between 6 differentially expressed long non-coding RNAs (lncRNAs) and antisense or overlapping protein-coding genes identified as potential cis-targets are shown

**Fig. 3:**
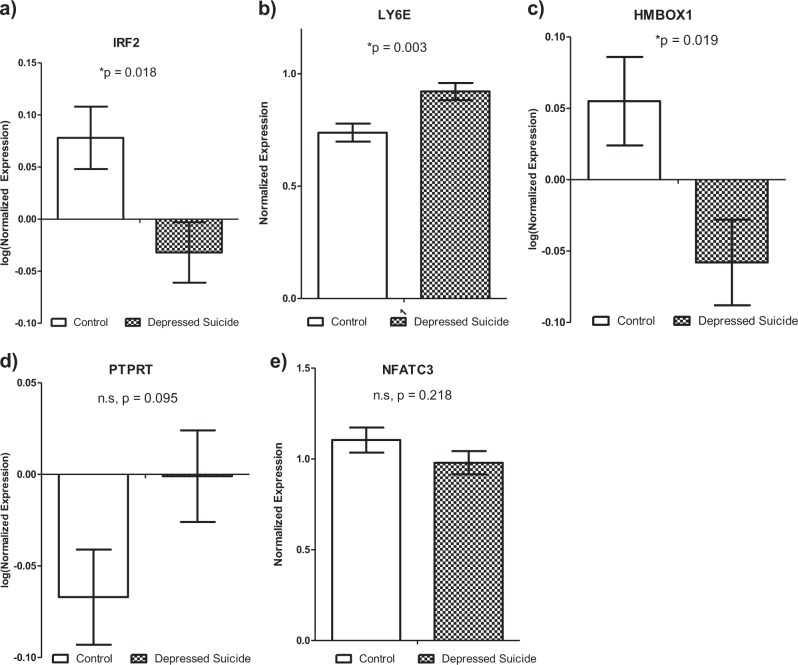
**Differentially expressed potential lncRNA** ***cis*** **gene targets.** **a**–**e** RT-qPCR validation of differentially expressed antisense and overlapping genes

Protein-coding genes antisense to or overlapping with differentially expressed lncRNAs were identified as potential lncRNA *cis* targets. Using full transcriptomic RNA-seq data available from the same samples included in this study, we found the expression of 6 lncRNAs were significantly correlated with one antisense or overlapping protein-coding gene each (Fig. 2a–g). The protein-coding genes were *IRF2, LY6E, HMBOX1, PTPRT, NFATC3*, and *REEP5*. All of these genes were nominally differentially expressed (*p* < 0.05) except *REEP5* (Supplementary Table 7). Differential expression of 3 out of 5 of these protein-coding genes (*IRF2, LY6E*, and *HMBOX1*) was further validated by qPCR (Fig. 3a–e). *IRF2* is antisense to lncRNA *RP11-326I11.3*, and encodes a transcription factor (TF) involved in interferon signaling^42^. *LY6E* is antisense to lncRNA *RP11-273G15.2* and is an interferon stimulated gene with immuno-modulatory functions^43^. Finally, lncRNA *CTD-2647L4.4* is sense intronic to *HMBOX1*, which encodes a TF involved in transcriptional repression, including of interferon genes^44^.

### Identifying other potential lncRNA *cis* and *trans* targets

LncRNAs have also been shown to regulate more distal gene targets through both *cis* and *trans* regulatory mechanisms^45^. To identify such potential regulatory targets, we took a 2-step approach: first, we performed a WGCNA analysis to identify modules of co-expressed genes; then, we searched within each module for significant correlations between expression of lncRNAs and distal protein-coding genes. The WGCNA network was constructed using both protein-coding and lncRNAs genes (Supplementary Figure 4) and 10 distinct modules of co-expressed genes were detected (Supplementary Table 8). Among these, the Brown and Blue modules showed significant associations with depression and suicide (Supplementary Table 8). Interestingly, 6 of the previously validated differentially expressed lncRNAs were members of the Blue module and 1 was a member of the Brown module (Supplementary Table 9). To determine the functional significance of these 2 modules, we performed Gene Ontology (GO) analysis and report in Supplementary Figures 5 and 6 the top 10 GO terms in each GO class for both the Brown and Blue modules, respectively.

Within the Brown and Blue modules, we then sought to determine associations between protein-coding genes and lncRNAs that were not antisense or overlapping with each other. To this end, we first identified the top 10 genes with the highest weighted correlation with each depression-associated lncRNA within the Brown and Blue modules. Of the top 10 most co-expressed genes with each lncRNA, those which showed nominal differential expression (*p* < 0.05) and also associated with a GO term significantly enriched within the corresponding co-expression module may represent potential biologically relevant lncRNA targets (Table 2). In this way, we identified 15 distinct protein-coding genes in the Blue module and 5 protein-coding genes in the Brown module that associated with differentially expressed lncRNAs and depression and suicide. In the Blue module, these protein-coding genes were predominantly associated with the plasma membrane, regulation of actin organization binding, and cell adhesion, while those in the Brown module were notably associated with the nucleus, DNA-binding and regulation of transcription, and regulation of dendrite development and morphogenesis.

**Table 2:** Differentially expressed lncRNA distal gene targets and associated GO terms in the Blue and Brown modules

LncRNA	Gene target	Weight	Associated GO	GO class	GO Description
CTC-487M23.5 (Blue module)	KANK1	0.228	GO:0007155	BP	Cell adhesion
			GO:0032956	BP	Regulation of actin cytoskeleton organiz…
			GO:0005886	CC	Plasma membrane
	DOCK1	0.212	GO:0043547	BP	Positive regulation of GTPase activity
	FNBP1	0.212	GO:0005886	CC	Plasma membrane
CTD-2647L4.4 (Blue module)	YWHAH	0.229	GO:0003779	MF	Actin binding
			GO:0005886	CC	Plasma membrane
RP11-453F18_B.1 (Blue module)	KANK1	0.204	GO:0007155	BP	Cell adhesion
			GO:0032956	BP	Regulation of actin cytoskeleton organiz…
			GO:0005886	CC	Plasma membrane
	TJP1	0.199	GO:0016324	CC	Apical plasma membrane
			GO:0005886	CC	Plasma membrane
	DOCK1	0.212	GO:0043547	BP	Positive regulation of GTPase activity
	FNBP1	0.211	GO:0005886	CC	Plasma membrane
RP11-273G15.2 (Blue module)	TCF12	0.165	GO:0071837	MF	HMG box domain binding
	GM2A	0.154	GO:0005887	CC	Integral component of plasma membrane
			GO:0005886	CC	Plasma membrane
			GO:0016021	CC	Integral component of membrane
	SNCB	0.155	GO:0005509	MF	Calcium ion binding
	YES1	0.156	GO:0007155	BP	Cell adhesion
			GO:0005886	CC	Plasma membrane
			GO:0015629	CC	Actin cytoskeleton
	VAMP3	0.152	GO:0007155	BP	Cell adhesion
			GO:0005886	CC	Plasma membrane
			GO:0016324	CC	Apical plasma membrane
			GO:0009986	CC	Cell surface
			GO:0016021	CC	Integral component of membrane
	RDX	0.154	GO:0003779	MF	Actin binding
			GO:0007155	BP	Cell adhesion
			GO:0032956	BP	Regulation of actin cytoskeleton organiz…
			GO:0030027	CC	Lamellipodium
			GO:0016324	CC	Apical plasma membrane
			GO:0005615	CC	Extracellular space
			GO:0015629	CC	Actin cytoskeleton
	RUSC1	0.165	GO:0003779	MF	Actin binding
			GO:0005886	CC	Plasma membrane
RP11-326I11.3 (Blue module)	TJP1	0.200	GO:0016324	CC	Apical plasma membrane
			GO:0005886	CC	Plasma membrane
	DOCK1	0.199	GO:0043547	BP	Positive regulation of GTPase activity
	FNBP1	0.220	GO:0005886	CC	Plasma membrane
	YWHAH	0.193	GO:0003779	MF	Actin binding
			GO:0005886	CC	Plasma membrane
RP1-269M15.3 (Blue module)	SGPL1	0.191	GO:0016021	CC	Integral component of membrane
	KANK1	0.222	GO:0007155	BP	Cell adhesion
			GO:0032956	BP	Regulation of actin cytoskeleton organiz…
			GO:0005886	CC	Plasma membrane
	DOCK1	0.198	GO:0043547	BP	Positive regulation of GTPase activity
	PPFIA1	0.195	GO:0007155	BP	Cell adhesion
			GO:0032956	BP	Regulation of actin cytoskeleton organiz…
	WASF2	0.189	GO:0003779	MF	Actin binding
			GO:0032956	BP	Regulation of actin cytoskeleton organiz…
			GO:0030027	CC	Lamellipodium
			GO:0015629	CC	Actin cytoskeleton
RP11-434C1.1 (Brown module)	CELSR3	0.130	GO:0046872	MF	Metal ion binding
	ATG3	0.122	GO:0016567	BP	Protein ubiquitination
	SRRM4	0.128	GO:0005634	CC	Nucleus
	DEK	0.122	GO:0003677	MF	DNA binding
			GO:0006355	BP	Regulation of transcription, DNA-templat…
			GO:0005634	CC	Nucleus
	GRIN1	0.124	GO:0046872	MF	Metal ion binding
			GO:0006355	BP	Regulation of transcription, DNA-templat…
			GO:0050773	BP	Regulation of dendrite development
			GO:0048813	BP	Dendrite morphogenesis

## Discussion

### Differential lncRNA expression in major depression and suicide

We found a significant number of lncRNAs were dysregulated, with a higher proportion showing decreased expression in the rACC of depressed suicides. Recent reports similarly characterized lncRNAs in the blood and peripheral blood mononuclear cells (PBMCs) of depressed patients. While one group reported a significantly higher proportion of downregulated lncRNAs in PBMCs^46^, another group reported a larger proportion of upregulated lncRNAs in whole blood from depressed subjects^24^. As lncRNAs are expressed in a highly tissue-specific manner, it was not surprising to see no overlap between the differentially expressed lncRNAs we identified in the brain and the top differentially expressed lncRNAs identified in peripheral samples reported by these previous studies. Whether lncRNA expression in the CNS relates to their expression in the periphery in the context of depression and suicide remains to be investigated.

Of note, we identified *RP1-269M15.3* as one of the top differentially expressed lncRNAs whose expression was previously reported to be stratified by 30 MDD related SNPs in the nucleus accumbens^47^. Therefore, *RP1-268M15.3* expression and associated genetic variation could contribute to depressive phenotypes, and should be investigated further.

### LncRNA associations with antisense and overlapping genes

We found that the expression of 3 differentially expressed lncRNAs were positively correlated with their corresponding antisense or overlapping genes, which was consistent with previous investigations characterizing lncRNA expression across tissues^8^. While positively correlated nearby genes could be indicative of an overall chromatin state around these genes, these associations could also reflect, in part, enhancer-like functions of lncRNAs. For example, there are the following reports in which: (i) siRNA-mediated depletion of lncRNAs leads to decreased expression of neighboring genes;^48^ (ii) lncRNAs transcribed from enhancer regions promote expression of nearby genes by acting as a decoy for the negative elongation factor complex in neurons^49^, and (iii) enhancer lncRNAs can mediate chromosomal looping, thereby bridging enhancers to their target genes^50^. Therefore, our results identify 3 lncRNAs as candidates for future functional studies designed to characterize their regulatory roles over nearby antisense and overlapping genes.

Most importantly, the 3 differentially expressed genes (*LY6E*, *IRF2*, and *HMBOX1*) identified as antisense to and overlapping with differentially expressed lncRNAs all have functions related to interferon (IFN) signaling. Among these, *LY6E* was recently reported to be upregulated in leukocytes from depressed patients after anti-depressant treatment^51^. The IFN signaling system has an important role in innate immune signaling as well as in CNS homeostasis and in psychiatric disorders, including major depression^52^. Therefore, our findings support the growing literature implicating the immune system in depression, and highlight the potential role of lncRNAs as mediators of IFN dysfunction in this disorder as well as in suicide.

### LncRNA potential associations with distal target genes

LncRNA genes have been shown to produce isoforms with distinct functions carried out through *cis* or *trans* regulatory mechanisms^53^. As such, a single lncRNA gene could regulate several target genes which may be distally located. Using WGCNA, we found that the Blue and Brown modules significantly associated with depression. GO analysis revealed an enrichment of protein-coding genes involved in cytoskeleton remodeling and in interactions between the cytoskeleton and the cell membrane (Table 2) within these modules. Importantly, several of these protein-coding genes were also associated with differentially expressed lncRNAs found within the Blue and Brown modules and some of these same genes were reported to have roles in the regulation of cell migration or in neural differentiation as well. For example, in the Blue module, *RDX* is a cytoskeletal protein linking the cytoplasm to the cell membrane^54^ that is also involved in regulating cell growth and migration^55^. Taken together, lncRNAs may be involved in the regulation of protein-coding genes related to cytoskeletal and membrane functions that can also underlie dysregulated neural cell motility and differentiation in depression and suicide.

Many lncRNAs in the Blue and Brown modules were also associated with genes (such as *PPFIA1*, *SNCB*, and *GRIN1*) involved in the development of neural circuitry and dendritic/synaptic morphology. Of note, SNCB has been previously implicated in anti-depressant response^56^ and *GRIN1* in depression^57^. Neuronal remodeling has been long known to be associated with depression^58^, and our data suggest that these processes may be regulated in part through lncRNAs.

Finally, several lncRNA-associated protein-coding genes (such as *TCF12* and *FNBP1*) are implicated in both neural processes involving cytoskeletal or membrane regulation as well as processing of immune signaling and function. Along with our findings that lncRNAs are associated with overlapping and antisense genes involved in IFN signaling, lncRNA associations with distal protein-coding genes also involved in immune and neurological processes implicate lncRNAs in regulating immune signals that may be significant players underlying depression and suicide.

We would like to acknowledge that a higher threshold value for module construction and detection was used than the recommended default parameters suggested by the developers of WGCNA (see Methods and Materials). This was done to increase the power of detecting significant modules after multiple testing corrections. While this approach reduces the specificity of gene networks, it would allow for the detection of broad significant networks of genes to provide an overall picture of the molecular and biological processes contributing to depression and suicide in the brain. Other studies taking a network approach to analyzing suicide related genes have treated suicide related genes as a “meta-system” of interacting pathways and processes and have similarly reported suicide related gene networks associated with signal transduction pathways, actin-interacting proteins, development/morphology phenotypes, and stress-sensitive synaptic plasticity processes^59^. While our results provide evidence in line with these previous findings, interpretation of the association of specific genes, including lncRNAs, need to be made with caution and further experiments are required to provide the evidence implicating their significance.

### Limitations and future directions

The present study is not without limitations. As it is often the case in studies of individuals who died by suicide, comorbidity is more the rule than the exception^60^. It is possible that some of the changes in the expression of specific lncRNAs identified in our study might not generalize to all cases of depression and suicide, given that factors such as history of early-life adversity or substance use may explain part of the variance. Future experiments, including animal models, should separate the relative contribution of comorbid factors.

Additionally, we recognize that the associations between lncRNAs and potential target genes need to be validated with additional experiments. Manipulation of lncRNA gene levels in vivo using animal models, or in vitro using cell lines, would confirm their effects on target genes, such as those identified from co-expression networks. For example, Bagot et al.^61^ showed that the overexpression of key network “hub genes” led to changes in the expression of a significant number of genes which were enriched with those belonging to the corresponding co-expression network that was related to depression susceptibility in mice. As lncRNA expression is tissue and cell-type-specific, utilizing the appropriate systems in which to probe lncRNA function becomes extremely important, but also challenging. Furthermore, in silico lncRNA target prediction tools can be used to supplement disease association approaches, such as ours, to improve target prediction^62^. Once promising lncRNA and target candidates have been narrowed down, targeted validation of RNA-RNA interactions, such as through RNA-interactome analysis (RIA-seq), can be used to identify direct binding partners of significant lncRNAs. The results can also help determine the accuracy of lncRNA target prediction approaches.

To conclude, to our knowledge, our study is the first to report a detailed and comprehensive list of lncRNAs expression in the brains of depressed individuals who died by suicide. We identified several lncRNAs that are differentially expressed in the rACC and found overlapping, antisense, and more distally located protein-coding genes significantly associated with these lncRNAs. Importantly, many of the lncRNA associated genes were implicated with immune processes, especially in IFN signaling, that may be significant signals driving biological and molecular changes in the brain. Altogether, our results uncover dysregulated lncRNAs in depression and suicide, and provide new molecular targets for follow-up post-mortem and in vitro functional studies.

## Electronic supplementary material

Supplementary Materials

Supplementary Materials Cont'd
