# Supplementary material for: Global long non-coding RNA expression in the rostral anterior cingulate cortex of depressed suicides
Source: Transl Psychiatry. 2018 Oct 18;8:224. doi: 10.1038/s41398-018-0267-7 (PMC6193959; doi:10.1038/s41398-018-0267-7)
Supplement: Supplementary file 1 — Supplementary Materials [file 41398_2018_267_MOESM1_ESM.docx]

**Supplementary Materials**

Supplementary Methods

Full Transcriptome Differential Expression Analysis:

**Alignment.** Following high-throughput sequencing, 100bp paired-end reads were aligned to the hg19 human genome using TopHat v2.1.0 (http://tophat.cbcb.umd.edu/) with a mate insert distance of 75 bp (-r) and library type fr-unstranded. Reads passing a mapping quality of at least 50 were used for gene and transcript quantification.

**Quantification**. Gene annotations from the Ensembl release 75 were used for quantification. For gene-level quantification we used HTSeq-count version 0.6.1p1 (http://www-huber.embl.de/users/anders/HTSeq/doc/overview.html) (23), using the intersection-nonempty mode, and results were combined to form a count matrix of 20,893 transcribed RNAs across 50 samples

**Differential expression analysis.** Genes with no mapped fragments were removed from the analysis. Furthermore, genes with low counts were removed by keeping only those with at least 10 counts per subject on average. Differential expression analysis was performed using the DESeq2 GLM using the following covariates: gender (24), age (25), and RIN (26), based on previous literature documenting their impact on human brain RNA-Seq datasets.

Supplementary Figure Legends

**Supplementary Figure 1** – With the guidance of a human brain atlas, the rACC was dissected in sections equivalent to plate 6 (-30mm from the center of the anterior commissure approximately) of the atlas. Grey matter was taken immediately rostral to corpus callosum genu. The rostral landmark limit was the cingulate sulcus, and the caudal landmark limit was the callosal sulcus.

**Supplementary Figure 2 – A)** Principal component analysis (PCA) exploring subject sex, **B)** PCA exploring subject sex with long non-coding RNA *XIST* removed, and **C)** hierarchical clustering in samples.

**Supplementary Figure 3** – (a-m) RT-qPCR validation of differentially expressed long non-coding RNAs (lncRNAs). Estimated marginal means are reported and gene expression was normalized to the geometric mean of three housekeeping genes: *ARHGEF12, TUBA1A*, and *B-Actin*.

**Supplementary Figure 4 –** Hierarchical clustering and branch cutting implemented by Weighted Gene Co-expression Network Analysis to construct co-expression networks using 21, 000 genes.

Supplementary Table Legends

**Supplementary Table 1 –** Sample demographics and characteristics. Means and standard errors are reported.

**Supplementary Table 2 –** qPCR primer sequences.

**Supplementary Table 3 –** Stability of housekeeping genes in RNA-seq and qPCR data between controls and depressed suicides.

**Supplementary Table 4** – **A)** Proportions of samples showing toxicological evidence for the presence medications at the time of death. **B)** Correlations between presence of benzodiazepines and acetaminophen and the top 4 principal components from the RNA-seq lncRNA expression data.

**Supplementary Table 5 –** Effects of including the presence of **A)** Benzodiazepine and **B)** Acetaminophen at the time of death as co-variates in the GLM analysis of the qPCR validation of differential lncRNA expression.

**Supplementary Table 6 –** Correlations between normalized RNA-seq expression values and RT-qPCR expression values (ΔCt: lncRNA_Ct_ – HKG_GeoMeanCt_) across 50 samples for each lncRNA. P-values highlighted in red are < 0.05 and p-values highlighted in yellow correlations that remain significant after Bonferonni corrections.

**Supplementary Table 7 -** Summary of differential expression analysis of genes antisense and overlapping to lncRNA using RNA-seq expression data. *baseMean* represents the normalized read counts for each gene, *log2FoldChange* represents a log transformed fold change value, *lfcSE* represents log fold change standard error, and *stat* represents the test-statstic, *p-value* represents the un-corrected significance value, and *p-adj* represents the corrected significance value (FDR < 0.1).

**Supplementary Table 8 –** F-statistics and corresponding p-values for each sample characteristic or trait, corresponding to each co-expression module, are shown. Benjamini-Hochberg multiple comparisons corrections were implemented for the analysis of the Group factor with a false discovery rate (FDR) of less than 0.1. Significant modules are highlighted in white while non-significant ones are highlighted in green. Note that the grey “module” contains genes not assigned to any modules.

**Supplementary Table 9 –** Validated lncRNA and module membership (MM) values corresponding to each lncRNA’s assigned module are shown. MM is the correlation between the expression of the gene and the Module Eigengene (ME). The ME is a principal component that represents the overall gene expression profile of each module. The larger the MM value, the higher the connectivity to other genes in the network.

**Supplementary Table 10** – Differential expression results for 2670 lncRNAs detected by RNA-seq.

**Supplementary Figure 1 –**


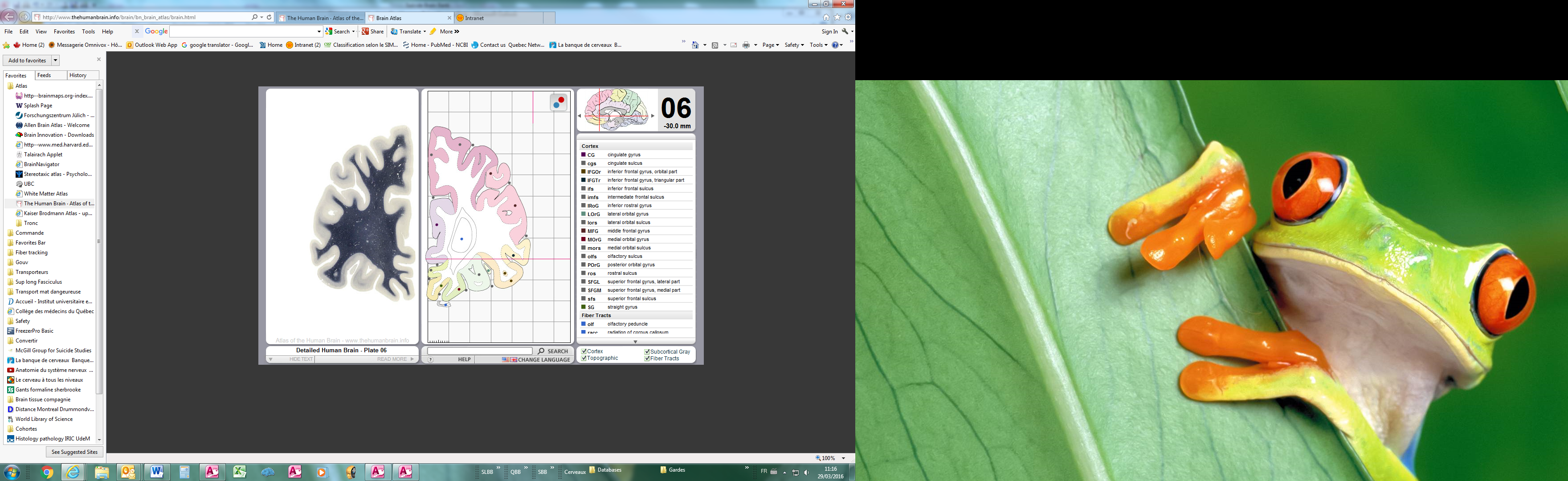


**Supplementary Figure 2 -**

**A) B)**

*
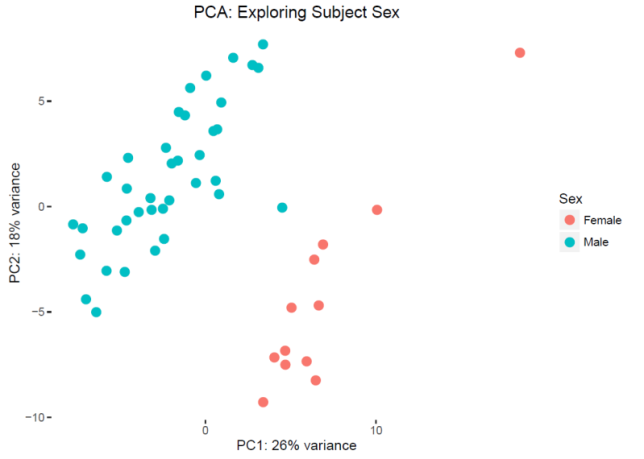
* *
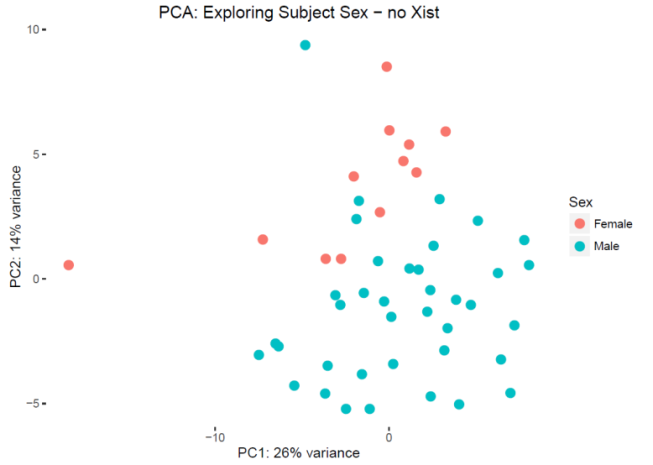
*

**C)**


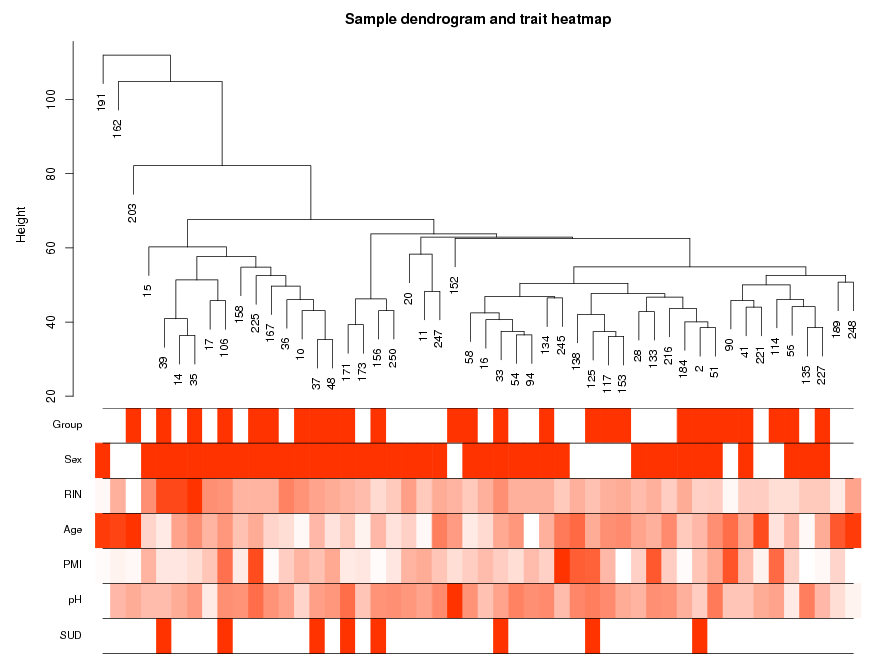


**Supplementary Figure 3 –**

a) b)

c)d)

e)f)

g)h)

i)j)

k)l)

m)

**Supplementary Figure 4 -**


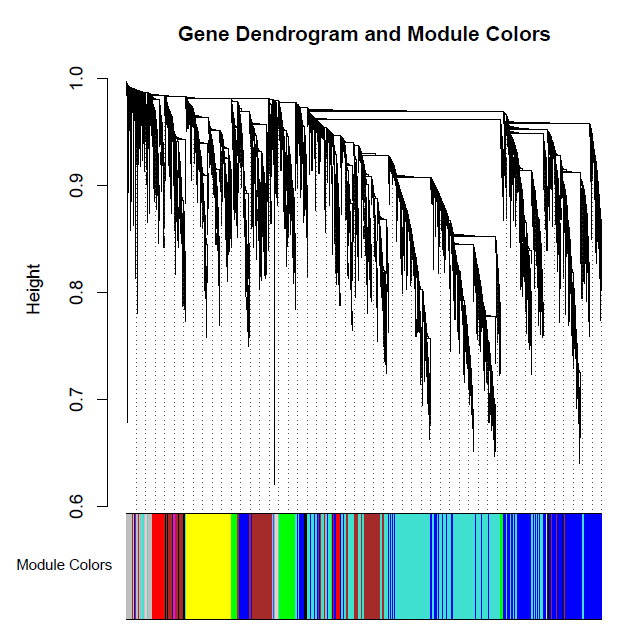


**Supplementary Table 1**

| **Rostral Anterior Cingulate Cortex** | | | |
| --- | --- | --- | --- |
| **Characteristics** | Controls | Depressed/Suicide | p-value |
| **N** | 24 | 26 | n/a |
| **Age (years)** | 46.75 ± 4.45 | 42.23 ± 2.84 | 0.389 |
| **PMI (hours)** | 22.13 ± 3.58 | 25.21 ± 3.88 | 0.563 |
| **Brain pH** | 6.47 ± 0.06 | 6.61 ± 0.06 | 0.114 |
| **RIN** | 6.86 ± 0.12 | 6.83 ± 0.15 | 0.859 |
| **Male/Female** | 19/5 | 19/7 | 0.614 |
| **Mood Disorder** | 0 (0%) | 26 (100%) | <0.0001 |
| **Substance Use Disorder** | 0 (0%) | 8 (21%) | 0.003 |

**Supplementary Table 2**

| **Gene** | **Forward Primer (5’ – 3’)** | | | **Reverse Primer** | **Product (bp)** |
| --- | --- | --- | --- | --- | --- |
| RP11-453F18_B.1 | ACCAAAACCGAGTGAAGAGGA | | | TCAGGTCTCACATCACATAAGTCTC | 180 |
| RP11-96D1.10 | GCAAGGAATGAAATGATGTGAGA | | | AAGCATCTCAGGGAGGTACAG | 134 |
| RP1-269M15.3 | GAGGGGTGTAAAGGACTGGG | | | TTCAAACATTGCAGAGGCGG | 153 |
| C9orf106 | GAGAAACTGCTGAGACGAGGT | | | CCGACAGGTGAGGTTTATCGC | 81 |
| CTC-487M23.5 | GGTCCAAGCACTCCAAGGAA | | | TGGGTCAATGCAAATGCGTA | 198 |
| CTD-2647L4.4 | GCAAGGGTCATCTGTCAGGATG | | | GGCGGTCATCTTTGAAACCAA | 113 |
| RP11-1391J7.1 | GGCAGGAAGGAACCCTACAC | | | GTCACAGTGTGTGTGACCGA | 172 |
| RP11-143K11.5 | GTGTCGGAGACCGGAGCTA | | | TGCTGAATCTTGTACCTGGGC | 129 |
| RP11-273G15.2 | AGTGTATCACAGCCATTGGGA | | | AGGACGCTTCGGTCTCTGTA | 151 |
| RP11-326I11.3 | CTGCGACCATCCAGTTAGCA | | | CAGCCACGGATAAGGTTCCA | 155 |
| RP11-326I11.5 | GCTGTGACTCGGTCTGCTTT | | | CGGTGATGCCTGAAAATCCAC | 85 |
| RP11-434C1.1 | TTTCTCCTGTCTCCTCCCCAA | | | CTGACACTTCTGCCACTGGTT | 245 |
| ZNF833P | ACTCAGTGACCTTTGAGGATG | | | TTCCTTACCTTCAGACTCTCTCTAC | 161 |
| *Antisense and Overlapping Protein Coding Genes* | | | | | |
| HMBOX1 | AACAGCATGGGTCAGAGGTC | | | CTGTGCAACAACTGCTTGGG | 162 |
| LY6E | TCTGTACTGCCTGAAGCCGA | | | CATGGAAGCCACACCAACAT | 169 |
| IRF2 | TCCATACAGGAAAGCATCAACC | | | CATGGCGCATCTGAAATTCGC | 77 |
| *housekeeping* |  | | |  |  |
| TUBA1A | GCTGCCAATAACTATGCCCG | | | AAACCAAGAAGCCCTGGAGAC | 118 |
| ACTB | CCGTCTTCCCCTCCATCGT | | | GGATGCCTCTCTTGCTCTGG | 108 |
| ARHGEF12 | ACACAGTCTACTATCACCGACA | | | TGCAATGCGCTCAACTTTCTG | 111 |
|  |  | | |  |  |
|  |  | | |  |  |
|  |  | | |  |  |
|  | |  |  |  |  |
|  | |  |  |  |  |
|  | |  |  |  |  |
|  | |  |  |  |  |
|  | |  |  |  |  |
|  | |  |  |  |  |
|  | |  |  |  |  |

**Supplementary Table 3**

**Supplementary Table 4**

**A)**

**B)**

**Supplementary Table 5**

**A)**

**5A) cont’d**

**B)**

**5B) cont’d**

**Supplementary Table 6**

| lncRNA | Pearson r | 95% CI | P value (two-tailed) | Bonferonni = 0.004 |
| --- | --- | --- | --- | --- |
| C9orf106 | -0.6506 | -0.7865 to -0.4545 | < 0.0001 | < 0.0001 |
| CTC-487M23.5 | -0.5285 | -0.7034 to -0.2932 | < 0.0001 | < 0.0001 |
| CTD-2647L4.4 | -0.3882 | -0.6016 to -0.1231 | 0.0053 | 0.0053 |
| FIRRE/RP11-453F18__B.1 | -0.6395 | -0.7792 to -0.4393 | < 0.0001 | < 0.0001 |
| RP11-1391J7.1 | -0.2453 | -0.4902 to 0.03557 | 0.086 | 0.086 |
| RP11-143K11.5 | -0.5352 | -0.7081 to -0.3018 | < 0.0001 | < 0.0001 |
| RP11-273G15.2 | -0.6875 | -0.8107 to -0.5059 | < 0.0001 | < 0.0001 |
| RP11-326I11.3 | -0.6775 | -0.8042 to -0.4918 | < 0.0001 | < 0.0001 |
| RP11-326I11.5 | -0.4206 | -0.6257 to -0.1610 | 0.0024 | 0.0024 |
| RP11-434C1.1 | -0.5828 | -0.7410 to -0.3634 | < 0.0001 | < 0.0001 |
| RP11-96D1.10 | -0.3929 | -0.6051 to -0.1285 | 0.0048 | 0.0048 |
| RP1-269M15.3 | -0.7039 | -0.8214 to -0.5292 | < 0.0001 | < 0.0001 |
| ZNF833P | -0.7973 | -0.8803 to -0.6669 | < 0.0001 | < 0.0001 |

**Supplementary Table 7**

**Supplementary Table 8**

| Module | RIN | Age | PMI | pH | Sex | SUD | Group | Group p-adj (FDR < 0.1) |
| --- | --- | --- | --- | --- | --- | --- | --- | --- |
| Brown | 47.75 | 1.44 | 0.07 | 0.71 | 0.85 | 2.14 | 9.41 |  |
| *p-value* | *0.000* | *0.237* | *0.790* | *0.405* | *0.361* | *0.151* | *0.004* | ***0.044*** |
| Blue | 6.68 | 0.18 | 2.55 | 0.31 | 0.50 | 1.50 | 7.00 |  |
| *p-value* | *0.010* | *0.670* | *0.120* | *0.580* | *0.490* | *0.230* | *0.010* | ***0.061*** |
| Black | 2.84 | 1.91 | 1.21 | 1.86 | 1.62 | 0.01 | 4.18 |  |
| *p-value* | *0.099* | *0.174* | *0.278* | *0.179* | *0.210* | *0.943* | *0.047* | *0.172* |
| Pink | 0.00 | 16.26 | 1.05 | 1.10 | 0.00 | 1.84 | 2.19 |  |
| *p-value* | *0.983* | *0.000* | *0.311* | *0.301* | *0.978* | *0.182* | *0.147* | *0.404* |
| Magenta | 29.66 | 2.34 | 0.97 | 0.03 | 0.03 | 0.04 | 1.39 |  |
| *p-value* | *0.000* | *0.134* | *0.330* | *0.875* | *0.870* | *0.836* | *0.245* | *0.539* |
| Yellow | 1.04 | 0.99 | 9.15 | 6.25 | 1.59 | 3.11 | 1.33 |  |
| *p-value* | *0.313* | *0.325* | *0.004* | *0.016* | *0.215* | *0.085* | *0.255* | *0.468* |
| Turquoise | 97.78 | 1.83 | 1.99 | 0.25 | 1.52 | 0.03 | 1.11 |  |
| *p-value* | *0.000* | *0.183* | *0.166* | *0.618* | *0.225* | *0.873* | *0.297* | *0.467* |
| Red | 6.77 | 3.60 | 0.04 | 3.50 | 2.77 | 0.07 | 0.35 |  |
| *p-value* | *0.013* | *0.065* | *0.843* | *0.068* | *0.103* | *0.800* | *0.558* | *0.767* |
| Purple | 1.54 | 0.44 | 4.25 | 3.76 | 2430.71 | 0.61 | 0.21 |  |
| *p-value* | *0.222* | *0.512* | *0.046* | *0.059* | *0.000* | *0.439* | *0.646* | *0.790* |
| Green | 10.32 | 8.34 | 1.49 | 1.19 | 1.41 | 0.15 | 0.08 |  |
| *p-value* | *0.003* | *0.006* | *0.229* | *0.282* | *0.241* | *0.704* | *0.775* | *0.853* |
| Grey | 0.47 | 1.58 | 0.33 | 0.30 | 0.31 | 1.80 | 0.02 |  |
| *p-value* | *0.499* | *0.216* | *0.567* | *0.590* | *0.582* | *0.187* | *0.895* | *0.895* |

**Supplementary Table 9**

| Validated lncRNA | Module | MM | MM p-value |
| --- | --- | --- | --- |
| CTC-487M23.5 | blue | 0.76 | 1.53E-10 |
| CTD-2647L4.4 | blue | 0.57 | 1.68E-05 |
| RP11-453F18_B.1 | blue | 0.74 | 6.75E-10 |
| RP11-273G15.2 | blue | -0.68 | 6.18E-08 |
| RP11-326I11.3 | blue | 0.83 | 1.28E-13 |
| RP1-269M15.3 | blue | -0.81 | 7.71E-13 |
| RP11-434C1.1 | brown | 0.56 | 2.61E-05 |
| RP11-96D1.10 | turquoise | -0.52 | 1.05E-04 |
| ZNF833P | turquoise | 0.33 | 0.02019 |

**Supplementary Table 10**

| Gene | log2FoldChange | lfcSE | stat | pvalue | padj |
| --- | --- | --- | --- | --- | --- |
| SNORD3C | 0.659740803 | 0.123503 | 5.341908 | 9.20E-08 | 0.000212 |
| RP11-453F18__B.1 | -0.554397449 | 0.121995 | -4.54444 | 5.51E-06 | 0.006354 |
| LLNLF-65H9.1 | 0.426305939 | 0.099675 | 4.276947 | 1.89E-05 | 0.014571 |
| RP11-96D1.10 | -0.32179568 | 0.081819 | -3.93301 | 8.39E-05 | 0.048383 |
| RP1-269M15.3 | 0.363252411 | 0.095875 | 3.7888 | 0.000151 | 0.069845 |
| AC004019.18/FLJ41941 | -0.40855476 | 0.118413 | -3.45026 | 0.00056 | 0.097802 |
| AC006003.3 | 0.366093815 | 0.110382 | 3.316604 | 0.000911 | 0.097802 |
| AC012507.3 | 0.341590103 | 0.097979 | 3.486362 | 0.00049 | 0.097802 |
| AC013460.1 | 0.369468169 | 0.110936 | 3.330449 | 0.000867 | 0.097802 |
| C9orf106 | 0.339831021 | 0.102477 | 3.316175 | 0.000913 | 0.097802 |
| CTC-487M23.5 | -0.378393959 | 0.111085 | -3.40635 | 0.000658 | 0.097802 |
| CTD-2647L4.4 | -0.25565737 | 0.071639 | -3.56871 | 0.000359 | 0.097802 |
| DYX1C1-CCPG1 | 0.162690891 | 0.045212 | 3.598416 | 0.00032 | 0.097802 |
| RP1-63G5.5 | 0.25483405 | 0.075689 | 3.366855 | 0.00076 | 0.097802 |
| RP11-1186N24.5 | 0.232686736 | 0.070314 | 3.309239 | 0.000935 | 0.097802 |
| RP11-1391J7.1 | 0.315266004 | 0.092954 | 3.391647 | 0.000695 | 0.097802 |
| RP11-143K11.5 | 0.335609071 | 0.099189 | 3.383517 | 0.000716 | 0.097802 |
| RP11-273G15.2 | 0.275172908 | 0.08311 | 3.310965 | 0.00093 | 0.097802 |
| RP11-326G21.1 | -0.454562316 | 0.136384 | -3.33297 | 0.000859 | 0.097802 |
| RP11-326I11.3 | -0.360695913 | 0.099962 | -3.60834 | 0.000308 | 0.097802 |
| RP11-326I11.5 | -0.325321295 | 0.096016 | -3.38821 | 0.000704 | 0.097802 |
| RP11-434C1.1 | 0.252907456 | 0.075125 | 3.366469 | 0.000761 | 0.097802 |
| ZNF833P | 0.263513644 | 0.07991 | 3.297628 | 0.000975 | 0.097802 |
| RP1-63G5.7 | 0.38061142 | 0.117823 | 3.230361 | 0.001236 | 0.101865 |
| RP11-466F5.8 | -0.288732294 | 0.089321 | -3.23252 | 0.001227 | 0.101865 |
| RP11-497H16.9 | -0.355721566 | 0.109213 | -3.25715 | 0.001125 | 0.101865 |
| RP11-682N22.1 | -0.306668403 | 0.094334 | -3.25089 | 0.00115 | 0.101865 |
| RP11-794G24.1 | 0.323584831 | 0.099888 | 3.239489 | 0.001197 | 0.101865 |
| PSMG3-AS1 | 0.268094749 | 0.083503 | 3.210619 | 0.001324 | 0.104306 |
| RP11-513O17.2 | 0.338781286 | 0.105909 | 3.198785 | 0.00138 | 0.104306 |
| RP11-712P20.2 | -0.308620233 | 0.096615 | -3.19432 | 0.001402 | 0.104306 |
| TRAF3IP2-AS1 | -0.152436355 | 0.048139 | -3.16662 | 0.001542 | 0.111185 |
| AC018737.1 | -0.326780956 | 0.107119 | -3.05063 | 0.002284 | 0.123696 |
| CTC-203F4.2 | -0.390411366 | 0.125237 | -3.11738 | 0.001825 | 0.123696 |
| CTC-459F4.3 | 0.203181127 | 0.066162 | 3.070976 | 0.002134 | 0.123696 |
| CTD-2521M24.9 | -0.332512854 | 0.107747 | -3.08604 | 0.002028 | 0.123696 |
| PTOV1-AS1 | -0.344352794 | 0.111529 | -3.08755 | 0.002018 | 0.123696 |
| RP11-1007O24.3 | -0.262773027 | 0.086154 | -3.05004 | 0.002288 | 0.123696 |
| RP11-119F7.5 | -0.317146866 | 0.101441 | -3.12643 | 0.001769 | 0.123696 |
| RP11-438N16.1 | 0.291822347 | 0.094315 | 3.094133 | 0.001974 | 0.123696 |
| RP11-452F19.3 | -0.287415497 | 0.093981 | -3.05824 | 0.002226 | 0.123696 |
| ZMIZ1-AS1 | 0.301156974 | 0.098574 | 3.055137 | 0.00225 | 0.123696 |
| ZNRD1-AS1 | -0.182422037 | 0.059855 | -3.04776 | 0.002306 | 0.123696 |
| MAGI2-AS3 | -0.22101065 | 0.072869 | -3.03297 | 0.002422 | 0.124148 |
| RP11-783K16.10 | 0.265272016 | 0.0874 | 3.035161 | 0.002404 | 0.124148 |
| DLGAP1-AS1 | -0.343400083 | 0.115401 | -2.97571 | 0.002923 | 0.130436 |
| KCNJ2-AS1 | -0.379377062 | 0.127231 | -2.98181 | 0.002866 | 0.130436 |
| RP11-142O6.1 | -0.242258774 | 0.081634 | -2.9676 | 0.003001 | 0.130436 |
| RP11-206L10.3 | -0.258434331 | 0.086507 | -2.98745 | 0.002813 | 0.130436 |
| RP11-212I21.4 | -0.385465381 | 0.128856 | -2.99145 | 0.002777 | 0.130436 |
| RP11-344E13.3 | 0.325504739 | 0.108255 | 3.00683 | 0.00264 | 0.130436 |
| RP11-680F8.1 | 0.280878596 | 0.094074 | 2.985729 | 0.002829 | 0.130436 |
| RP11-830F9.7 | 0.318354619 | 0.107467 | 2.962338 | 0.003053 | 0.130436 |
| RP11-848P1.2 | -0.336687459 | 0.113575 | -2.96445 | 0.003032 | 0.130436 |
| RP11-278H7.1 | 0.275990319 | 0.094097 | 2.933033 | 0.003357 | 0.135857 |
| RP11-420N3.2 | 0.234836826 | 0.079769 | 2.943945 | 0.003241 | 0.135857 |
| SOX2-OT | -0.378555303 | 0.128924 | -2.93626 | 0.003322 | 0.135857 |
| PPP2R2B-IT1 | -0.362758936 | 0.125944 | -2.88031 | 0.003973 | 0.155375 |
| SLC2A1-AS1 | 0.224668666 | 0.078103 | 2.876572 | 0.00402 | 0.155375 |
| TNRC6C-AS1 | -0.37562772 | 0.130656 | -2.87495 | 0.004041 | 0.155375 |
| LINC00460 | 0.364188962 | 0.126965 | 2.868421 | 0.004125 | 0.156016 |
| ZRANB2-AS2 | -0.285327245 | 0.099979 | -2.85387 | 0.004319 | 0.160711 |
| SRRM2-AS1 | -0.258351963 | 0.091469 | -2.82447 | 0.004736 | 0.173422 |
| AC141928.1 | 0.232103782 | 0.08645 | 2.684824 | 0.007257 | 0.175688 |
| AC159540.1 | 0.196162805 | 0.070956 | 2.764557 | 0.0057 | 0.175688 |
| ADIRF-AS1 | 0.261079124 | 0.096795 | 2.697225 | 0.006992 | 0.175688 |
| AP000473.8 | -0.253479286 | 0.094893 | -2.67122 | 0.007558 | 0.175688 |
| CDKN2B-AS1 | -0.296055931 | 0.109285 | -2.70903 | 0.006748 | 0.175688 |
| CTB-131B5.5 | 0.278900027 | 0.103928 | 2.683601 | 0.007283 | 0.175688 |
| CTB-55O6.12 | -0.333996509 | 0.123966 | -2.69425 | 0.007055 | 0.175688 |
| CTC-428H11.2 | -0.27605956 | 0.103675 | -2.66273 | 0.007751 | 0.175688 |
| CTC-462L7.1 | -0.26803643 | 0.099506 | -2.69368 | 0.007067 | 0.175688 |
| CTD-2514K5.2 | -0.303607322 | 0.112076 | -2.70894 | 0.00675 | 0.175688 |
| DLEU2 | -0.281971611 | 0.101913 | -2.76678 | 0.005661 | 0.175688 |
| KRTAP5-AS1 | 0.249820917 | 0.093201 | 2.680465 | 0.007352 | 0.175688 |
| LINC00472 | -0.269546052 | 0.100284 | -2.68782 | 0.007192 | 0.175688 |
| LINC00844 | -0.36984604 | 0.136782 | -2.70391 | 0.006853 | 0.175688 |
| LINC00938 | -0.212955613 | 0.07651 | -2.78336 | 0.00538 | 0.175688 |
| LY86-AS1 | 0.234259561 | 0.084868 | 2.760272 | 0.005775 | 0.175688 |
| NNT-AS1 | -0.138322334 | 0.051717 | -2.67462 | 0.007481 | 0.175688 |
| RAPGEF4-AS1 | 0.224926852 | 0.081661 | 2.75441 | 0.00588 | 0.175688 |
| RP11-121C2.2 | -0.175958399 | 0.065672 | -2.67935 | 0.007377 | 0.175688 |
| RP11-127I20.7 | 0.243676864 | 0.088321 | 2.758998 | 0.005798 | 0.175688 |
| RP11-168O16.1 | -0.264427483 | 0.099384 | -2.66066 | 0.007799 | 0.175688 |
| RP11-196G18.22 | -0.214875731 | 0.080819 | -2.65872 | 0.007844 | 0.175688 |
| RP11-250B2.5 | -0.272291559 | 0.09766 | -2.78817 | 0.005301 | 0.175688 |
| RP11-274B21.9 | 0.256666329 | 0.093532 | 2.744157 | 0.006067 | 0.175688 |
| RP11-286E11.1 | -0.349512865 | 0.128978 | -2.70986 | 0.006731 | 0.175688 |
| RP11-3B12.1 | -0.207905193 | 0.076729 | -2.70959 | 0.006737 | 0.175688 |
| RP11-466P24.7 | -0.296395543 | 0.110738 | -2.67654 | 0.007439 | 0.175688 |
| RP11-497E19.1 | 0.271087448 | 0.098449 | 2.75358 | 0.005895 | 0.175688 |
| RP11-506M13.3 | -0.248011525 | 0.09223 | -2.68907 | 0.007165 | 0.175688 |
| RP11-563J2.2 | -0.320896092 | 0.115926 | -2.7681 | 0.005638 | 0.175688 |
| RP11-84A19.4 | -0.337946308 | 0.124929 | -2.7051 | 0.006828 | 0.175688 |
| RP11-946L20.2 | -0.377092896 | 0.135201 | -2.78912 | 0.005285 | 0.175688 |
| RP4-613B23.1 | -0.355591451 | 0.131332 | -2.70757 | 0.006778 | 0.175688 |
| RP4-717I23.3 | -0.215124595 | 0.080388 | -2.67609 | 0.007449 | 0.175688 |
| RP5-827C21.2 | 0.280636014 | 0.104974 | 2.673385 | 0.007509 | 0.175688 |
| RP5-944M2.3 | 0.322313139 | 0.121179 | 2.659813 | 0.007818 | 0.175688 |
| RP6-91H8.1 | 0.291619756 | 0.107865 | 2.70357 | 0.00686 | 0.175688 |
| SCARNA15 | -0.214738215 | 0.080637 | -2.66302 | 0.007744 | 0.175688 |
| TUG1 | -0.148173574 | 0.055321 | -2.67843 | 0.007397 | 0.175688 |
| ZNF252P-AS1 | -0.259414526 | 0.092903 | -2.79232 | 0.005233 | 0.175688 |
| CTA-268H5.14 | 0.287633969 | 0.109444 | 2.628144 | 0.008585 | 0.17684 |
| PAN3-AS1 | -0.226834795 | 0.086057 | -2.63585 | 0.008393 | 0.17684 |
| PROX1-AS1 | -0.299373388 | 0.113075 | -2.64756 | 0.008108 | 0.17684 |
| RP11-34P13.13 | -0.309841181 | 0.117796 | -2.63032 | 0.00853 | 0.17684 |
| RP11-38L15.3 | 0.238663784 | 0.089987 | 2.652193 | 0.007997 | 0.17684 |
| RP11-545I5.3 | 0.135520129 | 0.051537 | 2.629592 | 0.008549 | 0.17684 |
| RP11-553L6.5 | -0.308961807 | 0.11696 | -2.6416 | 0.008251 | 0.17684 |
| RP11-572C15.6 | -0.311372932 | 0.117899 | -2.64101 | 0.008266 | 0.17684 |
| RP3-399L15.3 | -0.29592684 | 0.112517 | -2.63005 | 0.008537 | 0.17684 |
| AC138035.2 | -0.267384497 | 0.102501 | -2.6086 | 0.009091 | 0.181876 |
| ANKRD44-IT1 | -0.291974667 | 0.112267 | -2.60072 | 0.009303 | 0.181876 |
| RP11-164J13.1 | -0.350494325 | 0.134717 | -2.60172 | 0.009276 | 0.181876 |
| RP11-228B15.4 | -0.259637188 | 0.099822 | -2.601 | 0.009295 | 0.181876 |
| RP11-356J5.12 | 0.194303492 | 0.074585 | 2.605113 | 0.009184 | 0.181876 |
| RP11-760H22.2 | -0.293607493 | 0.112579 | -2.608 | 0.009107 | 0.181876 |
| GS1-124K5.3 | -0.254305382 | 0.097927 | -2.5969 | 0.009407 | 0.182369 |
| RP11-261C10.3 | -0.192476573 | 0.074488 | -2.584 | 0.009766 | 0.187755 |
| RP5-855F14.1 | -0.332732649 | 0.128952 | -2.58028 | 0.009872 | 0.18822 |
| AC004603.4 | -0.317303476 | 0.123384 | -2.57168 | 0.010121 | 0.191377 |
| AC067956.1 | 0.195991895 | 0.076627 | 2.557726 | 0.010536 | 0.195263 |
| AF011889.2 | 0.227676725 | 0.089636 | 2.540017 | 0.011085 | 0.195263 |
| CTC-457E21.1 | -0.346389063 | 0.135958 | -2.54777 | 0.010841 | 0.195263 |
| LINC00899 | -0.30179825 | 0.118479 | -2.54728 | 0.010857 | 0.195263 |
| RP11-116N8.1 | -0.348893062 | 0.137132 | -2.54422 | 0.010952 | 0.195263 |
| RP11-143A12.3 | -0.348390349 | 0.137027 | -2.54249 | 0.011006 | 0.195263 |
| RP11-29G8.3 | 0.173790783 | 0.068424 | 2.53992 | 0.011088 | 0.195263 |
| RP11-845M18.6 | 0.277911792 | 0.109309 | 2.542435 | 0.011008 | 0.195263 |
| RP4-612B15.3 | 0.259919342 | 0.101589 | 2.55854 | 0.010511 | 0.195263 |
| LINC00609 | -0.345494907 | 0.136542 | -2.53032 | 0.011396 | 0.197673 |
| MANEA-AS1 | -0.246003226 | 0.097176 | -2.53153 | 0.011357 | 0.197673 |
| AC007246.3 | 0.168438658 | 0.067058 | 2.511839 | 0.01201 | 0.197914 |
| AC013394.2 | -0.183127252 | 0.07282 | -2.5148 | 0.01191 | 0.197914 |
| CTD-3195I5.1 | -0.203566673 | 0.080679 | -2.52317 | 0.01163 | 0.197914 |
| LINC00341 | -0.314856541 | 0.125214 | -2.51454 | 0.011919 | 0.197914 |
| RP11-282O18.3 | -0.246697527 | 0.098196 | -2.5123 | 0.011995 | 0.197914 |
| RP11-673C5.2 | 0.261408879 | 0.103686 | 2.521161 | 0.011697 | 0.197914 |
| RSF1-IT2 | -0.247810717 | 0.098507 | -2.51567 | 0.011881 | 0.197914 |
| RP11-488L18.10 | -0.2082887 | 0.083046 | -2.50811 | 0.012138 | 0.198594 |
| OPCML-IT1 | 0.264336721 | 0.105588 | 2.503463 | 0.012298 | 0.199806 |
| SEMA3B | -0.339358151 | 0.135963 | -2.49596 | 0.012562 | 0.200968 |
| SOX21-AS1 | -0.273832054 | 0.109796 | -2.494 | 0.012631 | 0.200968 |
| SRP14-AS1 | -0.281241526 | 0.112624 | -2.49718 | 0.012518 | 0.200968 |
| RP13-977J11.2 | 0.27705232 | 0.111215 | 2.491136 | 0.012734 | 0.201207 |
| CAHM | -0.2675687 | 0.107846 | -2.48104 | 0.0131 | 0.202971 |
| SATB2-AS1 | 0.270086029 | 0.108871 | 2.480791 | 0.013109 | 0.202971 |
| ZNF883 | 0.179694999 | 0.072389 | 2.482356 | 0.013052 | 0.202971 |
| RP11-263K19.6 | 0.229207997 | 0.092527 | 2.47719 | 0.013242 | 0.203664 |
| CTD-2619J13.9 | 0.195583785 | 0.079035 | 2.474651 | 0.013337 | 0.203759 |
| RP11-243A14.1 | -0.3253749 | 0.131772 | -2.46922 | 0.013541 | 0.205515 |
| RP11-261N11.8 | 0.22841391 | 0.092711 | 2.463728 | 0.01375 | 0.207329 |
| LINC00936 | 0.192885967 | 0.078388 | 2.460654 | 0.013868 | 0.207756 |
| LINC00087 | 0.217049341 | 0.088304 | 2.45797 | 0.013972 | 0.207965 |
| RP1-30M3.5 | -0.223109791 | 0.090965 | -2.45269 | 0.014179 | 0.209691 |
| RP11-340F14.5 | -0.27624732 | 0.112752 | -2.45005 | 0.014284 | 0.209887 |
| RP11-263K19.4 | -0.269301194 | 0.110278 | -2.44202 | 0.014605 | 0.213254 |
| RP11-958J22.1 | 0.252416994 | 0.103532 | 2.438058 | 0.014766 | 0.214252 |
| LL22NC03-13G6.2 | 0.319475712 | 0.131772 | 2.424454 | 0.015331 | 0.216991 |
| RP1-34H18.1 | 0.166281351 | 0.068531 | 2.426367 | 0.015251 | 0.216991 |
| RP11-299J3.8 | -0.279742649 | 0.115375 | -2.42465 | 0.015323 | 0.216991 |
| RP11-99J16__A.2 | -0.275555909 | 0.113438 | -2.42913 | 0.015135 | 0.216991 |
| HOXD-AS1 | -0.322240416 | 0.133266 | -2.41802 | 0.015605 | 0.21819 |
| RP11-305K5.1 | -0.199339565 | 0.08241 | -2.41889 | 0.015568 | 0.21819 |
| AC097500.2 | -0.185641498 | 0.076991 | -2.4112 | 0.0159 | 0.218676 |
| CCDC147-AS1 | 0.222731276 | 0.092395 | 2.410644 | 0.015924 | 0.218676 |
| RP11-355B11.2 | -0.228020119 | 0.094463 | -2.41386 | 0.015784 | 0.218676 |
| AC007036.5 | -0.282444957 | 0.117318 | -2.40751 | 0.016062 | 0.219258 |
| RP11-25K21.6 | -0.327255922 | 0.136705 | -2.39388 | 0.016671 | 0.22188 |
| RP11-349A22.5 | -0.121679625 | 0.050878 | -2.39158 | 0.016776 | 0.22188 |
| RP11-386G11.5 | 0.249259318 | 0.104043 | 2.395743 | 0.016587 | 0.22188 |
| RP11-395B7.7 | -0.275883132 | 0.115105 | -2.3968 | 0.016539 | 0.22188 |
| RP11-792D21.2 | -0.289884475 | 0.120878 | -2.39816 | 0.016478 | 0.22188 |
| RP5-1057J7.6 | -0.186790174 | 0.078142 | -2.39038 | 0.016831 | 0.22188 |
| AC005329.7 | -0.308186723 | 0.130464 | -2.36223 | 0.018165 | 0.223834 |
| AC074289.1 | -0.258896936 | 0.109721 | -2.35959 | 0.018295 | 0.223834 |
| AC114765.1 | 0.233339377 | 0.098803 | 2.361658 | 0.018193 | 0.223834 |
| CTC-251D13.1 | 0.234086916 | 0.098793 | 2.36946 | 0.017814 | 0.223834 |
| CTD-2574D22.4 | -0.164746634 | 0.069642 | -2.36562 | 0.018 | 0.223834 |
| IL12A-AS1 | 0.252306622 | 0.107056 | 2.356773 | 0.018435 | 0.223834 |
| RP11-154D6.1 | -0.248655471 | 0.104729 | -2.37428 | 0.017583 | 0.223834 |
| RP11-267C16.1 | -0.316824279 | 0.133961 | -2.36505 | 0.018028 | 0.223834 |
| RP11-44N21.4 | 0.201280741 | 0.084676 | 2.37706 | 0.017451 | 0.223834 |
| RP11-548B3.3 | 0.183294075 | 0.077017 | 2.379906 | 0.017317 | 0.223834 |
| RP11-582J16.4 | -0.283260039 | 0.119392 | -2.37253 | 0.017667 | 0.223834 |
| RP11-809C18.3 | -0.324015691 | 0.136956 | -2.36584 | 0.017989 | 0.223834 |
| RP11-820L6.1 | -0.27473877 | 0.116572 | -2.35682 | 0.018432 | 0.223834 |
| RP11-88H9.2 | 0.24135903 | 0.101668 | 2.373992 | 0.017597 | 0.223834 |
| RP5-894A10.2 | -0.223586314 | 0.094766 | -2.35935 | 0.018307 | 0.223834 |
| RP11-48B3.4 | -0.238724987 | 0.101716 | -2.34697 | 0.018927 | 0.228281 |
| RP5-894A10.6 | -0.239452234 | 0.102088 | -2.34556 | 0.018999 | 0.228281 |
| U91319.1 | 0.230566198 | 0.098417 | 2.342752 | 0.019142 | 0.228813 |
| AC009487.5 | 0.248723259 | 0.106327 | 2.339235 | 0.019323 | 0.229787 |
| LINC00174 | -0.244431189 | 0.104658 | -2.33552 | 0.019516 | 0.230891 |
| RP11-242D8.1 | -0.191014883 | 0.082001 | -2.32942 | 0.019837 | 0.233489 |
| ATP1A1OS | -0.281940235 | 0.121698 | -2.31672 | 0.020519 | 0.239073 |
| RP11-286B14.1 | 0.266249902 | 0.115113 | 2.312941 | 0.020726 | 0.239073 |
| RP11-861E21.2 | -0.243955081 | 0.105341 | -2.31585 | 0.020566 | 0.239073 |
| RP4-669L17.10 | -0.176272937 | 0.076197 | -2.31339 | 0.020701 | 0.239073 |
| RFPL1S | 0.215451481 | 0.093391 | 2.306979 | 0.021056 | 0.240476 |
| RP4-813F11.4 | -0.169298891 | 0.073345 | -2.30824 | 0.020986 | 0.240476 |
| TP53TG1 | -0.209830742 | 0.091129 | -2.30258 | 0.021303 | 0.242094 |
| AC007566.10 | -0.230510272 | 0.100299 | -2.29823 | 0.021549 | 0.242427 |
| AP003039.3 | -0.315054796 | 0.137104 | -2.29793 | 0.021566 | 0.242427 |
| RP11-513I15.6 | 0.171503955 | 0.07468 | 2.296503 | 0.021647 | 0.242427 |
| RP11-430B1.2 | 0.209422846 | 0.091322 | 2.293232 | 0.021835 | 0.243345 |
| MIR181A1HG | -0.258007328 | 0.112752 | -2.28827 | 0.022122 | 0.244185 |
| RP4-565E6.1 | 0.159139938 | 0.069519 | 2.289173 | 0.022069 | 0.244185 |
| RP11-400F19.18 | 0.171877426 | 0.075238 | 2.284448 | 0.022345 | 0.245478 |
| RP11-129K12.3 | -0.23055659 | 0.101234 | -2.27747 | 0.022758 | 0.246493 |
| RP11-380L11.3 | 0.175166359 | 0.076876 | 2.278548 | 0.022694 | 0.246493 |
| RP11-85M11.2 | 0.207509483 | 0.091029 | 2.279587 | 0.022632 | 0.246493 |
| RP5-1112D6.8 | -0.20894867 | 0.091949 | -2.27244 | 0.02306 | 0.248597 |
| AC007163.3 | -0.304895213 | 0.134706 | -2.26341 | 0.02361 | 0.248634 |
| ANKRD62P1-PARP4P3 | 0.28223488 | 0.124784 | 2.261792 | 0.02371 | 0.248634 |
| CTB-129O4.1 | 0.201144498 | 0.088845 | 2.263983 | 0.023575 | 0.248634 |
| RP11-1055B8.3 | -0.310587592 | 0.137155 | -2.2645 | 0.023543 | 0.248634 |
| RP11-45P15.4 | -0.221692489 | 0.097917 | -2.26409 | 0.023569 | 0.248634 |
| RP5-1073O3.7 | -0.21245476 | 0.093647 | -2.26867 | 0.023289 | 0.248634 |
| RP11-433J8.1 | 0.253785572 | 0.11242 | 2.257473 | 0.023979 | 0.25031 |
| RP1-178F15.5 | -0.247819631 | 0.110221 | -2.24839 | 0.024551 | 0.252855 |
| RP11-522I20.3 | -0.220052076 | 0.097798 | -2.25008 | 0.024444 | 0.252855 |
| ZNF518A | -0.137817767 | 0.061285 | -2.24881 | 0.024524 | 0.252855 |
| BMS1P20 | 0.147082152 | 0.065523 | 2.244756 | 0.024784 | 0.25319 |
| ENTPD3-AS1 | -0.233795027 | 0.104166 | -2.24445 | 0.024803 | 0.25319 |
| CTC-425O23.2 | -0.170233323 | 0.076069 | -2.23787 | 0.025229 | 0.254165 |
| PCBP1-AS1 | -0.119991899 | 0.053568 | -2.23998 | 0.025092 | 0.254165 |
| RP11-72I8.1 | 0.238289129 | 0.106452 | 2.238476 | 0.02519 | 0.254165 |
| AC018647.3 | -0.305329105 | 0.137064 | -2.22763 | 0.025905 | 0.257002 |
| FGD5-AS1 | -0.138314802 | 0.062115 | -2.22677 | 0.025963 | 0.257002 |
| LINC00937 | 0.165650822 | 0.074443 | 2.2252 | 0.026068 | 0.257002 |
| RP11-158M2.5 | -0.22979972 | 0.102981 | -2.23148 | 0.02565 | 0.257002 |
| RP11-288G11.3 | -0.300205474 | 0.13473 | -2.2282 | 0.025867 | 0.257002 |
| RP3-402G11.26 | 0.225131535 | 0.101393 | 2.220379 | 0.026393 | 0.259101 |
| AC083843.1 | -0.175286865 | 0.079128 | -2.21522 | 0.026745 | 0.259245 |
| RAD51-AS1 | -0.181689917 | 0.081973 | -2.21647 | 0.02666 | 0.259245 |
| RP11-624M8.1 | -0.277012478 | 0.125045 | -2.21531 | 0.026739 | 0.259245 |
| JPX | -0.1730633 | 0.078259 | -2.21142 | 0.027007 | 0.260688 |
| RP11-66D17.5 | 0.238774807 | 0.10812 | 2.208423 | 0.027215 | 0.261602 |
| LL0XNC01-116E7.2 | 0.196459085 | 0.089177 | 2.20303 | 0.027593 | 0.264133 |
| CTD-2020K17.3 | 0.220614468 | 0.100278 | 2.200027 | 0.027805 | 0.264863 |
| RP11-565F19.2 | -0.178249767 | 0.08107 | -2.19871 | 0.027898 | 0.264863 |
| RP11-589P10.7 | -0.210258257 | 0.095845 | -2.19374 | 0.028254 | 0.264967 |
| RP11-94L15.2 | -0.218608617 | 0.099602 | -2.19481 | 0.028177 | 0.264967 |
| UGDH-AS1 | 0.103603829 | 0.047202 | 2.194887 | 0.028172 | 0.264967 |
| CTB-131B5.2 | -0.224358145 | 0.102363 | -2.19178 | 0.028395 | 0.265215 |
| A1BG-AS1 | 0.168160458 | 0.07707 | 2.181908 | 0.029116 | 0.267613 |
| AC013268.5 | 0.264511389 | 0.121354 | 2.179663 | 0.029282 | 0.267613 |
| RP11-182L21.5 | -0.209141633 | 0.095839 | -2.18221 | 0.029094 | 0.267613 |
| RP11-345P4.9 | 0.141276849 | 0.064738 | 2.182276 | 0.029089 | 0.267613 |
| RP11-504P24.8 | -0.174796644 | 0.080201 | -2.17949 | 0.029295 | 0.267613 |
| RP11-522N14.2 | 0.205585451 | 0.094358 | 2.178778 | 0.029348 | 0.267613 |
| AC009492.1 | 0.25358177 | 0.116755 | 2.17192 | 0.029862 | 0.268058 |
| RP11-250B2.3 | -0.204622647 | 0.094134 | -2.17375 | 0.029724 | 0.268058 |
| RP11-391L3.1 | -0.153737411 | 0.07076 | -2.17267 | 0.029805 | 0.268058 |
| RP4-756G23.5 | -0.271273263 | 0.124877 | -2.17232 | 0.029832 | 0.268058 |
| RP11-1020A11.2 | -0.24560137 | 0.113264 | -2.16839 | 0.030129 | 0.26941 |
| RP11-122G18.5 | -0.13287951 | 0.061448 | -2.16246 | 0.030583 | 0.271808 |
| RP11-390E23.6 | -0.171056751 | 0.079127 | -2.16181 | 0.030633 | 0.271808 |
| CTD-2528L19.6 | -0.13165396 | 0.061253 | -2.14935 | 0.031606 | 0.273185 |
| LINC00639 | -0.29401705 | 0.136822 | -2.1489 | 0.031643 | 0.273185 |
| RP1-122P22.2 | -0.239726672 | 0.111134 | -2.1571 | 0.030998 | 0.273185 |
| RP11-299H21.1 | 0.232757283 | 0.108233 | 2.15053 | 0.031513 | 0.273185 |
| RP11-423H2.3 | 0.209931751 | 0.097746 | 2.147727 | 0.031735 | 0.273185 |
| RP11-464F9.1 | -0.163184478 | 0.075975 | -2.14787 | 0.031724 | 0.273185 |
| RP11-67A1.2 | -0.183867384 | 0.085416 | -2.15262 | 0.031349 | 0.273185 |
| RP11-701B16.2 | 0.167101119 | 0.077783 | 2.14831 | 0.031689 | 0.273185 |
| LINC00263 | -0.238832637 | 0.111461 | -2.14275 | 0.032133 | 0.27558 |
| AC007464.1 | -0.218973145 | 0.103322 | -2.11932 | 0.034063 | 0.280765 |
| AC015849.13 | -0.196360931 | 0.093386 | -2.10269 | 0.035493 | 0.280765 |
| ACAP2-IT1 | -0.163135375 | 0.077238 | -2.1121 | 0.034678 | 0.280765 |
| AF127936.5 | -0.278500379 | 0.132831 | -2.09666 | 0.036024 | 0.280765 |
| CTC-479C5.17 | -0.234680223 | 0.111962 | -2.09607 | 0.036076 | 0.280765 |
| CTD-2049O4.1 | -0.278702053 | 0.131563 | -2.11839 | 0.034142 | 0.280765 |
| CTD-2619J13.17 | -0.157370673 | 0.07487 | -2.10192 | 0.03556 | 0.280765 |
| CTD-3099C6.9 | -0.217468199 | 0.102531 | -2.121 | 0.033922 | 0.280765 |
| MKLN1-AS2 | -0.155138905 | 0.073531 | -2.10985 | 0.034871 | 0.280765 |
| OR2A1-AS1 | -0.192327142 | 0.09091 | -2.11559 | 0.03438 | 0.280765 |
| RP11-13K12.1 | 0.235817788 | 0.112154 | 2.102622 | 0.035499 | 0.280765 |
| RP11-20I23.8 | 0.195638121 | 0.092974 | 2.104224 | 0.035359 | 0.280765 |
| RP11-267A15.1 | -0.284813324 | 0.133939 | -2.12644 | 0.033467 | 0.280765 |
| RP11-400K9.4 | -0.27788317 | 0.132623 | -2.09529 | 0.036145 | 0.280765 |
| RP11-416I2.1 | 0.228882684 | 0.107802 | 2.123185 | 0.033738 | 0.280765 |
| RP11-44N21.1 | 0.210926894 | 0.10063 | 2.096067 | 0.036076 | 0.280765 |
| RP11-482M8.3 | 0.227244494 | 0.107265 | 2.118524 | 0.034131 | 0.280765 |
| RP11-552E20.4 | 0.236069305 | 0.111934 | 2.109001 | 0.034944 | 0.280765 |
| RP11-571M6.8 | 0.195794839 | 0.091822 | 2.132335 | 0.032979 | 0.280765 |
| RP11-588K22.2 | -0.149067828 | 0.070958 | -2.10079 | 0.035659 | 0.280765 |
| RP11-61A14.3 | -0.144951958 | 0.068948 | -2.10232 | 0.035525 | 0.280765 |
| RP11-61L19.1 | -0.206755071 | 0.097515 | -2.12024 | 0.033986 | 0.280765 |
| RP11-932O9.9 | -0.178770403 | 0.084794 | -2.1083 | 0.035005 | 0.280765 |
| RP11-95O2.1 | 0.212120721 | 0.101014 | 2.09991 | 0.035737 | 0.280765 |
| RP4-753M9.1 | -0.247515291 | 0.116871 | -2.11785 | 0.034188 | 0.280765 |
| RP4-773A18.4 | 0.197006809 | 0.093737 | 2.101704 | 0.035579 | 0.280765 |
| RP5-1052I5.1 | 0.236421778 | 0.112416 | 2.10309 | 0.035458 | 0.280765 |
| THAP9-AS1 | 0.154230825 | 0.072849 | 2.117132 | 0.034249 | 0.280765 |
| RP11-75C9.1 | -0.233649319 | 0.111608 | -2.09348 | 0.036306 | 0.281069 |
| RP11-586K2.1 | 0.21686131 | 0.103692 | 2.091397 | 0.036493 | 0.281566 |
| RP11-863P13.4 | 0.24206332 | 0.115986 | 2.087007 | 0.036887 | 0.283665 |
| AC137723.5 | 0.222666909 | 0.107242 | 2.076305 | 0.037866 | 0.289259 |
| RP11-143K11.1 | 0.240263832 | 0.115649 | 2.077532 | 0.037752 | 0.289259 |
| ANKRD10-IT1 | -0.170820907 | 0.08255 | -2.06931 | 0.038517 | 0.293261 |
| TPTEP1 | -0.243583532 | 0.117825 | -2.06734 | 0.038702 | 0.293702 |
| CTC-524C5.2 | 0.233104136 | 0.113067 | 2.06164 | 0.039242 | 0.293933 |
| FAM13A-AS1 | -0.149821255 | 0.072602 | -2.06361 | 0.039055 | 0.293933 |
| RP11-397O8.7 | -0.201548142 | 0.097732 | -2.06225 | 0.039184 | 0.293933 |
| RP4-555D20.2 | 0.214951142 | 0.104087 | 2.065103 | 0.038913 | 0.293933 |
| AC062029.1 | -0.204785394 | 0.099722 | -2.05357 | 0.040017 | 0.29452 |
| FAM157C | -0.215094541 | 0.104735 | -2.0537 | 0.040005 | 0.29452 |
| RP11-333E1.1 | 0.144053508 | 0.070125 | 2.054235 | 0.039953 | 0.29452 |
| RP11-554A11.9 | 0.239170693 | 0.116506 | 2.052858 | 0.040086 | 0.29452 |
| RP11-689P11.2 | 0.184886424 | 0.089986 | 2.054614 | 0.039916 | 0.29452 |
| RP11-71N10.1 | -0.279560079 | 0.136116 | -2.05384 | 0.039991 | 0.29452 |
| RP1-239B22.5 | 0.158239231 | 0.077234 | 2.048823 | 0.040479 | 0.296464 |
| LIPE-AS1 | 0.120762746 | 0.058995 | 2.046984 | 0.04066 | 0.296841 |
| RP11-182J1.13 | 0.206008762 | 0.100707 | 2.045626 | 0.040793 | 0.296877 |
| PRKAG2-AS1 | 0.183913775 | 0.090138 | 2.040351 | 0.041315 | 0.29689 |
| RP11-115H15.2 | -0.208214131 | 0.102173 | -2.03786 | 0.041564 | 0.29689 |
| RP11-182L21.6 | -0.14513905 | 0.071093 | -2.04153 | 0.041199 | 0.29689 |
| RP11-259O2.3 | 0.116609545 | 0.057111 | 2.041803 | 0.041171 | 0.29689 |
| RP11-274H2.5 | -0.258128064 | 0.126544 | -2.03983 | 0.041367 | 0.29689 |
| RP11-705C15.3 | -0.179396894 | 0.08805 | -2.03744 | 0.041606 | 0.29689 |
| TTTY15 | -0.158956768 | 0.078052 | -2.03654 | 0.041696 | 0.29689 |
| CTA-217C2.1 | 0.147495354 | 0.072525 | 2.033729 | 0.041979 | 0.297986 |
| AC105053.4 | -0.278244061 | 0.137143 | -2.02886 | 0.042472 | 0.299014 |
| RP11-350F4.2 | -0.156526211 | 0.077148 | -2.0289 | 0.042469 | 0.299014 |
| RUSC1-AS1 | -0.233903817 | 0.115311 | -2.02847 | 0.042513 | 0.299014 |
| LINC00086 | 0.17868434 | 0.088239 | 2.025014 | 0.042866 | 0.300583 |
| RP11-503E24.2 | -0.190059099 | 0.093948 | -2.02301 | 0.043072 | 0.301111 |
| AC025171.1 | -0.266693102 | 0.13217 | -2.0178 | 0.043612 | 0.3014 |
| AC092620.2 | -0.137818657 | 0.068309 | -2.01757 | 0.043636 | 0.3014 |
| RP11-73M18.8 | -0.192606877 | 0.095367 | -2.01964 | 0.043421 | 0.3014 |
| TTC25 | 0.159253663 | 0.078796 | 2.021099 | 0.04327 | 0.3014 |
| RP11-521O16.2 | 0.213820102 | 0.106224 | 2.012926 | 0.044122 | 0.303852 |
| RP11-344N10.5 | -0.164504556 | 0.081916 | -2.00821 | 0.044621 | 0.306368 |
| CTC-525D6.2 | 0.210457814 | 0.105158 | 2.001345 | 0.045355 | 0.306813 |
| CTD-2517M14.5 | 0.212916082 | 0.106338 | 2.002254 | 0.045257 | 0.306813 |
| CTD-2562J17.4 | 0.189501795 | 0.094525 | 2.004779 | 0.044987 | 0.306813 |
| IQCH-AS1 | -0.165948595 | 0.083121 | -1.99647 | 0.045882 | 0.306813 |
| RP11-281O15.4 | 0.194264821 | 0.097282 | 1.99693 | 0.045833 | 0.306813 |
| RP11-32K4.1 | 0.204558653 | 0.102316 | 1.999276 | 0.045578 | 0.306813 |
| RP11-539G18.2 | -0.225780011 | 0.113034 | -1.99745 | 0.045776 | 0.306813 |
| RP11-98D18.3 | 0.202718328 | 0.101364 | 1.999904 | 0.045511 | 0.306813 |
| SRD5A3-AS1 | -0.210953493 | 0.105459 | -2.00034 | 0.045464 | 0.306813 |
| RP11-58B17.2 | 0.166212651 | 0.083356 | 1.994005 | 0.046152 | 0.307721 |
| RP11-66N24.4 | 0.119879064 | 0.060183 | 1.991897 | 0.046382 | 0.308369 |
| CTA-204B4.2 | -0.198120563 | 0.099592 | -1.98933 | 0.046665 | 0.308472 |
| ZFHX4-AS1 | -0.238807182 | 0.120003 | -1.99 | 0.04659 | 0.308472 |
| RP6-109B7.3 | -0.185837054 | 0.093504 | -1.98749 | 0.046868 | 0.30893 |
| MIR24-2 | -0.243902196 | 0.122865 | -1.98512 | 0.047131 | 0.309775 |
| AC108488.3 | -0.221041529 | 0.111916 | -1.97506 | 0.048262 | 0.310827 |
| CTD-2555O16.4 | -0.159336373 | 0.080604 | -1.97677 | 0.048067 | 0.310827 |
| RP11-10J21.4 | 0.217404825 | 0.109855 | 1.979013 | 0.047815 | 0.310827 |
| RP11-178G16.4 | -0.177327796 | 0.089827 | -1.97411 | 0.048369 | 0.310827 |
| RP11-482M8.1 | 0.174222438 | 0.088108 | 1.977371 | 0.048 | 0.310827 |
| RP11-4O1.2 | 0.17734251 | 0.089773 | 1.975449 | 0.048217 | 0.310827 |
| RP11-792A8.4 | -0.176416526 | 0.089309 | -1.97536 | 0.048228 | 0.310827 |
| RP6-65G23.3 | 0.164188189 | 0.083007 | 1.977993 | 0.047929 | 0.310827 |
| RP11-177H13.2 | 0.2168341 | 0.110022 | 1.970826 | 0.048744 | 0.312366 |
| SLC38A3 | -0.201293174 | 0.102256 | -1.96851 | 0.049009 | 0.313196 |
| RP11-190A12.8 | 0.248383384 | 0.126561 | 1.962558 | 0.049698 | 0.315406 |
| TERC | 0.159825685 | 0.081383 | 1.963883 | 0.049544 | 0.315406 |
| WAC-AS1 | 0.12721877 | 0.064842 | 1.961978 | 0.049765 | 0.315406 |
| LINC00601 | -0.264694467 | 0.135266 | -1.95684 | 0.050366 | 0.318339 |
| ZEB1-AS1 | -0.145152286 | 0.074323 | -1.95298 | 0.050822 | 0.320342 |
| RP1-146A15.1 | -0.225960957 | 0.116054 | -1.94704 | 0.05153 | 0.323922 |
| SIK3-IT1 | -0.214027163 | 0.110072 | -1.94444 | 0.051843 | 0.325004 |
| DDX11-AS1 | 0.176158995 | 0.090719 | 1.941814 | 0.05216 | 0.326104 |
| LINC00478 | -0.12046992 | 0.062154 | -1.93826 | 0.052591 | 0.327029 |
| RP11-303E16.8 | -0.172095771 | 0.088737 | -1.9394 | 0.052452 | 0.327029 |
| RP11-44N11.1 | -0.217629887 | 0.112367 | -1.93678 | 0.052773 | 0.327277 |
| RP11-96H17.1 | 0.234763053 | 0.121386 | 1.934021 | 0.053111 | 0.328488 |
| LINC00320 | -0.253145832 | 0.131239 | -1.9289 | 0.053744 | 0.328883 |
| MIR4435-1HG | 0.188307847 | 0.097584 | 1.929708 | 0.053643 | 0.328883 |
| RP11-436K8.1 | -0.235442012 | 0.122061 | -1.92889 | 0.053745 | 0.328883 |
| SNHG5 | -0.166462345 | 0.086256 | -1.92987 | 0.053623 | 0.328883 |
| RP11-285F7.2 | -0.26403452 | 0.137167 | -1.92492 | 0.05424 | 0.330929 |
| TMEM51-AS1 | -0.191221218 | 0.099392 | -1.92391 | 0.054366 | 0.330929 |
| LINC00032 | -0.223851216 | 0.116507 | -1.92135 | 0.054688 | 0.331261 |
| RAB30-AS1 | -0.126120712 | 0.065649 | -1.92113 | 0.054715 | 0.331261 |
| RP11-401P9.4 | -0.198399278 | 0.10333 | -1.92005 | 0.054851 | 0.331261 |
| RP11-283I3.6 | -0.128085785 | 0.066752 | -1.91884 | 0.055005 | 0.331324 |
| MIR4453 | -0.116566236 | 0.06101 | -1.9106 | 0.056056 | 0.331605 |
| RAMP2-AS1 | -0.154264288 | 0.080595 | -1.91407 | 0.055611 | 0.331605 |
| RP11-17L5.4 | 0.169199018 | 0.088385 | 1.914348 | 0.055576 | 0.331605 |
| RP11-383J24.6 | -0.118321285 | 0.061929 | -1.91058 | 0.056058 | 0.331605 |
| RP11-627G23.1 | 0.216941034 | 0.113486 | 1.911615 | 0.055926 | 0.331605 |
| RP11-64K12.10 | 0.204310811 | 0.106773 | 1.913506 | 0.055683 | 0.331605 |
| RP5-890E16.2 | -0.195679577 | 0.102073 | -1.91706 | 0.05523 | 0.331605 |
| HLA-F-AS1 | -0.191066472 | 0.100073 | -1.90928 | 0.056226 | 0.33175 |
| TRHDE-AS1 | 0.173686604 | 0.091025 | 1.908119 | 0.056376 | 0.331784 |
| DLGAP1-AS2 | -0.225719379 | 0.118396 | -1.90647 | 0.056589 | 0.332189 |
| INTS6-AS1 | -0.140861085 | 0.073985 | -1.9039 | 0.056923 | 0.333302 |
| NPSR1-AS1 | 0.253817808 | 0.133765 | 1.897485 | 0.057764 | 0.337371 |
| CTD-2349P21.5 | 0.158697831 | 0.083755 | 1.894775 | 0.058122 | 0.338467 |
| RP11-798L4.1 | -0.184671421 | 0.097511 | -1.89385 | 0.058245 | 0.338467 |
| C20orf166-AS1 | 0.244228629 | 0.129043 | 1.892614 | 0.058409 | 0.338568 |
| C6orf3 | -0.185207707 | 0.098461 | -1.88103 | 0.059968 | 0.344158 |
| LINC00338 | 0.104400494 | 0.055502 | 1.881011 | 0.05997 | 0.344158 |
| RP11-123O10.3 | -0.198075554 | 0.105225 | -1.8824 | 0.059781 | 0.344158 |
| RP11-799B12.4 | -0.137364667 | 0.072998 | -1.88175 | 0.05987 | 0.344158 |
| LINC00939 | -0.212483466 | 0.113287 | -1.87563 | 0.060707 | 0.347519 |
| RP11-13K12.2 | 0.173614292 | 0.092766 | 1.871522 | 0.061273 | 0.349028 |
| RP11-981G7.6 | 0.145997595 | 0.078009 | 1.871553 | 0.061269 | 0.349028 |
| RP11-588H23.3 | -0.223215828 | 0.119429 | -1.86902 | 0.06162 | 0.350141 |
| CTC-559E9.5 | -0.129326572 | 0.069296 | -1.86629 | 0.062001 | 0.35058 |
| RP11-105N13.4 | 0.118110836 | 0.06327 | 1.866771 | 0.061934 | 0.35058 |
| AC008269.2 | 0.200652435 | 0.107885 | 1.859881 | 0.062902 | 0.351161 |
| ARHGAP5-AS1 | -0.179026893 | 0.096512 | -1.85497 | 0.063601 | 0.351161 |
| BDNF-AS | -0.179530716 | 0.096828 | -1.85413 | 0.063721 | 0.351161 |
| CTD-3025N20.2 | -0.247573004 | 0.133208 | -1.85855 | 0.063091 | 0.351161 |
| LINC00574 | 0.186733356 | 0.100519 | 1.857699 | 0.063212 | 0.351161 |
| LINC00886 | -0.204659291 | 0.110058 | -1.85955 | 0.062949 | 0.351161 |
| RP11-254F7.2 | 0.16737277 | 0.089939 | 1.860957 | 0.06275 | 0.351161 |
| RP11-262H14.4 | -0.162717619 | 0.087695 | -1.85549 | 0.063527 | 0.351161 |
| RP11-395P17.3 | -0.148901006 | 0.080118 | -1.85852 | 0.063095 | 0.351161 |
| RP4-535B20.4 | 0.180241503 | 0.097123 | 1.855812 | 0.06348 | 0.351161 |
| U62631.5 | -0.246287839 | 0.132861 | -1.85373 | 0.063778 | 0.351161 |
| LINC00884 | 0.163840645 | 0.088462 | 1.852111 | 0.06401 | 0.351597 |
| RP11-204M4.2 | 0.192256797 | 0.103957 | 1.849393 | 0.064401 | 0.352906 |
| AC000120.7 | -0.113340492 | 0.061484 | -1.84341 | 0.065269 | 0.352958 |
| AC016995.3 | 0.19339402 | 0.104772 | 1.845848 | 0.064914 | 0.352958 |
| BAIAP2-AS1 | 0.162858949 | 0.088511 | 1.839984 | 0.065771 | 0.352958 |
| CASC7 | -0.13050706 | 0.070681 | -1.84643 | 0.064829 | 0.352958 |
| CTD-2542L18.1 | 0.198465325 | 0.107925 | 1.838927 | 0.065926 | 0.352958 |
| FOXP1-IT1 | -0.145925225 | 0.079209 | -1.84229 | 0.065433 | 0.352958 |
| IDI2-AS1 | -0.190116917 | 0.10339 | -1.83883 | 0.065941 | 0.352958 |
| LINC00152 | 0.192208477 | 0.104246 | 1.843798 | 0.065213 | 0.352958 |
| RP1-272J12.1 | 0.197533586 | 0.106978 | 1.846496 | 0.06482 | 0.352958 |
| RP11-466A19.1 | 0.232083551 | 0.126093 | 1.84058 | 0.065683 | 0.352958 |
| CTA-254O6.1 | 0.199958681 | 0.108848 | 1.837052 | 0.066202 | 0.353539 |
| USP3-AS1 | 0.14646019 | 0.080137 | 1.827615 | 0.067607 | 0.360208 |
| RP11-332H14.2 | -0.140688948 | 0.077065 | -1.82559 | 0.067911 | 0.360994 |
| CTD-2292P10.4 | 0.164688633 | 0.090378 | 1.822225 | 0.068421 | 0.361289 |
| LINC00957 | 0.136228368 | 0.074848 | 1.82006 | 0.06875 | 0.361289 |
| MATN1-AS1 | -0.237530271 | 0.130499 | -1.82018 | 0.068732 | 0.361289 |
| RP3-395M20.12 | 0.221283272 | 0.121442 | 1.822135 | 0.068435 | 0.361289 |
| SBF2-AS1 | 0.163975702 | 0.090019 | 1.821565 | 0.068521 | 0.361289 |
| CTD-2587H19.2 | -0.15726942 | 0.086823 | -1.81137 | 0.070084 | 0.365798 |
| CTD-2620I22.1 | 0.201996038 | 0.111488 | 1.811818 | 0.070014 | 0.365798 |
| LINC00092 | -0.176436287 | 0.097404 | -1.81139 | 0.07008 | 0.365798 |
| ZNF674-AS1 | -0.13946195 | 0.077208 | -1.80633 | 0.070867 | 0.369054 |
| LINC01001 | -0.192135133 | 0.106892 | -1.79747 | 0.072261 | 0.371281 |
| RP11-166O4.6 | -0.14291813 | 0.079508 | -1.79754 | 0.07225 | 0.371281 |
| RP11-260M2.1 | -0.15481684 | 0.08612 | -1.79769 | 0.072227 | 0.371281 |
| RP11-66B24.4 | 0.211779728 | 0.117567 | 1.801356 | 0.071647 | 0.371281 |
| RP11-73M18.7 | -0.188757644 | 0.10478 | -1.80146 | 0.07163 | 0.371281 |
| RP4-802A10.1 | -0.215121323 | 0.119547 | -1.79947 | 0.071944 | 0.371281 |
| MIR29B1 | 0.180691761 | 0.100617 | 1.795836 | 0.072521 | 0.371789 |
| ASB16-AS1 | -0.133065719 | 0.074168 | -1.7941 | 0.072797 | 0.371872 |
| CTC-444N24.11 | -0.089098999 | 0.049871 | -1.78661 | 0.074001 | 0.371872 |
| H1FX-AS1 | 0.144037139 | 0.080682 | 1.785243 | 0.074222 | 0.371872 |
| MEG9 | 0.17580478 | 0.098512 | 1.784603 | 0.074326 | 0.371872 |
| PART1 | 0.199941307 | 0.11198 | 1.78551 | 0.074179 | 0.371872 |
| RP1-249H1.4 | -0.2343647 | 0.131351 | -1.78427 | 0.07438 | 0.371872 |
| RP11-1109F11.3 | -0.181427673 | 0.101692 | -1.78409 | 0.074408 | 0.371872 |
| RP11-120M18.2 | 0.129098087 | 0.072416 | 1.782717 | 0.074632 | 0.371872 |
| RP11-31F19.1 | -0.222490988 | 0.12439 | -1.78866 | 0.07367 | 0.371872 |
| RP11-337C18.9 | -0.167712841 | 0.093912 | -1.78584 | 0.074125 | 0.371872 |
| RP11-509J21.1 | -0.195129334 | 0.109427 | -1.78319 | 0.074555 | 0.371872 |
| RP11-53I6.2 | -0.195057225 | 0.109175 | -1.78665 | 0.073995 | 0.371872 |
| RP11-712B9.2 | -0.157850948 | 0.088027 | -1.79321 | 0.07294 | 0.371872 |
| CHL1-AS2 | 0.182672118 | 0.102588 | 1.780633 | 0.074972 | 0.372262 |
| RP11-145P16.3 | -0.168698419 | 0.094764 | -1.7802 | 0.075043 | 0.372262 |
| RP11-277L2.3 | 0.188844415 | 0.106136 | 1.779275 | 0.075195 | 0.372262 |
| MIR137HG | 0.169256401 | 0.095443 | 1.773382 | 0.076165 | 0.375497 |
| RP11-384F7.2 | 0.104414109 | 0.05888 | 1.773332 | 0.076174 | 0.375497 |
| AC137934.1 | -0.134047585 | 0.075709 | -1.77056 | 0.076634 | 0.376061 |
| RP11-359E10.1 | 0.166108131 | 0.093906 | 1.768874 | 0.076915 | 0.376061 |
| RP11-85K15.2 | -0.151036918 | 0.085393 | -1.76872 | 0.07694 | 0.376061 |
| USP2-AS1 | 0.190497277 | 0.107672 | 1.769235 | 0.076855 | 0.376061 |
| LINC00594 | 0.193148721 | 0.109305 | 1.767062 | 0.077218 | 0.37621 |
| RP11-33B1.3 | -0.111614987 | 0.063203 | -1.76599 | 0.077398 | 0.37621 |
| RPS10P7 | -0.176575445 | 0.100008 | -1.76562 | 0.07746 | 0.37621 |
| AP000253.1 | -0.157432673 | 0.089807 | -1.75301 | 0.0796 | 0.377078 |
| AP000692.9 | -0.161647832 | 0.092059 | -1.75592 | 0.079102 | 0.377078 |
| ARHGEF26-AS1 | -0.157385971 | 0.089714 | -1.7543 | 0.079379 | 0.377078 |
| CTC-428G20.3 | -0.144955728 | 0.082366 | -1.7599 | 0.078425 | 0.377078 |
| LINC00202-1 | 0.195357729 | 0.111154 | 1.757547 | 0.078825 | 0.377078 |
| LINC00662 | 0.077897546 | 0.044305 | 1.758223 | 0.07871 | 0.377078 |
| RP11-215G15.5 | 0.181191089 | 0.103237 | 1.755091 | 0.079244 | 0.377078 |
| RP11-258C19.5 | 0.093388965 | 0.053233 | 1.754356 | 0.07937 | 0.377078 |
| RP11-373N22.3 | -0.165500988 | 0.094025 | -1.76017 | 0.078378 | 0.377078 |
| RP11-517H2.6 | 0.078131995 | 0.044486 | 1.756325 | 0.079033 | 0.377078 |
| RP13-895J2.7 | 0.199148686 | 0.113018 | 1.762101 | 0.078052 | 0.377078 |
| SLC26A4-AS1 | 0.167821021 | 0.095716 | 1.753326 | 0.079546 | 0.377078 |
| CTD-3162L10.1 | 0.218586464 | 0.124955 | 1.74932 | 0.080236 | 0.378856 |
| RP11-197K6.1 | 0.207371453 | 0.118705 | 1.746949 | 0.080646 | 0.378856 |
| RP11-304F15.3 | 0.175852916 | 0.100622 | 1.747653 | 0.080524 | 0.378856 |
| RP11-73M18.6 | -0.168874813 | 0.096725 | -1.74592 | 0.080824 | 0.378856 |
| TTLL7-IT1 | -0.20045263 | 0.114674 | -1.74802 | 0.08046 | 0.378856 |
| ZBED3-AS1 | -0.152056848 | 0.087132 | -1.74514 | 0.080961 | 0.378856 |
| AP000473.5 | -0.159777939 | 0.091762 | -1.74122 | 0.081645 | 0.379201 |
| GPC5-IT1 | -0.221637614 | 0.127416 | -1.73948 | 0.08195 | 0.379201 |
| MAP3K14-AS1 | 0.175324803 | 0.10081 | 1.739167 | 0.082005 | 0.379201 |
| RP11-498C9.15 | -0.136427153 | 0.078278 | -1.74286 | 0.081359 | 0.379201 |
| RP4-782G3.1 | 0.18011259 | 0.103483 | 1.740496 | 0.081772 | 0.379201 |
| TRIL | -0.194872441 | 0.112055 | -1.73908 | 0.082021 | 0.379201 |
| RP11-158M2.3 | -0.175349311 | 0.100907 | -1.73773 | 0.082258 | 0.379539 |
| LINC00877 | -0.229183564 | 0.132103 | -1.73488 | 0.082761 | 0.381098 |
| F11-AS1 | -0.194031617 | 0.112111 | -1.73071 | 0.083504 | 0.381521 |
| RP11-617D20.1 | -0.122975261 | 0.070968 | -1.73284 | 0.083125 | 0.381521 |
| RP11-769O8.3 | -0.185940278 | 0.10744 | -1.73065 | 0.083515 | 0.381521 |
| RP11-989F5.3 | 0.184716718 | 0.106724 | 1.730788 | 0.08349 | 0.381521 |
| AP000439.1 | -0.232897871 | 0.134686 | -1.72919 | 0.083774 | 0.381951 |
| RP11-380L11.4 | 0.181130185 | 0.104864 | 1.72729 | 0.084116 | 0.382751 |
| CTC-444N24.8 | -0.161571754 | 0.093753 | -1.72338 | 0.084819 | 0.384437 |
| ZNF33B | 0.116023891 | 0.067316 | 1.723573 | 0.084785 | 0.384437 |
| CYP4F35P | 0.183687093 | 0.106674 | 1.721945 | 0.085079 | 0.38486 |
| AC025335.1 | 0.102356408 | 0.059617 | 1.716899 | 0.085998 | 0.386122 |
| RP11-483C6.1 | -0.151697445 | 0.088364 | -1.71673 | 0.086028 | 0.386122 |
| RP11-798G7.8 | -0.213033118 | 0.124031 | -1.71758 | 0.085874 | 0.386122 |
| TRAM2-AS1 | -0.155625337 | 0.090601 | -1.7177 | 0.085851 | 0.386122 |
| CTC-529P8.1 | -0.228291618 | 0.133054 | -1.71578 | 0.086202 | 0.386153 |
| RP11-248G5.8 | -0.163412785 | 0.095379 | -1.7133 | 0.086658 | 0.387442 |
| RP11-424G14.1 | -0.186282404 | 0.108791 | -1.71229 | 0.086843 | 0.387516 |
| CPB2-AS1 | -0.194820446 | 0.113869 | -1.71092 | 0.087097 | 0.387899 |
| RP11-981G7.2 | 0.135034254 | 0.078999 | 1.709323 | 0.087391 | 0.388461 |
| AC019118.3 | 0.197042397 | 0.115595 | 1.704586 | 0.088272 | 0.389374 |
| CTC-246B18.8 | 0.179347877 | 0.105182 | 1.705115 | 0.088173 | 0.389374 |
| ERICH1-AS1 | 0.188717698 | 0.110555 | 1.706997 | 0.087823 | 0.389374 |
| RP3-467N11.1 | 0.165290604 | 0.096966 | 1.70463 | 0.088263 | 0.389374 |
| CTD-2324F15.2 | -0.174588078 | 0.102515 | -1.70304 | 0.08856 | 0.389901 |
| AC018730.1 | -0.132191259 | 0.077717 | -1.70093 | 0.088956 | 0.390899 |
| PLK1S1 | -0.116715488 | 0.068732 | -1.69812 | 0.089484 | 0.391727 |
| RP11-490M8.1 | 0.153271311 | 0.090242 | 1.698447 | 0.089424 | 0.391727 |
| RP3-428L16.1 | 0.169523398 | 0.099913 | 1.696717 | 0.08975 | 0.392147 |
| WDFY3-AS2 | -0.178120384 | 0.105065 | -1.69534 | 0.090011 | 0.392545 |
| EMX2OS | -0.167042108 | 0.09872 | -1.69207 | 0.090632 | 0.39269 |
| RP11-448A19.1 | -0.151066786 | 0.089352 | -1.69069 | 0.090896 | 0.39269 |
| RP11-546J1.1 | 0.157073798 | 0.09284 | 1.69188 | 0.090669 | 0.39269 |
| RP11-703G6.1 | 0.115585561 | 0.068251 | 1.693526 | 0.090355 | 0.39269 |
| RP3-402G11.25 | 0.189054264 | 0.111804 | 1.690945 | 0.090847 | 0.39269 |
| RP11-26J3.3 | -0.159418132 | 0.094621 | -1.68481 | 0.092026 | 0.39683 |
| LINC00630 | 0.089187625 | 0.053057 | 1.68097 | 0.092769 | 0.397064 |
| PSMD6-AS2 | -0.138965907 | 0.082648 | -1.68142 | 0.092682 | 0.397064 |
| RP11-206L10.11 | 0.13005788 | 0.077307 | 1.682351 | 0.092501 | 0.397064 |
| RP11-440L14.1 | -0.124866506 | 0.074197 | -1.68289 | 0.092395 | 0.397064 |
| RP11-177G23.2 | -0.164136631 | 0.097703 | -1.67995 | 0.092968 | 0.397178 |
| RP5-1085F17.3 | -0.105943242 | 0.06312 | -1.67845 | 0.09326 | 0.397692 |
| RP11-517P14.2 | -0.192352733 | 0.114833 | -1.67507 | 0.09392 | 0.399767 |
| GS1-72M22.1 | 0.154353016 | 0.092371 | 1.671015 | 0.094719 | 0.400947 |
| RASSF8-AS1 | -0.118913608 | 0.071091 | -1.6727 | 0.094386 | 0.400947 |
| RP11-308D16.4 | -0.152707691 | 0.091367 | -1.67137 | 0.094649 | 0.400947 |
| AP006222.2 | 0.206663477 | 0.123811 | 1.669187 | 0.09508 | 0.401006 |
| RP11-110G21.1 | -0.141989149 | 0.085041 | -1.66966 | 0.094987 | 0.401006 |
| RP11-145M9.4 | -0.124622878 | 0.074931 | -1.66317 | 0.096278 | 0.40311 |
| RP11-498D10.6 | -0.160927971 | 0.096726 | -1.66374 | 0.096164 | 0.40311 |
| RP11-572C21.1 | 0.196183495 | 0.117821 | 1.665091 | 0.095895 | 0.40311 |
| XXbac-B461K10.4 | 0.089754794 | 0.053954 | 1.663556 | 0.096201 | 0.40311 |
| AC090587.4 | 0.150432804 | 0.090558 | 1.661176 | 0.096678 | 0.404052 |
| ZBTB11-AS1 | -0.119039129 | 0.071883 | -1.65601 | 0.09772 | 0.407668 |
| ALMS1-IT1 | -0.183640746 | 0.111052 | -1.65365 | 0.098199 | 0.408214 |
| RP11-549J18.1 | -0.146105068 | 0.088355 | -1.65362 | 0.098205 | 0.408214 |
| LINC00461 | -0.129411159 | 0.078344 | -1.65184 | 0.098568 | 0.408251 |
| RP11-420A23.1 | -0.17483622 | 0.105819 | -1.65223 | 0.098488 | 0.408251 |
| CTD-2293H3.1 | -0.152744638 | 0.092585 | -1.64978 | 0.098989 | 0.408498 |
| LINC00240 | -0.173650527 | 0.105349 | -1.64834 | 0.099283 | 0.408498 |
| RP11-384P7.7 | -0.13784457 | 0.083637 | -1.64812 | 0.099328 | 0.408498 |
| TINCR | 0.189526673 | 0.114998 | 1.648083 | 0.099336 | 0.408498 |
| AP001258.4 | -0.156373114 | 0.095269 | -1.64139 | 0.100717 | 0.412034 |
| MIR497HG | -0.163980247 | 0.099932 | -1.64093 | 0.100813 | 0.412034 |
| RP11-449P15.2 | -0.192868305 | 0.11757 | -1.64046 | 0.10091 | 0.412034 |
| RP5-836N10.1 | -0.166275044 | 0.101358 | -1.64047 | 0.100907 | 0.412034 |
| AC007551.3 | -0.174352305 | 0.106462 | -1.63769 | 0.101486 | 0.412196 |
| RP11-421E14.2 | -0.115635297 | 0.070593 | -1.63805 | 0.101411 | 0.412196 |
| RP4-562J12.2 | -0.159982723 | 0.097596 | -1.63924 | 0.101163 | 0.412196 |
| LINC00299 | -0.187315258 | 0.114589 | -1.63467 | 0.102118 | 0.412584 |
| RP11-259K15.2 | -0.21568875 | 0.131924 | -1.63495 | 0.102059 | 0.412584 |
| RP11-715J22.6 | -0.163038434 | 0.099655 | -1.63603 | 0.101833 | 0.412584 |
| RP11-137H2.6 | -0.136822559 | 0.083795 | -1.63282 | 0.102507 | 0.413429 |
| RP11-390P2.4 | -0.198451715 | 0.121602 | -1.63197 | 0.102685 | 0.413429 |
| RP11-10K16.1 | -0.14903036 | 0.091432 | -1.62997 | 0.103109 | 0.414411 |
| AC002116.8 | 0.13028497 | 0.080127 | 1.625989 | 0.103952 | 0.416948 |
| AC018766.5 | -0.177856 | 0.109488 | -1.62444 | 0.104282 | 0.416948 |
| LINC00847 | -0.118120911 | 0.072705 | -1.62466 | 0.104234 | 0.416948 |
| AC019221.4 | -0.146614664 | 0.090323 | -1.62322 | 0.104542 | 0.417014 |
| RP6-42F4.1 | -0.104398547 | 0.064338 | -1.62267 | 0.10466 | 0.417014 |
| RP11-867G2.8 | 0.160721854 | 0.099145 | 1.621081 | 0.105 | 0.417647 |
| AC009948.5 | 0.08957688 | 0.055428 | 1.616101 | 0.106072 | 0.419023 |
| RP11-17M24.1 | 0.181000342 | 0.111855 | 1.618175 | 0.105625 | 0.419023 |
| RP11-318M2.2 | -0.203972963 | 0.126085 | -1.61774 | 0.105718 | 0.419023 |
| TMEM191A | 0.166763743 | 0.103146 | 1.616775 | 0.105927 | 0.419023 |
| AC004540.4 | -0.201455398 | 0.124886 | -1.61312 | 0.106719 | 0.4199 |
| AC007092.1 | 0.115449979 | 0.071641 | 1.611497 | 0.107071 | 0.4199 |
| AC009014.3 | 0.219300251 | 0.136812 | 1.602932 | 0.10895 | 0.4199 |
| AC091878.1 | 0.139539169 | 0.086476 | 1.61362 | 0.10661 | 0.4199 |
| AP000688.29 | -0.209178535 | 0.130424 | -1.60384 | 0.10875 | 0.4199 |
| CTD-2516F10.2 | -0.184783262 | 0.115217 | -1.60379 | 0.108761 | 0.4199 |
| CTD-2541J13.1 | -0.169714546 | 0.10557 | -1.60759 | 0.107924 | 0.4199 |
| FAM83H-AS1 | 0.163768673 | 0.101682 | 1.610603 | 0.107266 | 0.4199 |
| RNF219-AS1 | -0.195880571 | 0.122211 | -1.6028 | 0.108978 | 0.4199 |
| RP11-14N7.2 | 0.115470572 | 0.072052 | 1.602592 | 0.109025 | 0.4199 |
| RP11-296A18.3 | -0.152757257 | 0.094755 | -1.61213 | 0.106935 | 0.4199 |
| RP11-319G9.3 | -0.168961472 | 0.105071 | -1.60808 | 0.107818 | 0.4199 |
| RP11-806L2.2 | 0.209555908 | 0.130505 | 1.605731 | 0.108333 | 0.4199 |
| RP6-191P20.4 | 0.155902495 | 0.096968 | 1.607779 | 0.107884 | 0.4199 |
| RPL34-AS1 | -0.163531286 | 0.10183 | -1.60593 | 0.10829 | 0.4199 |
| AC062028.1 | 0.159155768 | 0.099556 | 1.598663 | 0.109896 | 0.421454 |
| LINC00085 | -0.130240538 | 0.081587 | -1.59633 | 0.110415 | 0.421454 |
| RP11-2E17.1 | -0.178894045 | 0.112158 | -1.59502 | 0.110707 | 0.421454 |
| RP11-343N15.5 | -0.122900557 | 0.076903 | -1.59813 | 0.110014 | 0.421454 |
| RP11-439C15.4 | 0.149850917 | 0.093906 | 1.595761 | 0.110542 | 0.421454 |
| RP4-739H11.4 | -0.146654111 | 0.091725 | -1.59884 | 0.109857 | 0.421454 |
| RP5-1120P11.1 | 0.160775755 | 0.100763 | 1.59558 | 0.110583 | 0.421454 |
| RP6-201G10.2 | -0.169436547 | 0.106306 | -1.59386 | 0.110968 | 0.421751 |
| RP11-500G22.2 | -0.215151815 | 0.135236 | -1.59094 | 0.111623 | 0.423545 |
| RP11-713P17.3 | 0.145395377 | 0.0915 | 1.589021 | 0.112056 | 0.423791 |
| SNORA67 | -0.144993696 | 0.091202 | -1.58981 | 0.111878 | 0.423791 |
| RP13-616I3.1 | -0.183814091 | 0.115806 | -1.58726 | 0.112454 | 0.424601 |
| RP11-439E19.3 | 0.142743879 | 0.08998 | 1.586401 | 0.112648 | 0.42464 |
| CTC-297N7.5 | -0.191201841 | 0.120735 | -1.58364 | 0.113275 | 0.424918 |
| CTD-2555A7.2 | 0.157569826 | 0.099477 | 1.58398 | 0.113198 | 0.424918 |
| RP11-235E17.6 | -0.186899216 | 0.11797 | -1.5843 | 0.113127 | 0.424918 |
| CTD-2666L21.1 | 0.119546203 | 0.075634 | 1.580578 | 0.113974 | 0.425468 |
| RP11-180N14.1 | -0.144001076 | 0.091088 | -1.58091 | 0.113899 | 0.425468 |
| TAPT1-AS1 | -0.09163719 | 0.057973 | -1.58068 | 0.113951 | 0.425468 |
| LINC00342 | 0.094249539 | 0.059677 | 1.57933 | 0.11426 | 0.425846 |
| RP11-305E6.4 | -0.108603386 | 0.06881 | -1.5783 | 0.114497 | 0.426039 |
| AC004840.9 | 0.126830001 | 0.080417 | 1.577147 | 0.114762 | 0.426163 |
| NRG1-IT2 | 0.193922957 | 0.123231 | 1.573652 | 0.115568 | 0.426163 |
| RP11-317N8.5 | 0.121050645 | 0.076966 | 1.57278 | 0.11577 | 0.426163 |
| RP11-635N19.1 | -0.158998541 | 0.100891 | -1.57594 | 0.115039 | 0.426163 |
| RP11-82L18.4 | 0.141401467 | 0.089848 | 1.573782 | 0.115538 | 0.426163 |
| RP4-769N13.6 | 0.099925756 | 0.063544 | 1.572549 | 0.115823 | 0.426163 |
| ZSWIM8-AS1 | -0.169232905 | 0.107479 | -1.57456 | 0.115357 | 0.426163 |
| RP11-262I2.2 | 0.171390224 | 0.109285 | 1.568287 | 0.116814 | 0.428442 |
| RP11-88H12.2 | -0.163932352 | 0.104523 | -1.56839 | 0.116791 | 0.428442 |
| CTC-525D6.1 | 0.146481483 | 0.093838 | 1.561001 | 0.118523 | 0.429962 |
| RP11-171I2.1 | 0.144020006 | 0.092313 | 1.560119 | 0.118732 | 0.429962 |
| RP11-346I3.4 | -0.189288341 | 0.120969 | -1.56477 | 0.117638 | 0.429962 |
| RP11-395A13.2 | -0.132512713 | 0.084956 | -1.55978 | 0.118811 | 0.429962 |
| RP11-449G16.1 | -0.157887614 | 0.101207 | -1.56004 | 0.11875 | 0.429962 |
| RP11-527D7.1 | 0.151457601 | 0.096766 | 1.565188 | 0.117539 | 0.429962 |
| RP11-611E13.2 | 0.116518066 | 0.074623 | 1.561428 | 0.118423 | 0.429962 |
| RP11-612B6.2 | -0.192574762 | 0.123413 | -1.56041 | 0.118662 | 0.429962 |
| RP4-791M13.3 | -0.153724197 | 0.09858 | -1.55938 | 0.118906 | 0.429962 |
| RP11-499P20.2 | -0.137444323 | 0.088264 | -1.55719 | 0.119426 | 0.431167 |
| LINC00610 | 0.142390467 | 0.091865 | 1.549995 | 0.121143 | 0.433296 |
| RP11-195B17.1 | 0.140138502 | 0.090328 | 1.551435 | 0.120797 | 0.433296 |
| RP11-509J21.2 | -0.183613813 | 0.118274 | -1.55245 | 0.120555 | 0.433296 |
| RP11-54O7.17 | -0.201622496 | 0.130079 | -1.55 | 0.121142 | 0.433296 |
| RP11-849I19.1 | -0.155633715 | 0.100393 | -1.55024 | 0.121084 | 0.433296 |
| XXbac-BPGBPG55C20.2 | -0.126085805 | 0.081257 | -1.55169 | 0.120736 | 0.433296 |
| AL592494.5 | -0.118722454 | 0.076813 | -1.5456 | 0.122202 | 0.435734 |
| HNRNPU-AS1 | -0.106115571 | 0.068637 | -1.54605 | 0.122093 | 0.435734 |
| AC011526.1 | -0.201003139 | 0.130151 | -1.54438 | 0.122496 | 0.435988 |
| RP1-79C4.4 | -0.170529403 | 0.110465 | -1.54374 | 0.122651 | 0.435988 |
| CTC-340D7.1 | 0.156282256 | 0.101351 | 1.541994 | 0.123075 | 0.436151 |
| RP11-566K11.7 | 0.111206094 | 0.072089 | 1.542626 | 0.122921 | 0.436151 |
| CTD-2047H16.4 | 0.129441101 | 0.084016 | 1.540678 | 0.123395 | 0.436614 |
| CTD-2540M10.1 | 0.127577112 | 0.082949 | 1.538027 | 0.124042 | 0.436689 |
| LINC00094 | 0.106361553 | 0.069154 | 1.538037 | 0.12404 | 0.436689 |
| RP1-69D17.4 | 0.155835399 | 0.101357 | 1.53749 | 0.124173 | 0.436689 |
| SNHG10 | -0.110537186 | 0.07182 | -1.53908 | 0.123786 | 0.436689 |
| CTD-2314B22.3 | -0.135205824 | 0.08828 | -1.53156 | 0.125631 | 0.440474 |
| CTD-2540L5.5 | 0.11051582 | 0.072132 | 1.532126 | 0.125491 | 0.440474 |
| AC006946.16 | -0.135741236 | 0.088688 | -1.53056 | 0.125879 | 0.440673 |
| U47924.30 | -0.118181808 | 0.077258 | -1.5297 | 0.126091 | 0.440746 |
| RP1-104O17.1 | -0.186558734 | 0.12209 | -1.52805 | 0.126501 | 0.441377 |
| RP11-226L15.5 | -0.138305717 | 0.090548 | -1.52743 | 0.126654 | 0.441377 |
| PSMD5-AS1 | -0.193201539 | 0.126552 | -1.52665 | 0.126847 | 0.441382 |
| AC009501.4 | -0.165309633 | 0.108735 | -1.5203 | 0.128435 | 0.443561 |
| FLG-AS1 | -0.191565407 | 0.125992 | -1.52046 | 0.128395 | 0.443561 |
| LL0XNC01-7P3.1 | -0.133777217 | 0.087827 | -1.5232 | 0.12771 | 0.443561 |
| RP11-147L13.2 | -0.154948924 | 0.101846 | -1.5214 | 0.128159 | 0.443561 |
| RP3-467K16.4 | -0.186810057 | 0.122715 | -1.52231 | 0.127932 | 0.443561 |
| MIR219-2 | -0.204272174 | 0.134608 | -1.51753 | 0.129132 | 0.444813 |
| RP11-563K23.1 | -0.152306432 | 0.100378 | -1.51733 | 0.129183 | 0.444813 |
| CTD-2555K7.2 | -0.179868002 | 0.118853 | -1.51336 | 0.130188 | 0.44694 |
| RP1-90J20.11 | 0.143070077 | 0.094521 | 1.513634 | 0.130119 | 0.44694 |
| RP11-624C23.1 | 0.146112469 | 0.096605 | 1.512476 | 0.130413 | 0.447047 |
| A2M-AS1 | -0.150016551 | 0.099331 | -1.51027 | 0.130974 | 0.448086 |
| RP11-774O3.3 | -0.113160468 | 0.074953 | -1.50976 | 0.131105 | 0.448086 |
| CTD-2308G16.1 | -0.133734708 | 0.088737 | -1.50709 | 0.131789 | 0.448467 |
| NRG1-IT1 | 0.146542528 | 0.097275 | 1.506483 | 0.131943 | 0.448467 |
| RP11-706O15.3 | -0.192701735 | 0.127932 | -1.50629 | 0.131994 | 0.448467 |
| SETD5-AS1 | -0.09925441 | 0.065871 | -1.50679 | 0.131865 | 0.448467 |
| DGCR5 | 0.151310994 | 0.100531 | 1.505117 | 0.132294 | 0.448826 |
| RP11-426C22.5 | -0.163089705 | 0.108706 | -1.50028 | 0.133542 | 0.452394 |
| AF146191.4 | -0.14804047 | 0.098815 | -1.49815 | 0.134094 | 0.45346 |
| SNHG3 | 0.121912275 | 0.081408 | 1.497554 | 0.134249 | 0.45346 |
| RP3-462C17.1 | 0.173434218 | 0.115897 | 1.496456 | 0.134535 | 0.45376 |
| RP11-15H20.7 | 0.121457277 | 0.081252 | 1.49482 | 0.134961 | 0.45452 |
| RP5-935K16.1 | -0.084641181 | 0.056651 | -1.49408 | 0.135154 | 0.45452 |
| MIR145 | -0.185158657 | 0.124265 | -1.49003 | 0.136217 | 0.456098 |
| RP11-999E24.3 | -0.120781398 | 0.081025 | -1.49067 | 0.136048 | 0.456098 |
| SEC24B-AS1 | 0.109560549 | 0.073511 | 1.490405 | 0.136118 | 0.456098 |
| RP11-1299A16.3 | -0.183323834 | 0.123105 | -1.48916 | 0.136445 | 0.4562 |
| DYNLL1-AS1 | 0.11551592 | 0.077666 | 1.487348 | 0.136923 | 0.457137 |
| RP11-503P10.1 | 0.131107429 | 0.088506 | 1.481342 | 0.138516 | 0.460454 |
| RP11-752G15.3 | -0.178386052 | 0.120413 | -1.48146 | 0.138485 | 0.460454 |
| hsa-mir-125a | -0.14743537 | 0.099495 | -1.48184 | 0.138382 | 0.460454 |
| NAV2-IT1 | -0.151529796 | 0.102353 | -1.48046 | 0.13875 | 0.460571 |
| MAFG-AS1 | 0.147481289 | 0.099962 | 1.475375 | 0.140112 | 0.463756 |
| RP11-1275H24.1 | 0.147079995 | 0.09967 | 1.475672 | 0.140032 | 0.463756 |
| LINC00621 | 0.159878024 | 0.10855 | 1.472845 | 0.140793 | 0.464912 |
| NOP14-AS1 | 0.098936599 | 0.067186 | 1.472581 | 0.140864 | 0.464912 |
| RP11-337L12.1 | -0.162292057 | 0.110485 | -1.46891 | 0.141858 | 0.467523 |
| LINC01018 | 0.151329588 | 0.103097 | 1.467844 | 0.142147 | 0.467806 |
| CTA-292E10.6 | -0.154237035 | 0.105173 | -1.46651 | 0.142509 | 0.46833 |
| RP11-440D17.3 | -0.151445091 | 0.103338 | -1.46553 | 0.142778 | 0.468547 |
| AF131215.4 | -0.141049637 | 0.096335 | -1.46416 | 0.14315 | 0.469099 |
| RP4-669H2.1 | -0.123753029 | 0.084606 | -1.4627 | 0.14355 | 0.469745 |
| RP11-728G15.1 | 0.175587246 | 0.120131 | 1.461633 | 0.143842 | 0.470033 |
| AC127904.2 | 0.125059838 | 0.08573 | 1.458772 | 0.144628 | 0.470228 |
| AF131215.6 | 0.153832833 | 0.105798 | 1.454023 | 0.14594 | 0.470228 |
| CTC-273B12.8 | -0.158111771 | 0.108626 | -1.45557 | 0.145513 | 0.470228 |
| CTD-2619J13.19 | -0.130994223 | 0.090065 | -1.45445 | 0.145822 | 0.470228 |
| KTN1-AS1 | 0.100088464 | 0.068526 | 1.460601 | 0.144125 | 0.470228 |
| RP11-567L7.6 | 0.16630889 | 0.114105 | 1.457506 | 0.144977 | 0.470228 |
| RP11-644F5.11 | -0.134183922 | 0.092249 | -1.45459 | 0.145782 | 0.470228 |
| RP11-65L3.2 | -0.14455311 | 0.099253 | -1.45641 | 0.14528 | 0.470228 |
| RP11-680G24.5 | 0.191764546 | 0.13161 | 1.457067 | 0.145098 | 0.470228 |
| RP11-946L20.4 | 0.107305798 | 0.073693 | 1.45611 | 0.145362 | 0.470228 |
| CTD-3148I10.15 | -0.152596002 | 0.10517 | -1.45094 | 0.146796 | 0.471012 |
| HCG11 | -0.087293884 | 0.060124 | -1.45189 | 0.146533 | 0.471012 |
| RP11-485G4.2 | -0.087427864 | 0.060241 | -1.45129 | 0.146699 | 0.471012 |
| RP1-74M1.3 | 0.134668947 | 0.093028 | 1.447612 | 0.147726 | 0.473337 |
| CTB-31O20.2 | -0.129414112 | 0.089623 | -1.44399 | 0.148742 | 0.473991 |
| FAM181A-AS1 | -0.142421119 | 0.098531 | -1.44544 | 0.148334 | 0.473991 |
| RP11-37B2.1 | -0.093429953 | 0.064737 | -1.44323 | 0.148957 | 0.473991 |
| RP11-597D13.9 | -0.187607795 | 0.129991 | -1.44323 | 0.148954 | 0.473991 |
| SNHG6 | -0.141938043 | 0.098254 | -1.4446 | 0.148569 | 0.473991 |
| AC133528.2 | -0.12325211 | 0.085534 | -1.44096 | 0.149595 | 0.474791 |
| RP11-642D21.1 | -0.162353184 | 0.112677 | -1.44088 | 0.14962 | 0.474791 |
| RP11-115C21.2 | -0.109929017 | 0.076414 | -1.4386 | 0.150264 | 0.476179 |
| CTD-2517M22.14 | 0.133264188 | 0.092771 | 1.436488 | 0.150864 | 0.477424 |
| AC092168.2 | -0.164467663 | 0.114568 | -1.43554 | 0.151133 | 0.477621 |
| AC084018.1 | -0.104098669 | 0.072662 | -1.43265 | 0.151957 | 0.47957 |
| FBXL19-AS1 | -0.142031116 | 0.099323 | -1.42999 | 0.15272 | 0.481106 |
| RP11-9G1.3 | -0.111872303 | 0.07826 | -1.4295 | 0.152861 | 0.481106 |
| CTD-2083E4.4 | -0.13379982 | 0.093922 | -1.42459 | 0.154277 | 0.483873 |
| MIR31HG | -0.178566037 | 0.125382 | -1.42417 | 0.154396 | 0.483873 |
| RP11-312O7.2 | -0.156549391 | 0.109819 | -1.42553 | 0.154005 | 0.483873 |
| RP11-461O7.1 | -0.083929561 | 0.058981 | -1.423 | 0.154735 | 0.483873 |
| RP11-55K13.1 | 0.136294445 | 0.095839 | 1.422126 | 0.15499 | 0.483873 |
| RP11-584P21.2 | 0.146342309 | 0.102906 | 1.422095 | 0.154999 | 0.483873 |
| CTD-2020K17.1 | 0.102278012 | 0.071969 | 1.421143 | 0.155275 | 0.484081 |
| RP1-257A7.4 | -0.134397264 | 0.09494 | -1.41559 | 0.156894 | 0.487973 |
| RP11-757O6.1 | 0.16332899 | 0.115393 | 1.415416 | 0.156947 | 0.487973 |
| AC011747.7 | -0.14260699 | 0.101218 | -1.4089 | 0.158863 | 0.489076 |
| AC016629.8 | -0.132595109 | 0.094014 | -1.41038 | 0.158428 | 0.489076 |
| AC074138.3 | 0.098928748 | 0.070172 | 1.409796 | 0.1586 | 0.489076 |
| MAGI2-IT1 | -0.153282494 | 0.108896 | -1.4076 | 0.159249 | 0.489076 |
| RP1-111C20.4 | 0.1161732 | 0.082707 | 1.404639 | 0.160129 | 0.489076 |
| RP11-31F15.2 | -0.136078656 | 0.096684 | -1.40746 | 0.159291 | 0.489076 |
| RP11-370I10.6 | -0.139478774 | 0.098992 | -1.40899 | 0.158838 | 0.489076 |
| RP11-423O2.5 | -0.187854043 | 0.133578 | -1.40632 | 0.159629 | 0.489076 |
| RP11-536K7.3 | -0.150441224 | 0.107093 | -1.40477 | 0.16009 | 0.489076 |
| RP11-65J3.1 | 0.121970608 | 0.086776 | 1.405578 | 0.15985 | 0.489076 |
| RP11-742D12.2 | 0.147410698 | 0.104981 | 1.404166 | 0.160269 | 0.489076 |
| RP5-1074L1.4 | -0.113888717 | 0.080911 | -1.40759 | 0.159253 | 0.489076 |
| SPON1 | -0.15123599 | 0.107604 | -1.40549 | 0.159877 | 0.489076 |
| TMEM191C | 0.145273851 | 0.10317 | 1.4081 | 0.159101 | 0.489076 |
| GAS6-AS2 | 0.127900002 | 0.091348 | 1.400138 | 0.161472 | 0.489509 |
| GUSBP11 | 0.076430895 | 0.054573 | 1.400524 | 0.161356 | 0.489509 |
| RP11-20G6.3 | -0.10486097 | 0.074844 | -1.40107 | 0.161194 | 0.489509 |
| RP11-811P12.3 | 0.1030409 | 0.073521 | 1.401522 | 0.161058 | 0.489509 |
| RP3-468B3.2 | -0.113617452 | 0.08106 | -1.40165 | 0.161021 | 0.489509 |
| AC006115.3 | 0.09502955 | 0.067935 | 1.398839 | 0.161861 | 0.489779 |
| RP11-23J9.4 | 0.093762016 | 0.067068 | 1.398008 | 0.162111 | 0.489779 |
| RP11-410E4.1 | -0.151616338 | 0.108529 | -1.39701 | 0.162411 | 0.489779 |
| RP11-457M11.2 | -0.126724866 | 0.090684 | -1.39744 | 0.162282 | 0.489779 |
| AC004158.3 | -0.163199967 | 0.116933 | -1.39567 | 0.162815 | 0.490359 |
| NUTM2A-AS1 | -0.120073169 | 0.086126 | -1.39416 | 0.163269 | 0.49063 |
| RP11-120J1.1 | -0.174944919 | 0.125502 | -1.39396 | 0.163331 | 0.49063 |
| CTB-152G17.6 | -0.107820462 | 0.077526 | -1.39077 | 0.164296 | 0.491098 |
| PAXIP1-AS1 | 0.08906579 | 0.06397 | 1.392311 | 0.163828 | 0.491098 |
| RP11-339B21.14 | 0.147879874 | 0.106309 | 1.39104 | 0.164213 | 0.491098 |
| RP11-708H21.4 | -0.137684402 | 0.099009 | -1.39063 | 0.164338 | 0.491098 |
| AC005152.2 | -0.120620984 | 0.087262 | -1.38228 | 0.166885 | 0.491603 |
| AC005618.6 | -0.10677059 | 0.077045 | -1.38582 | 0.165803 | 0.491603 |
| AC147651.3 | 0.147569706 | 0.106727 | 1.382681 | 0.166763 | 0.491603 |
| AP006216.11 | 0.087640326 | 0.063529 | 1.379536 | 0.167729 | 0.491603 |
| CTB-36H16.2 | -0.105361133 | 0.076629 | -1.37494 | 0.169149 | 0.491603 |
| CTC-304I17.4 | 0.153474769 | 0.111557 | 1.375751 | 0.168899 | 0.491603 |
| CTD-2283N19.1 | -0.142586242 | 0.103669 | -1.3754 | 0.169007 | 0.491603 |
| EPB41L4A-AS1 | 0.104723501 | 0.075946 | 1.378921 | 0.167919 | 0.491603 |
| LINC00623 | 0.144620614 | 0.104493 | 1.384018 | 0.166353 | 0.491603 |
| RP11-238K6.1 | 0.185085572 | 0.134608 | 1.374997 | 0.169132 | 0.491603 |
| RP11-304L19.5 | -0.125521229 | 0.091015 | -1.37912 | 0.167856 | 0.491603 |
| RP11-31I22.1 | 0.160609622 | 0.116778 | 1.375339 | 0.169027 | 0.491603 |
| RP11-32B5.7 | 0.187133102 | 0.13555 | 1.380546 | 0.167419 | 0.491603 |
| RP11-391M1.4 | -0.101022573 | 0.07274 | -1.38882 | 0.164887 | 0.491603 |
| RP11-395G23.3 | 0.123663284 | 0.089509 | 1.381577 | 0.167102 | 0.491603 |
| RP11-552D4.1 | -0.186166224 | 0.134949 | -1.37953 | 0.167731 | 0.491603 |
| RP11-640M9.1 | -0.120918749 | 0.087446 | -1.38278 | 0.166731 | 0.491603 |
| RP13-726E6.2 | 0.1422989 | 0.103506 | 1.374795 | 0.169195 | 0.491603 |
| RP3-368A4.6 | -0.10478748 | 0.076127 | -1.37648 | 0.168672 | 0.491603 |
| RP5-1159O4.1 | -0.145800992 | 0.105001 | -1.38857 | 0.164964 | 0.491603 |
| SDCBP2-AS1 | -0.118303139 | 0.085535 | -1.3831 | 0.166633 | 0.491603 |
| TCL6 | -0.129240973 | 0.093394 | -1.38382 | 0.166413 | 0.491603 |
| AC019118.2 | 0.146426657 | 0.106575 | 1.37393 | 0.169463 | 0.491736 |
| LINC00893 | 0.096446723 | 0.07025 | 1.372912 | 0.16978 | 0.491736 |
| RP11-137L10.6 | -0.126968383 | 0.092549 | -1.3719 | 0.170093 | 0.491736 |
| RP11-407N17.5 | 0.152608986 | 0.111193 | 1.372474 | 0.169916 | 0.491736 |
| RP11-320H14.1 | -0.132184766 | 0.096401 | -1.3712 | 0.170312 | 0.491751 |
| AC003104.1 | -0.141287584 | 0.103212 | -1.36891 | 0.171027 | 0.491838 |
| RP11-15A1.3 | 0.093033174 | 0.068056 | 1.367014 | 0.171621 | 0.491838 |
| RP11-2B6.2 | -0.142953988 | 0.104526 | -1.36764 | 0.171425 | 0.491838 |
| RP11-362K14.6 | -0.125999839 | 0.092087 | -1.36827 | 0.171228 | 0.491838 |
| RP11-467D6.1 | -0.110496394 | 0.080793 | -1.36764 | 0.171424 | 0.491838 |
| RP4-809F18.1 | 0.157050014 | 0.114847 | 1.367471 | 0.171478 | 0.491838 |
| RP11-727A23.4 | -0.131086305 | 0.095982 | -1.36574 | 0.172019 | 0.492368 |
| LINC00284 | -0.15737595 | 0.115594 | -1.36146 | 0.173369 | 0.493781 |
| RAP2C-AS1 | -0.113351541 | 0.083141 | -1.36336 | 0.172768 | 0.493781 |
| RP11-173M1.8 | -0.142313835 | 0.10452 | -1.36159 | 0.173327 | 0.493781 |
| RP3-475N16.1 | 0.089523945 | 0.065693 | 1.362765 | 0.172957 | 0.493781 |
| AC132217.4 | -0.169942837 | 0.125111 | -1.35834 | 0.174357 | 0.494815 |
| CTD-2308L22.1 | 0.162217706 | 0.119539 | 1.357032 | 0.174771 | 0.494815 |
| CTD-2622I13.3 | -0.128784544 | 0.094827 | -1.3581 | 0.174432 | 0.494815 |
| FAM226B | 0.114147975 | 0.084036 | 1.358327 | 0.17436 | 0.494815 |
| RP11-423G4.7 | 0.141890218 | 0.104567 | 1.356926 | 0.174805 | 0.494815 |
| AC007283.5 | -0.120266127 | 0.088788 | -1.35454 | 0.175565 | 0.495144 |
| RP1-140C12.2 | -0.158974454 | 0.117297 | -1.35532 | 0.175316 | 0.495144 |
| RP3-508I15.9 | -0.125627964 | 0.092706 | -1.35513 | 0.175377 | 0.495144 |
| RP11-10L12.4 | -0.120660298 | 0.089363 | -1.35023 | 0.176942 | 0.497811 |
| RP11-37C7.1 | 0.09474401 | 0.07014 | 1.350777 | 0.176767 | 0.497811 |
| RABGAP1L-IT1 | -0.103352915 | 0.076619 | -1.34893 | 0.17736 | 0.498379 |
| RP11-219A15.4 | 0.16536994 | 0.122917 | 1.345376 | 0.178504 | 0.500375 |
| TRIM36-IT1 | 0.153656253 | 0.114167 | 1.345895 | 0.178337 | 0.500375 |
| RP11-439L18.3 | 0.112803249 | 0.083913 | 1.344295 | 0.178853 | 0.500745 |
| RP11-262H14.1 | -0.12894812 | 0.096022 | -1.3429 | 0.179305 | 0.501403 |
| RP11-196G18.24 | -0.138601929 | 0.103273 | -1.34209 | 0.179567 | 0.501527 |
| AC131025.8 | -0.165142507 | 0.123343 | -1.33889 | 0.180607 | 0.503212 |
| RP11-473M20.7 | 0.112921061 | 0.084339 | 1.338895 | 0.180605 | 0.503212 |
| AF131217.1 | -0.163103935 | 0.121941 | -1.33757 | 0.181037 | 0.503803 |
| ENTPD1-AS1 | -0.079970469 | 0.05994 | -1.33419 | 0.182143 | 0.50627 |
| RP11-662M24.2 | 0.136413971 | 0.102449 | 1.331537 | 0.183012 | 0.508074 |
| CTB-31N19.3 | -0.137867676 | 0.103709 | -1.32937 | 0.183726 | 0.509288 |
| VAC14-AS1 | -0.151206487 | 0.113786 | -1.32887 | 0.183891 | 0.509288 |
| CTC-559E9.6 | -0.128634435 | 0.096951 | -1.32679 | 0.184577 | 0.509963 |
| TTTY14 | -0.09363247 | 0.070562 | -1.32696 | 0.184522 | 0.509963 |
| RP11-206L10.2 | -0.145900375 | 0.110064 | -1.3256 | 0.184973 | 0.510447 |
| DHRS4-AS1 | -0.091444538 | 0.069086 | -1.32363 | 0.185626 | 0.510719 |
| MCM3AP-AS1 | -0.114033384 | 0.086174 | -1.3233 | 0.185736 | 0.510719 |
| RP11-412D9.4 | -0.11840089 | 0.089441 | -1.32378 | 0.185576 | 0.510719 |
| AC002117.1 | -0.137650344 | 0.104287 | -1.31992 | 0.186861 | 0.511981 |
| AC007563.5 | 0.155171343 | 0.117478 | 1.320852 | 0.186551 | 0.511981 |
| HAR1A | 0.148968773 | 0.112832 | 1.32027 | 0.186745 | 0.511981 |
| RP11-216L13.19 | 0.10390217 | 0.079024 | 1.314819 | 0.188571 | 0.516053 |
| RP11-304F15.7 | 0.13603615 | 0.103549 | 1.313734 | 0.188936 | 0.51644 |
| LINC00663 | -0.079589714 | 0.060735 | -1.31044 | 0.190046 | 0.517978 |
| RP11-88I18.2 | -0.074145087 | 0.05657 | -1.31068 | 0.189965 | 0.517978 |
| RP4-773N10.4 | -0.08760748 | 0.066872 | -1.31007 | 0.190172 | 0.517978 |
| AC026150.8 | -0.108669439 | 0.083009 | -1.30913 | 0.190491 | 0.518235 |
| RAB11B-AS1 | -0.149355939 | 0.114204 | -1.3078 | 0.190941 | 0.518847 |
| CTC-436K13.3 | 0.121782482 | 0.093238 | 1.306141 | 0.191505 | 0.519766 |
| AP002954.3 | -0.171962577 | 0.132005 | -1.3027 | 0.192677 | 0.522303 |
| RP11-521B24.3 | 0.08619798 | 0.066201 | 1.302071 | 0.192892 | 0.522303 |
| AC069277.2 | -0.133076321 | 0.102359 | -1.30009 | 0.193569 | 0.522908 |
| RP11-427H3.3 | -0.07181153 | 0.055212 | -1.30064 | 0.19338 | 0.522908 |
| RP1-193H18.2 | -0.095793854 | 0.073817 | -1.29772 | 0.194384 | 0.524495 |
| AC008174.3 | -0.133080161 | 0.102773 | -1.2949 | 0.195356 | 0.526503 |
| CACNA1C-IT3 | 0.137255178 | 0.106202 | 1.292394 | 0.196221 | 0.528118 |
| ZEB2-AS1 | -0.149412952 | 0.115659 | -1.29184 | 0.196413 | 0.528118 |
| ACTA2-AS1 | 0.119195394 | 0.09251 | 1.28846 | 0.197586 | 0.528901 |
| DGCR9 | 0.13995151 | 0.10856 | 1.289165 | 0.197341 | 0.528901 |
| MIR143HG | -0.152347427 | 0.118296 | -1.28785 | 0.197798 | 0.528901 |
| RP11-155D18.12 | -0.122551729 | 0.09522 | -1.28704 | 0.19808 | 0.528901 |
| RP13-487P22.1 | -0.128999211 | 0.100227 | -1.28708 | 0.198068 | 0.528901 |
| RP5-864K19.4 | 0.09157993 | 0.071098 | 1.288089 | 0.197715 | 0.528901 |
| RP4-740C4.6 | 0.135284238 | 0.105254 | 1.285313 | 0.198683 | 0.529898 |
| CTD-3018O17.3 | -0.117742671 | 0.09171 | -1.28386 | 0.199192 | 0.530641 |
| TMCC1-AS1 | 0.088674029 | 0.06911 | 1.283078 | 0.199465 | 0.530756 |
| AC017099.3 | -0.091997697 | 0.071875 | -1.27997 | 0.200555 | 0.530903 |
| RP11-230B22.1 | -0.137132114 | 0.107164 | -1.27964 | 0.200671 | 0.530903 |
| RP11-288H12.4 | 0.108193023 | 0.084413 | 1.281705 | 0.199946 | 0.530903 |
| RP11-586D19.1 | 0.11917287 | 0.093011 | 1.281282 | 0.200094 | 0.530903 |
| RP4-749H3.1 | -0.118610549 | 0.09269 | -1.27965 | 0.200669 | 0.530903 |
| LINC00966 | 0.126956999 | 0.099272 | 1.278883 | 0.200938 | 0.531002 |
| LINC01088 | -0.147833828 | 0.115772 | -1.27694 | 0.201622 | 0.5322 |
| AC091729.9 | 0.144240312 | 0.113026 | 1.276168 | 0.201896 | 0.532314 |
| CTD-2561J22.5 | 0.137480904 | 0.107916 | 1.273963 | 0.202677 | 0.532792 |
| RP11-364C11.3 | 0.144241588 | 0.113157 | 1.274706 | 0.202413 | 0.532792 |
| RP3-406A7.7 | -0.139289895 | 0.109359 | -1.2737 | 0.20277 | 0.532792 |
| RP11-122K13.12 | 0.119234634 | 0.093733 | 1.272066 | 0.20335 | 0.533706 |
| BCDIN3D-AS1 | 0.098782447 | 0.077748 | 1.270554 | 0.203887 | 0.534509 |
| CTD-2303H24.2 | -0.12857375 | 0.101251 | -1.26985 | 0.204138 | 0.534559 |
| SNAI3-AS1 | 0.110409967 | 0.087202 | 1.266145 | 0.205461 | 0.537414 |
| CTD-2530H12.8 | 0.14177384 | 0.112101 | 1.264701 | 0.205979 | 0.537552 |
| PAXBP1-AS1 | -0.078401704 | 0.062017 | -1.26419 | 0.206162 | 0.537552 |
| RP11-766N7.3 | 0.137114722 | 0.108473 | 1.264048 | 0.206213 | 0.537552 |
| CTD-2281E23.2 | 0.125164735 | 0.099249 | 1.261114 | 0.207268 | 0.537831 |
| LL22NC03-86G7.1 | -0.13043755 | 0.103467 | -1.26067 | 0.207427 | 0.537831 |
| RP11-143J12.2 | -0.125450789 | 0.099423 | -1.26178 | 0.207027 | 0.537831 |
| RP11-168K9.2 | 0.148562536 | 0.117689 | 1.26233 | 0.20683 | 0.537831 |
| RP11-97O12.6 | -0.119853725 | 0.095084 | -1.26051 | 0.207486 | 0.537831 |
| AC007620.3 | -0.110214913 | 0.087646 | -1.25751 | 0.20857 | 0.538399 |
| LINC00926 | -0.143614726 | 0.114299 | -1.25649 | 0.20894 | 0.538399 |
| RP1-78O14.1 | -0.126227643 | 0.10054 | -1.25549 | 0.2093 | 0.538399 |
| RP11-202H2.1 | 0.108978265 | 0.086833 | 1.255026 | 0.209469 | 0.538399 |
| RP11-81N13.1 | -0.133664345 | 0.106452 | -1.25563 | 0.209251 | 0.538399 |
| RP4-621B10.8 | 0.119819958 | 0.095279 | 1.257568 | 0.208548 | 0.538399 |
| RP4-714D9.5 | -0.088743427 | 0.07048 | -1.25913 | 0.207982 | 0.538399 |
| RP5-890O3.9 | -0.099100116 | 0.078998 | -1.25446 | 0.209676 | 0.538399 |
| RP6-91H8.5 | 0.137443128 | 0.109595 | 1.254101 | 0.209805 | 0.538399 |
| CDC42-IT1 | -0.133354861 | 0.106449 | -1.25276 | 0.210292 | 0.538493 |
| RP11-866E20.3 | 0.114272755 | 0.09122 | 1.252717 | 0.210309 | 0.538493 |
| RP5-1065J22.8 | -0.116435497 | 0.093011 | -1.25185 | 0.210624 | 0.538702 |
| RP11-529H2.2 | -0.114161722 | 0.091308 | -1.2503 | 0.211191 | 0.539556 |
| RP11-37E23.5 | 0.118306752 | 0.094673 | 1.249642 | 0.211431 | 0.539569 |
| AP003419.11 | -0.113871017 | 0.091245 | -1.24797 | 0.212042 | 0.539936 |
| RP11-324I22.4 | 0.098048293 | 0.07855 | 1.248223 | 0.21195 | 0.539936 |
| RP11-290D2.6 | -0.149294595 | 0.119722 | -1.24701 | 0.212394 | 0.540236 |
| RP11-888D10.4 | -0.141611189 | 0.113729 | -1.24517 | 0.21307 | 0.541357 |
| RP11-966I7.1 | -0.097103588 | 0.078076 | -1.2437 | 0.21361 | 0.542131 |
| SNHG1 | -0.115071399 | 0.092889 | -1.23881 | 0.215416 | 0.546116 |
| RP11-272L13.3 | 0.148530813 | 0.12041 | 1.233542 | 0.217373 | 0.550473 |
| RP11-983P16.4 | -0.131946284 | 0.107235 | -1.23044 | 0.218534 | 0.552804 |
| CTC-552D5.1 | 0.160944492 | 0.131109 | 1.227563 | 0.219611 | 0.554921 |
| RP11-114G22.1 | 0.12596872 | 0.102689 | 1.226702 | 0.219935 | 0.55513 |
| CTB-131K11.1 | 0.119564855 | 0.097541 | 1.225793 | 0.220277 | 0.555386 |
| CTA-384D8.36 | 0.091082157 | 0.074405 | 1.224134 | 0.220902 | 0.555747 |
| RP11-798M19.6 | -0.105454487 | 0.086135 | -1.22429 | 0.220842 | 0.555747 |
| AC131056.3 | -0.166014997 | 0.136296 | -1.21805 | 0.223205 | 0.56093 |
| RP11-586D19.2 | 0.120141766 | 0.098708 | 1.217141 | 0.22355 | 0.561187 |
| AC103564.7 | 0.130103661 | 0.107066 | 1.215176 | 0.224299 | 0.56222 |
| RP11-803B1.8 | 0.117402863 | 0.096645 | 1.214782 | 0.224449 | 0.56222 |
| CTB-25J19.9 | 0.105247753 | 0.086712 | 1.213766 | 0.224837 | 0.56258 |
| RP1-90L6.2 | -0.102139211 | 0.084362 | -1.21073 | 0.225999 | 0.56477 |
| RP11-571L19.8 | 0.127842073 | 0.105637 | 1.210201 | 0.226202 | 0.56477 |
| RP11-806O11.1 | -0.159387891 | 0.131829 | -1.20905 | 0.226645 | 0.565265 |
| DTX2P1-UPK3BP1-PMS2P11 | -0.116185048 | 0.096152 | -1.20834 | 0.226915 | 0.565327 |
| DNAJC3-AS1 | -0.076082102 | 0.063199 | -1.20386 | 0.228645 | 0.567799 |
| MIR4500HG | 0.118665767 | 0.098523 | 1.204453 | 0.228415 | 0.567799 |
| RP4-798P15.3 | 0.072843946 | 0.060477 | 1.204484 | 0.228402 | 0.567799 |
| RP11-23J9.5 | 0.083161558 | 0.06917 | 1.202276 | 0.229257 | 0.568126 |
| RP4-784A16.5 | 0.131883233 | 0.109698 | 1.202242 | 0.22927 | 0.568126 |
| MEG8 | 0.099197093 | 0.082576 | 1.201287 | 0.22964 | 0.568333 |
| RP11-343L5.2 | -0.144545185 | 0.120379 | -1.20076 | 0.229846 | 0.568333 |
| AC092594.1 | 0.108373846 | 0.090733 | 1.194427 | 0.232311 | 0.568602 |
| ARHGEF7-AS2 | 0.122487995 | 0.102589 | 1.193966 | 0.232491 | 0.568602 |
| CTD-2410N18.3 | 0.119599012 | 0.100173 | 1.193924 | 0.232508 | 0.568602 |
| DLGAP1-AS4 | 0.121925026 | 0.101861 | 1.196971 | 0.231318 | 0.568602 |
| RP11-106M3.2 | 0.057398955 | 0.048092 | 1.19352 | 0.232666 | 0.568602 |
| RP11-197P3.5 | -0.116314843 | 0.097109 | -1.19777 | 0.231005 | 0.568602 |
| RP11-531A24.3 | -0.114076421 | 0.095418 | -1.19555 | 0.231872 | 0.568602 |
| RP11-696N14.1 | -0.143660257 | 0.119966 | -1.19751 | 0.231107 | 0.568602 |
| RP11-884K10.7 | -0.091007832 | 0.07611 | -1.19574 | 0.231797 | 0.568602 |
| RP11-91J19.4 | -0.073978403 | 0.061683 | -1.19933 | 0.2304 | 0.568602 |
| RP5-1107A17.4 | 0.141105999 | 0.117685 | 1.199011 | 0.230524 | 0.568602 |
| AC005943.5 | -0.116799255 | 0.098075 | -1.19092 | 0.233685 | 0.569616 |
| AC144833.1 | 0.134848582 | 0.113443 | 1.188689 | 0.234562 | 0.569616 |
| LINC00504 | 0.077831614 | 0.065452 | 1.189133 | 0.234387 | 0.569616 |
| RP11-256I9.3 | 0.140349827 | 0.117808 | 1.191347 | 0.233517 | 0.569616 |
| RP11-310E22.5 | 0.147530348 | 0.124007 | 1.189692 | 0.234168 | 0.569616 |
| RP6-206I17.3 | -0.107624866 | 0.090418 | -1.1903 | 0.233927 | 0.569616 |
| MIR600HG | 0.103677403 | 0.087322 | 1.187294 | 0.235112 | 0.570035 |
| PITPNA-AS1 | -0.121887887 | 0.102686 | -1.187 | 0.235229 | 0.570035 |
| ZNF571-AS1 | -0.114608339 | 0.096641 | -1.18591 | 0.235657 | 0.570472 |
| C20orf203 | 0.112895588 | 0.095352 | 1.183989 | 0.236417 | 0.571644 |
| CTD-2319I12.4 | 0.092447048 | 0.07825 | 1.181428 | 0.237433 | 0.571644 |
| CTD-2350C19.1 | 0.09743069 | 0.082477 | 1.181307 | 0.237481 | 0.571644 |
| CTD-3185P2.1 | -0.100615432 | 0.0852 | -1.18094 | 0.237627 | 0.571644 |
| RP11-381K20.2 | -0.095635349 | 0.080942 | -1.18152 | 0.237395 | 0.571644 |
| TMEM161B-AS1 | -0.078630438 | 0.066491 | -1.18257 | 0.236981 | 0.571644 |
| CTC-265F19.1 | -0.135826831 | 0.115115 | -1.17992 | 0.238031 | 0.572019 |
| RP11-15H20.6 | 0.084879851 | 0.072043 | 1.178184 | 0.238723 | 0.572489 |
| RP11-16C1.2 | -0.105381938 | 0.089405 | -1.17871 | 0.238514 | 0.572489 |
| LMCD1-AS1 | -0.120811029 | 0.10263 | -1.17715 | 0.239135 | 0.572882 |
| RP11-624L4.1 | 0.114778072 | 0.097643 | 1.175483 | 0.239801 | 0.573881 |
| RP11-542M13.2 | 0.086912227 | 0.074017 | 1.174212 | 0.24031 | 0.573908 |
| RP4-798A10.2 | -0.141681909 | 0.120632 | -1.17449 | 0.240197 | 0.573908 |
| RP11-680C21.1 | -0.118051647 | 0.100829 | -1.17081 | 0.241677 | 0.576575 |
| CTA-211A9.5 | -0.093240884 | 0.079884 | -1.1672 | 0.243128 | 0.578243 |
| CTD-2536I1.1 | 0.093006414 | 0.079663 | 1.167492 | 0.243012 | 0.578243 |
| RP11-418J17.1 | -0.093387411 | 0.079936 | -1.16827 | 0.242696 | 0.578243 |
| LINC00680 | 0.075570758 | 0.064801 | 1.166188 | 0.243538 | 0.578623 |
| RP11-405A12.2 | 0.115618502 | 0.099283 | 1.164531 | 0.244209 | 0.579619 |
| AC005484.5 | 0.081139697 | 0.069874 | 1.161229 | 0.245549 | 0.581688 |
| RNF157-AS1 | -0.141474198 | 0.122036 | -1.15928 | 0.246341 | 0.581688 |
| RP11-174G6.5 | -0.079769302 | 0.068737 | -1.1605 | 0.245845 | 0.581688 |
| RP11-326C3.11 | -0.13716938 | 0.118261 | -1.15989 | 0.246095 | 0.581688 |
| RP11-6N17.9 | -0.077549615 | 0.066789 | -1.16112 | 0.245595 | 0.581688 |
| MIR381HG | 0.122779922 | 0.105999 | 1.158317 | 0.246735 | 0.581799 |
| NPPA-AS1 | 0.078732641 | 0.068044 | 1.15709 | 0.247235 | 0.581799 |
| RP4-738P15.1 | 0.12973331 | 0.112159 | 1.15669 | 0.247399 | 0.581799 |
| RP4-799P18.4 | 0.137363567 | 0.118819 | 1.156078 | 0.247649 | 0.581799 |
| STARD4-AS1 | 0.122159276 | 0.105545 | 1.15741 | 0.247105 | 0.581799 |
| CTB-89H12.4 | -0.078753779 | 0.068158 | -1.15546 | 0.247901 | 0.581799 |
| DCTN1-AS1 | 0.092264579 | 0.079926 | 1.154368 | 0.248349 | 0.581826 |
| RP11-33N16.3 | -0.095565316 | 0.082817 | -1.15393 | 0.248529 | 0.581826 |
| RP11-732M18.3 | -0.109445327 | 0.094874 | -1.15359 | 0.24867 | 0.581826 |
| HCG23 | 0.140446819 | 0.122099 | 1.150268 | 0.250033 | 0.583864 |
| RP11-867G23.3 | -0.095582519 | 0.083143 | -1.14962 | 0.2503 | 0.583864 |
| RP5-1126H10.2 | 0.059251138 | 0.051534 | 1.149739 | 0.250251 | 0.583864 |
| RP11-379F4.4 | 0.101326768 | 0.088289 | 1.147666 | 0.251106 | 0.585154 |
| RP11-744K17.1 | -0.141107214 | 0.123104 | -1.14624 | 0.251694 | 0.585932 |
| RP11-175O19.4 | -0.081473241 | 0.071201 | -1.14427 | 0.252512 | 0.587244 |
| CTD-2086O20.3 | -0.100606921 | 0.088007 | -1.14317 | 0.252969 | 0.587434 |
| RP1-151F17.1 | 0.115197217 | 0.100799 | 1.142844 | 0.253103 | 0.587434 |
| RP11-531A24.5 | -0.088488006 | 0.077508 | -1.14166 | 0.253595 | 0.587984 |
| RP11-127B20.2 | -0.100026164 | 0.087738 | -1.14006 | 0.254263 | 0.588941 |
| RP4-614O4.11 | 0.084332976 | 0.074074 | 1.138489 | 0.254916 | 0.589861 |
| RP11-680F8.4 | -0.102085685 | 0.089791 | -1.13693 | 0.255569 | 0.590779 |
| AC073479.1 | -0.107507294 | 0.094663 | -1.13569 | 0.256086 | 0.591382 |
| CTC-277H1.7 | 0.10392396 | 0.091726 | 1.132985 | 0.257221 | 0.592529 |
| RP11-181E10.3 | 0.141301478 | 0.124751 | 1.132669 | 0.257353 | 0.592529 |
| RP11-458N5.1 | -0.112233486 | 0.098987 | -1.13382 | 0.256869 | 0.592529 |
| AC009404.2 | -0.096580873 | 0.08551 | -1.12947 | 0.258698 | 0.592835 |
| RP1-212P9.2 | 0.110287269 | 0.097611 | 1.129867 | 0.258532 | 0.592835 |
| RP11-147L13.8 | -0.108979352 | 0.096495 | -1.12938 | 0.258736 | 0.592835 |
| RP11-147N17.1 | 0.112705367 | 0.099783 | 1.129506 | 0.258684 | 0.592835 |
| RP11-445H22.3 | 0.118929046 | 0.105312 | 1.1293 | 0.258771 | 0.592835 |
| LINC00515 | -0.09618623 | 0.085315 | -1.12742 | 0.259563 | 0.593919 |
| SAPCD1-AS1 | 0.134194004 | 0.119076 | 1.126961 | 0.259759 | 0.593919 |
| NR2F2-AS1 | -0.122939879 | 0.109223 | -1.12558 | 0.260342 | 0.594663 |
| RP11-299M14.2 | -0.082170384 | 0.073082 | -1.12436 | 0.260861 | 0.595259 |
| MKLN1-AS1 | 0.0737641 | 0.065689 | 1.122935 | 0.261465 | 0.595459 |
| RP11-664H17.1 | 0.120201913 | 0.107001 | 1.123371 | 0.26128 | 0.595459 |
| RNF139-AS1 | 0.113811049 | 0.101456 | 1.121776 | 0.261958 | 0.595793 |
| RP11-435B5.4 | -0.143646151 | 0.128098 | -1.12138 | 0.262128 | 0.595793 |
| AC068490.1 | 0.147204854 | 0.131618 | 1.118423 | 0.263386 | 0.596371 |
| ADAM20P1 | 0.102180312 | 0.091356 | 1.11848 | 0.263362 | 0.596371 |
| RP11-57H14.3 | 0.112578299 | 0.100664 | 1.118352 | 0.263417 | 0.596371 |
| TP73-AS1 | -0.070859422 | 0.063308 | -1.11928 | 0.263022 | 0.596371 |
| AC074286.1 | -0.067794907 | 0.06066 | -1.11762 | 0.26373 | 0.596495 |
| DBH-AS1 | 0.107512593 | 0.096297 | 1.116464 | 0.264224 | 0.597026 |
| PVRL3-AS1 | 0.104595619 | 0.093863 | 1.114338 | 0.265134 | 0.597697 |
| RP11-271F18.4 | 0.128483436 | 0.115273 | 1.1146 | 0.265022 | 0.597697 |
| RP11-350N15.6 | 0.127661498 | 0.114627 | 1.113711 | 0.265403 | 0.597697 |
| RP11-529H20.5 | -0.076548045 | 0.068755 | -1.11335 | 0.265557 | 0.597697 |
| AF064858.8 | 0.131540899 | 0.11846 | 1.110424 | 0.266816 | 0.5996 |
| RP11-15N24.4 | 0.087170251 | 0.078519 | 1.110179 | 0.266922 | 0.5996 |
| RP4-758J24.5 | -0.081524341 | 0.073487 | -1.10938 | 0.267268 | 0.599794 |
| CTD-2201E18.3 | -0.115008173 | 0.103794 | -1.10804 | 0.267845 | 0.600504 |
| RP11-736K20.5 | 0.100267931 | 0.090581 | 1.106942 | 0.268319 | 0.600982 |
| RP3-414A15.10 | -0.094584928 | 0.085495 | -1.10632 | 0.268587 | 0.600998 |
| BOLA3-AS1 | 0.080035372 | 0.072506 | 1.103848 | 0.269659 | 0.602814 |
| RP11-97C16.1 | -0.104733258 | 0.095071 | -1.10163 | 0.270624 | 0.604385 |
| AC012593.1 | -0.148174739 | 0.135143 | -1.09643 | 0.272889 | 0.605428 |
| AF127936.7 | -0.086007389 | 0.078449 | -1.09634 | 0.272928 | 0.605428 |
| CTD-2277K2.1 | 0.10025367 | 0.091252 | 1.098646 | 0.271922 | 0.605428 |
| LPP-AS2 | -0.11358553 | 0.103534 | -1.09709 | 0.272604 | 0.605428 |
| RP11-195F19.9 | -0.104839946 | 0.095519 | -1.09759 | 0.272385 | 0.605428 |
| RP11-53O19.3 | -0.074668076 | 0.067942 | -1.099 | 0.271768 | 0.605428 |
| RP5-1007M22.2 | -0.104566291 | 0.095298 | -1.09725 | 0.272531 | 0.605428 |
| RP11-370K11.1 | -0.084381166 | 0.077042 | -1.09527 | 0.273399 | 0.605848 |
| RP11-417J8.3 | -0.144095463 | 0.131629 | -1.09471 | 0.273643 | 0.605848 |
| AC011754.1 | 0.140955762 | 0.129091 | 1.091909 | 0.274873 | 0.606997 |
| CROCCP2 | -0.110779441 | 0.101513 | -1.09128 | 0.27515 | 0.606997 |
| CTD-2560K21.6 | 0.115994337 | 0.106423 | 1.089938 | 0.27574 | 0.606997 |
| RP1-178F10.3 | 0.064493777 | 0.059169 | 1.089999 | 0.275714 | 0.606997 |
| RP11-658F2.8 | 0.073701998 | 0.067557 | 1.090955 | 0.275293 | 0.606997 |
| RP11-933H2.4 | 0.102272682 | 0.093586 | 1.092819 | 0.274473 | 0.606997 |
| RP11-214K3.18 | -0.110696242 | 0.101681 | -1.08866 | 0.276303 | 0.60704 |
| RP5-1180C10.2 | 0.074507626 | 0.068407 | 1.089173 | 0.276077 | 0.60704 |
| RSBN1L-AS1 | -0.085770257 | 0.078825 | -1.0881 | 0.276549 | 0.60704 |
| LINC00607 | 0.133258115 | 0.122625 | 1.086714 | 0.277163 | 0.60781 |
| RP11-21A7A.2 | 0.087616468 | 0.080749 | 1.085041 | 0.277904 | 0.608277 |
| RP11-326I11.4 | 0.130090306 | 0.119863 | 1.085323 | 0.277779 | 0.608277 |
| JAG1 | -0.114681222 | 0.105865 | -1.08327 | 0.278687 | 0.608808 |
| RP11-111M22.5 | 0.120115464 | 0.111062 | 1.08152 | 0.279466 | 0.608808 |
| RP11-214K3.21 | 0.105069559 | 0.097114 | 1.081923 | 0.279287 | 0.608808 |
| RP11-389C8.3 | 0.115145895 | 0.106422 | 1.081974 | 0.279264 | 0.608808 |
| RP11-697N18.4 | -0.101052535 | 0.093257 | -1.08359 | 0.278546 | 0.608808 |
| FAM215B | -0.11973401 | 0.111086 | -1.07785 | 0.281103 | 0.611796 |
| CTD-2541M15.1 | 0.09239593 | 0.086023 | 1.074083 | 0.282786 | 0.613583 |
| LL0XNC01-237H1.2 | 0.05940364 | 0.05536 | 1.073039 | 0.283254 | 0.613583 |
| RNF144A-AS1 | 0.12002448 | 0.111841 | 1.073175 | 0.283193 | 0.613583 |
| RP1-251M9.2 | 0.132712841 | 0.123423 | 1.07527 | 0.282254 | 0.613583 |
| RP11-439E19.1 | -0.093005947 | 0.086573 | -1.07431 | 0.282684 | 0.613583 |
| CTD-3025N20.3 | -0.108656675 | 0.101441 | -1.07113 | 0.28411 | 0.61486 |
| RP11-227G15.8 | 0.100283141 | 0.094031 | 1.066488 | 0.286203 | 0.618231 |
| RP11-361L15.4 | 0.086693998 | 0.08125 | 1.067003 | 0.28597 | 0.618231 |
| AC087393.1 | -0.105387606 | 0.09903 | -1.0642 | 0.28724 | 0.619168 |
| LINC00672 | 0.088214481 | 0.082928 | 1.06375 | 0.287442 | 0.619168 |
| LINC00856 | 0.082612604 | 0.077644 | 1.063992 | 0.287332 | 0.619168 |
| AC006538.1 | -0.112771432 | 0.106079 | -1.06309 | 0.287741 | 0.619233 |
| RP11-539I5.1 | 0.099711694 | 0.093862 | 1.062318 | 0.288091 | 0.619409 |
| RP11-38P22.2 | -0.142880648 | 0.134759 | -1.06026 | 0.289024 | 0.620837 |
| RP11-403I13.7 | -0.103516524 | 0.097733 | -1.05917 | 0.289522 | 0.621327 |
| RP11-549B18.1 | 0.090255465 | 0.085451 | 1.056228 | 0.290864 | 0.623628 |
| AC066593.1 | 0.098744972 | 0.09361 | 1.054854 | 0.291492 | 0.623814 |
| Y_RNA | 0.077698744 | 0.073633 | 1.055219 | 0.291325 | 0.623814 |
| KB-1460A1.5 | -0.080563005 | 0.076446 | -1.05385 | 0.291952 | 0.62422 |
| LINC00535 | 0.081485529 | 0.07741 | 1.052646 | 0.292503 | 0.624241 |
| RP11-573D15.2 | 0.081878862 | 0.077754 | 1.053055 | 0.292316 | 0.624241 |
| LINC00327 | 0.09702791 | 0.092321 | 1.050979 | 0.293268 | 0.62493 |
| LINC00909 | -0.066205213 | 0.063007 | -1.05076 | 0.293368 | 0.62493 |
| AC005162.5 | 0.127275119 | 0.121618 | 1.046512 | 0.295325 | 0.625556 |
| AC012065.7 | 0.137542312 | 0.131145 | 1.048779 | 0.29428 | 0.625556 |
| AC093673.5 | 0.117161156 | 0.111926 | 1.046772 | 0.295205 | 0.625556 |
| LA16c-313D11.12 | -0.082861972 | 0.079163 | -1.04672 | 0.295228 | 0.625556 |
| LINC00617 | 0.124284826 | 0.119002 | 1.044393 | 0.296304 | 0.625556 |
| LOXL1-AS1 | 0.11876709 | 0.113679 | 1.044756 | 0.296136 | 0.625556 |
| RP11-258F22.1 | 0.094971733 | 0.090948 | 1.044242 | 0.296373 | 0.625556 |
| RP11-561O23.8 | 0.118129044 | 0.11306 | 1.044832 | 0.296101 | 0.625556 |
| RP11-731J8.2 | -0.097464563 | 0.093194 | -1.04582 | 0.295642 | 0.625556 |
| UHRF1 | -0.121826478 | 0.116568 | -1.04511 | 0.295972 | 0.625556 |
| RP11-626K17.3 | -0.104835909 | 0.100451 | -1.04365 | 0.296645 | 0.625558 |
| AC005786.7 | 0.117487073 | 0.112857 | 1.041025 | 0.297864 | 0.626981 |
| RP11-114F10.2 | 0.125324192 | 0.120356 | 1.041279 | 0.297746 | 0.626981 |
| AP001347.6 | 0.101961735 | 0.098069 | 1.039699 | 0.29848 | 0.627706 |
| AC129929.5 | 0.106627776 | 0.10278 | 1.037437 | 0.299532 | 0.628773 |
| GS1-124K5.11 | 0.07686119 | 0.074071 | 1.037669 | 0.299424 | 0.628773 |
| FENDRR | 0.1261923 | 0.121778 | 1.036247 | 0.300087 | 0.629364 |
| RP11-562L8.1 | 0.109757438 | 0.105982 | 1.035622 | 0.300379 | 0.629404 |
| RP11-17A4.2 | 0.082578396 | 0.079793 | 1.034903 | 0.300714 | 0.629535 |
| RP11-191L9.4 | -0.139038814 | 0.134609 | -1.03291 | 0.301648 | 0.630918 |
| AC093627.10 | 0.10897109 | 0.105755 | 1.030407 | 0.302819 | 0.631078 |
| RP1-8B1.4 | 0.112091311 | 0.108739 | 1.030829 | 0.302621 | 0.631078 |
| RP11-170N16.3 | 0.098207268 | 0.0952 | 1.031584 | 0.302267 | 0.631078 |
| RP11-344A5.1 | -0.127646068 | 0.123856 | -1.0306 | 0.302727 | 0.631078 |
| RP11-423P10.2 | -0.082074483 | 0.079928 | -1.02685 | 0.304492 | 0.633991 |
| AC018766.4 | -0.113893337 | 0.111093 | -1.02521 | 0.305265 | 0.634456 |
| XX-CR54.3 | 0.113656709 | 0.11086 | 1.025227 | 0.305256 | 0.634456 |
| CYP1B1-AS1 | 0.099292946 | 0.096964 | 1.024019 | 0.305826 | 0.63478 |
| FAM41C | -0.113355385 | 0.11073 | -1.02371 | 0.305971 | 0.63478 |
| RP11-532F6.5 | 0.111092815 | 0.108923 | 1.019925 | 0.307764 | 0.637926 |
| CTB-25B13.6 | -0.091500782 | 0.089787 | -1.01909 | 0.308162 | 0.638178 |
| RP11-468I15.1 | 0.121674312 | 0.119703 | 1.016465 | 0.309408 | 0.640183 |
| CTC-321K16.1 | -0.117544859 | 0.11589 | -1.01428 | 0.310451 | 0.640197 |
| LINC00222 | -0.113010382 | 0.111263 | -1.01571 | 0.30977 | 0.640197 |
| RP11-378J18.8 | -0.106393283 | 0.104881 | -1.01441 | 0.310385 | 0.640197 |
| RP11-384O8.1 | 0.093539953 | 0.092237 | 1.014122 | 0.310525 | 0.640197 |
| AC016747.3 | -0.082198821 | 0.081251 | -1.01167 | 0.311697 | 0.642039 |
| RP11-276H19.1 | -0.102155275 | 0.101048 | -1.01096 | 0.312037 | 0.642168 |
| RP11-594N15.3 | -0.09300802 | 0.09217 | -1.00909 | 0.312932 | 0.643353 |
| RP5-855D21.3 | -0.102577006 | 0.101703 | -1.00859 | 0.313171 | 0.643353 |
| RP11-477H21.2 | -0.106291285 | 0.105571 | -1.00682 | 0.314022 | 0.644527 |
| RP11-182J1.12 | 0.093958871 | 0.093755 | 1.002176 | 0.316259 | 0.648314 |
| RP11-408A13.4 | -0.087890313 | 0.08773 | -1.00182 | 0.316429 | 0.648314 |
| RP11-159D12.2 | -0.06783935 | 0.067854 | -0.99979 | 0.317413 | 0.649754 |
| RP11-874J12.3 | 0.110260315 | 0.110395 | 0.998779 | 0.317902 | 0.650177 |
| LINC00158 | 0.079455914 | 0.079647 | 0.997599 | 0.318474 | 0.650305 |
| LINC00683 | -0.115072106 | 0.115362 | -0.99749 | 0.318528 | 0.650305 |
| RP11-968O1.5 | -0.121020571 | 0.121472 | -0.99628 | 0.319114 | 0.650925 |
| AC005592.1 | 0.108065707 | 0.109168 | 0.989902 | 0.322222 | 0.651777 |
| AC011196.3 | 0.111693625 | 0.11259 | 0.992038 | 0.321179 | 0.651777 |
| CTD-3092A11.2 | -0.078860973 | 0.079649 | -0.99011 | 0.322121 | 0.651777 |
| GMDS-AS1 | -0.055136321 | 0.055695 | -0.98996 | 0.322193 | 0.651777 |
| NCBP2-AS2 | 0.093141195 | 0.094082 | 0.990001 | 0.322174 | 0.651777 |
| PRKCQ-AS1 | -0.128755577 | 0.130125 | -0.98948 | 0.32243 | 0.651777 |
| RP1-111D6.3 | -0.135221971 | 0.136794 | -0.98851 | 0.322903 | 0.651777 |
| RP11-106M7.1 | -0.114831064 | 0.115667 | -0.99277 | 0.320823 | 0.651777 |
| RP11-277L2.5 | -0.110091434 | 0.111376 | -0.98847 | 0.322922 | 0.651777 |
| RP11-488C13.7 | -0.084742019 | 0.085322 | -0.9932 | 0.320612 | 0.651777 |
| RP5-1119A7.17 | 0.095179422 | 0.096266 | 0.988714 | 0.322803 | 0.651777 |
| TTN-AS1 | -0.080426047 | 0.081078 | -0.99196 | 0.321219 | 0.651777 |
| EPHA1-AS1 | 0.125203895 | 0.127281 | 0.983683 | 0.325271 | 0.652264 |
| FMR1-IT1 | -0.098431728 | 0.099746 | -0.98683 | 0.323727 | 0.652264 |
| OPCML-IT2 | 0.102260951 | 0.103967 | 0.983591 | 0.325317 | 0.652264 |
| RP11-111M22.4 | 0.100257959 | 0.101953 | 0.98337 | 0.325425 | 0.652264 |
| RP11-692D12.1 | -0.095930168 | 0.097285 | -0.98608 | 0.324096 | 0.652264 |
| RP11-805I24.1 | -0.087623971 | 0.088921 | -0.98542 | 0.324419 | 0.652264 |
| RP4-809F18.2 | 0.117265265 | 0.11911 | 0.984511 | 0.324865 | 0.652264 |
| UBXN8 | -0.060401092 | 0.061251 | -0.98613 | 0.32407 | 0.652264 |
| CRYM-AS1 | 0.096648953 | 0.098525 | 0.98096 | 0.326612 | 0.652941 |
| RP11-686G23.2 | -0.110984198 | 0.11313 | -0.98103 | 0.326576 | 0.652941 |
| RP11-752G15.6 | -0.100621856 | 0.102488 | -0.98179 | 0.326204 | 0.652941 |
| DLX6-AS1 | -0.109968613 | 0.112222 | -0.97992 | 0.327125 | 0.653401 |
| RP11-378I6.1 | 0.126815039 | 0.129806 | 0.97696 | 0.328589 | 0.655757 |
| RP4-610C12.4 | 0.1269331 | 0.130008 | 0.976345 | 0.328894 | 0.655797 |
| AC013402.2 | -0.082163444 | 0.084233 | -0.97543 | 0.329349 | 0.656138 |
| AC005789.11 | 0.086897654 | 0.089378 | 0.97225 | 0.330926 | 0.658554 |
| RP11-309G3.3 | -0.114410436 | 0.117765 | -0.97152 | 0.33129 | 0.658554 |
| RP11-722E23.2 | -0.121014725 | 0.124595 | -0.97126 | 0.331418 | 0.658554 |
| AC087294.2 | 0.089304517 | 0.092167 | 0.968945 | 0.332572 | 0.660279 |
| AC113189.5 | -0.092344879 | 0.095487 | -0.96709 | 0.333498 | 0.661038 |
| RP11-359J14.2 | -0.083199948 | 0.086105 | -0.96626 | 0.333914 | 0.661038 |
| RP11-373L24.1 | 0.045055412 | 0.046655 | 0.965705 | 0.334192 | 0.661038 |
| RP11-98D18.9 | 0.077842688 | 0.080562 | 0.966245 | 0.333922 | 0.661038 |
| RP4-665J23.1 | 0.101902312 | 0.105564 | 0.965314 | 0.334387 | 0.661038 |
| LINC00693 | -0.08137535 | 0.084415 | -0.96399 | 0.33505 | 0.661782 |
| BZRAP1-AS1 | 0.083571585 | 0.086807 | 0.962724 | 0.335686 | 0.662471 |
| AC002429.5 | -0.121533442 | 0.126446 | -0.96115 | 0.336478 | 0.66329 |
| ALDH1L1-AS2 | -0.105689558 | 0.110088 | -0.96005 | 0.337031 | 0.66329 |
| RP11-215H22.1 | 0.103193454 | 0.10761 | 0.958954 | 0.337582 | 0.66329 |
| RP11-406A9.2 | -0.119432859 | 0.124494 | -0.95935 | 0.337384 | 0.66329 |
| RP11-556N21.1 | -0.116924557 | 0.122115 | -0.95749 | 0.338318 | 0.66329 |
| RP11-690G19.3 | 0.064000961 | 0.066674 | 0.959908 | 0.337101 | 0.66329 |
| RP11-761E20.1 | 0.081543231 | 0.085094 | 0.958276 | 0.337924 | 0.66329 |
| SNORA76 | -0.107683611 | 0.112484 | -0.95733 | 0.338402 | 0.66329 |
| RP11-87N24.2 | 0.103599677 | 0.108429 | 0.955464 | 0.339343 | 0.664571 |
| AC068282.3 | 0.097247954 | 0.102141 | 0.952094 | 0.341049 | 0.665239 |
| RP11-10O22.1 | -0.098741123 | 0.103749 | -0.95173 | 0.341233 | 0.665239 |
| RP11-20D14.6 | -0.088110028 | 0.092395 | -0.95363 | 0.340273 | 0.665239 |
| RP11-660L16.2 | 0.100466579 | 0.105465 | 0.952608 | 0.340789 | 0.665239 |
| RP11-739P1.2 | -0.083678477 | 0.087745 | -0.95366 | 0.340258 | 0.665239 |
| RP11-814P5.1 | -0.094213067 | 0.099028 | -0.95137 | 0.341414 | 0.665239 |
| AC017116.11 | 0.079002193 | 0.083246 | 0.949024 | 0.342609 | 0.66644 |
| LINC00614 | 0.115122801 | 0.121247 | 0.949492 | 0.342371 | 0.66644 |
| RP11-337C18.8 | -0.073557033 | 0.077776 | -0.94575 | 0.344276 | 0.66912 |
| CTC-338M12.4 | 0.077114362 | 0.081662 | 0.944307 | 0.345013 | 0.669424 |
| HCCAT3 | 0.103890887 | 0.109978 | 0.944654 | 0.344835 | 0.669424 |
| LINC00511 | -0.059638427 | 0.063259 | -0.94277 | 0.345801 | 0.669771 |
| RP11-282K24.3 | -0.091344883 | 0.096943 | -0.94225 | 0.346063 | 0.669771 |
| RP11-351M8.2 | -0.097144979 | 0.103065 | -0.94256 | 0.345908 | 0.669771 |
| AC011747.4 | 0.050690668 | 0.053903 | 0.940412 | 0.347006 | 0.670554 |
| TMEM254-AS1 | -0.074359104 | 0.079078 | -0.94033 | 0.347049 | 0.670554 |
| LINC01006 | 0.084638746 | 0.09017 | 0.938654 | 0.347908 | 0.670695 |
| RP11-34P13.7 | 0.090160124 | 0.09607 | 0.938488 | 0.347994 | 0.670695 |
| WDR7-UT1 | 0.081135367 | 0.086384 | 0.939245 | 0.347605 | 0.670695 |
| TTTY10 | 0.067980353 | 0.072485 | 0.937854 | 0.348319 | 0.670762 |
| AC005614.5 | 0.047679706 | 0.050897 | 0.936796 | 0.348863 | 0.670817 |
| AC016907.3 | -0.090661917 | 0.096941 | -0.93523 | 0.349672 | 0.670817 |
| RP11-134K1.3 | -0.09768815 | 0.104608 | -0.93385 | 0.350384 | 0.670817 |
| RP11-13J10.1 | -0.085114992 | 0.091102 | -0.93428 | 0.35016 | 0.670817 |
| RP11-290F24.6 | -0.080808212 | 0.086431 | -0.93494 | 0.349818 | 0.670817 |
| RP11-785H5.2 | -0.079667824 | 0.085269 | -0.93432 | 0.350141 | 0.670817 |
| XXbac-B444P24.13 | -0.088999046 | 0.095153 | -0.93532 | 0.349623 | 0.670817 |
| RP11-266K4.9 | 0.093721885 | 0.100507 | 0.93249 | 0.351083 | 0.671043 |
| RP11-51J9.6 | -0.094878461 | 0.101746 | -0.9325 | 0.351077 | 0.671043 |
| RP11-10C8.2 | -0.113430321 | 0.121721 | -0.93189 | 0.351394 | 0.671081 |
| RP11-388C12.8 | -0.074464013 | 0.080004 | -0.93075 | 0.351981 | 0.671647 |
| RP4-569M23.4 | -0.096986036 | 0.10438 | -0.92916 | 0.352807 | 0.672665 |
| RP11-167N5.5 | 0.08800241 | 0.094843 | 0.927872 | 0.353474 | 0.673381 |
| AL450992.2 | 0.110638159 | 0.120302 | 0.919672 | 0.357744 | 0.677599 |
| CTD-2228K2.7 | 0.093605827 | 0.101742 | 0.920033 | 0.357555 | 0.677599 |
| CTD-2336O2.1 | -0.082844147 | 0.089912 | -0.9214 | 0.356844 | 0.677599 |
| DGCR10 | 0.111247606 | 0.120864 | 0.920434 | 0.357346 | 0.677599 |
| RP11-140I16.3 | -0.090432719 | 0.09825 | -0.92043 | 0.357346 | 0.677599 |
| RP11-346D14.1 | 0.065041834 | 0.070524 | 0.922264 | 0.356391 | 0.677599 |
| RP11-398K22.12 | 0.049279169 | 0.053546 | 0.920312 | 0.35741 | 0.677599 |
| RP11-83A24.2 | -0.055385222 | 0.060294 | -0.91858 | 0.358314 | 0.678122 |
| AC096772.6 | 0.068354437 | 0.074921 | 0.912359 | 0.36158 | 0.678183 |
| CTB-113D17.1 | 0.106269108 | 0.116347 | 0.91338 | 0.361043 | 0.678183 |
| CTD-2240H23.2 | -0.11358937 | 0.124496 | -0.91239 | 0.361561 | 0.678183 |
| CTD-2540B15.13 | 0.09222008 | 0.100607 | 0.916639 | 0.359332 | 0.678183 |
| LAMTOR5-AS1 | -0.082101022 | 0.089669 | -0.9156 | 0.359876 | 0.678183 |
| RNU11 | 0.120014521 | 0.131234 | 0.914509 | 0.36045 | 0.678183 |
| RP1-86D1.3 | -0.085159496 | 0.093201 | -0.91372 | 0.360866 | 0.678183 |
| RP1-90J20.12 | 0.086442343 | 0.094443 | 0.915284 | 0.360043 | 0.678183 |
| RP11-5L12.1 | -0.093707795 | 0.102476 | -0.91444 | 0.360486 | 0.678183 |
| RP11-762I7.4 | -0.0854782 | 0.093151 | -0.91763 | 0.358812 | 0.678183 |
| SNHG16 | 0.069185256 | 0.075817 | 0.912525 | 0.361493 | 0.678183 |
| DANCR | -0.066036776 | 0.072516 | -0.91065 | 0.36248 | 0.678767 |
| RP11-216L13.18 | 0.072599639 | 0.079696 | 0.910955 | 0.362319 | 0.678767 |
| RP11-497D6.5 | -0.113571734 | 0.124908 | -0.90924 | 0.363223 | 0.679607 |
| CTD-2545H1.1 | 0.103467345 | 0.113919 | 0.908251 | 0.363746 | 0.680033 |
| RP11-363E6.4 | -0.088297959 | 0.097302 | -0.90747 | 0.36416 | 0.680257 |
| AQP4-AS1 | -0.095579739 | 0.105733 | -0.90397 | 0.366012 | 0.681 |
| CTD-3199J23.4 | -0.079237449 | 0.087605 | -0.90448 | 0.36574 | 0.681 |
| DSCAM-IT1 | 0.094406415 | 0.104349 | 0.90472 | 0.365614 | 0.681 |
| RP11-1246C19.1 | 0.075378628 | 0.08339 | 0.903928 | 0.366034 | 0.681 |
| RP11-490G2.2 | 0.10320115 | 0.113981 | 0.905422 | 0.365242 | 0.681 |
| RP11-480C16.1 | 0.094672149 | 0.104848 | 0.902945 | 0.366555 | 0.68142 |
| LINC00882 | 0.051300352 | 0.056986 | 0.900231 | 0.367997 | 0.681903 |
| MFI2-AS1 | 0.069615975 | 0.077288 | 0.900733 | 0.36773 | 0.681903 |
| RP11-141M3.5 | 0.090124545 | 0.099928 | 0.901897 | 0.367112 | 0.681903 |
| RP11-710C12.1 | -0.078358151 | 0.086975 | -0.90093 | 0.367628 | 0.681903 |
| SCARNA10 | -0.085523285 | 0.0953 | -0.89741 | 0.369499 | 0.684136 |
| CTD-2554C21.2 | 0.075457007 | 0.08431 | 0.894995 | 0.37079 | 0.685025 |
| RP1-184J9.2 | 0.078487272 | 0.087779 | 0.894149 | 0.371242 | 0.685025 |
| RP11-165E7.1 | -0.096593483 | 0.10809 | -0.89364 | 0.371517 | 0.685025 |
| RP11-206L10.10 | 0.105277908 | 0.117869 | 0.89318 | 0.371761 | 0.685025 |
| RP11-53O19.1 | 0.066539443 | 0.074324 | 0.895259 | 0.370649 | 0.685025 |
| U91328.19 | 0.067145874 | 0.075174 | 0.893203 | 0.371748 | 0.685025 |
| RP11-179A16.1 | -0.115185834 | 0.129167 | -0.89176 | 0.372524 | 0.685186 |
| RP11-24B19.3 | -0.074611214 | 0.083619 | -0.89228 | 0.372245 | 0.685186 |
| RP11-96K19.4 | -0.092573059 | 0.103857 | -0.89135 | 0.372739 | 0.685186 |
| RP11-351I21.11 | 0.084424091 | 0.094846 | 0.890121 | 0.373401 | 0.685857 |
| RP1-240B8.3 | 0.078348855 | 0.08831 | 0.887198 | 0.374973 | 0.687102 |
| RP11-441O15.3 | -0.065580023 | 0.073851 | -0.88801 | 0.374537 | 0.687102 |
| RP5-1021I20.5 | 0.059715796 | 0.067287 | 0.887473 | 0.374824 | 0.687102 |
| BSN-AS2 | 0.0765761 | 0.086562 | 0.884637 | 0.376352 | 0.687757 |
| RP11-158K1.3 | -0.072355826 | 0.081806 | -0.88448 | 0.376437 | 0.687757 |
| RP11-214K3.23 | 0.083979259 | 0.094965 | 0.884322 | 0.376523 | 0.687757 |
| RP5-1172N10.4 | -0.065774878 | 0.074338 | -0.88481 | 0.376259 | 0.687757 |
| LINC00641 | -0.043691012 | 0.049629 | -0.88035 | 0.37867 | 0.690616 |
| RP11-434B12.1 | 0.088289929 | 0.100293 | 0.88032 | 0.378686 | 0.690616 |
| RP1-283E3.8 | 0.06598599 | 0.075046 | 0.879273 | 0.379253 | 0.691103 |
| AC011288.2 | 0.103058285 | 0.117523 | 0.876918 | 0.380531 | 0.69274 |
| SNAP25-AS1 | 0.066304132 | 0.075645 | 0.876512 | 0.380752 | 0.69274 |
| AC115115.2 | -0.051001416 | 0.058408 | -0.87319 | 0.382562 | 0.69422 |
| KMT2B | 0.059367352 | 0.068102 | 0.871743 | 0.383348 | 0.69422 |
| RP11-325L7.1 | 0.088985642 | 0.101987 | 0.872523 | 0.382923 | 0.69422 |
| RP11-622K12.1 | 0.061594961 | 0.070471 | 0.874044 | 0.382094 | 0.69422 |
| RP11-807H7.1 | 0.099907536 | 0.114607 | 0.871739 | 0.383351 | 0.69422 |
| ZNF205-AS1 | 0.084585803 | 0.097035 | 0.871703 | 0.38337 | 0.69422 |
| RP11-529E10.6 | 0.07608822 | 0.087444 | 0.87014 | 0.384224 | 0.695219 |
| CTC-503J8.6 | 0.073163541 | 0.084192 | 0.869008 | 0.384843 | 0.695249 |
| RP11-1E4.1 | -0.079643678 | 0.091605 | -0.86943 | 0.384614 | 0.695249 |
| AL132709.5 | 0.059487892 | 0.068617 | 0.866957 | 0.385966 | 0.696561 |
| NAMA | 0.085703703 | 0.098899 | 0.866578 | 0.386173 | 0.696561 |
| RP11-275H4.1 | -0.116035391 | 0.133992 | -0.86599 | 0.386497 | 0.696601 |
| AC011995.1 | 0.090884226 | 0.105195 | 0.863962 | 0.387609 | 0.698059 |
| DICER1-AS1 | -0.058747475 | 0.068075 | -0.86298 | 0.388147 | 0.698482 |
| CTC-550B14.6 | 0.06916132 | 0.0804 | 0.860215 | 0.389671 | 0.698707 |
| CTD-3220F14.1 | 0.1133248 | 0.131663 | 0.860721 | 0.389392 | 0.698707 |
| LINC00599 | 0.069690998 | 0.081035 | 0.860006 | 0.389786 | 0.698707 |
| LINC00649 | -0.06697314 | 0.077678 | -0.86219 | 0.388583 | 0.698707 |
| WEE2-AS1 | -0.101924558 | 0.118498 | -0.86013 | 0.389715 | 0.698707 |
| RP11-266L9.5 | 0.071291707 | 0.083039 | 0.858534 | 0.390597 | 0.699618 |
| RP11-348F1.2 | 0.085041235 | 0.099184 | 0.857411 | 0.391218 | 0.700186 |
| RP11-307B6.3 | -0.100106098 | 0.117269 | -0.85365 | 0.3933 | 0.703368 |
| CTD-3234P18.2 | -0.066235641 | 0.077798 | -0.85138 | 0.394559 | 0.704526 |
| FAM66C | -0.048494977 | 0.056925 | -0.85191 | 0.394263 | 0.704526 |
| RP1-168L15.5 | 0.077811151 | 0.091526 | 0.850154 | 0.39524 | 0.705196 |
| AC093843.1 | 0.08569617 | 0.101121 | 0.847463 | 0.396737 | 0.7056 |
| BX322557.10 | 0.088789688 | 0.104886 | 0.846533 | 0.397255 | 0.7056 |
| CTD-2630F21.1 | 0.072981721 | 0.086221 | 0.846451 | 0.397301 | 0.7056 |
| CTD-3064H18.4 | 0.113089921 | 0.133229 | 0.848836 | 0.395973 | 0.7056 |
| RP11-97O12.7 | -0.059517437 | 0.070221 | -0.84757 | 0.396678 | 0.7056 |
| RP5-921G16.1 | -0.078982102 | 0.093143 | -0.84796 | 0.396458 | 0.7056 |
| DGCR11 | -0.067300218 | 0.079614 | -0.84533 | 0.397927 | 0.706167 |
| CTC-479C5.10 | 0.054831268 | 0.065165 | 0.841416 | 0.400115 | 0.706788 |
| ERI3-IT1 | -0.07455743 | 0.088596 | -0.84155 | 0.400042 | 0.706788 |
| RP11-255A11.21 | 0.111235168 | 0.132039 | 0.842439 | 0.399542 | 0.706788 |
| RP11-305O6.3 | -0.089829704 | 0.106699 | -0.8419 | 0.399845 | 0.706788 |
| RP11-571M6.17 | 0.073957779 | 0.087817 | 0.842181 | 0.399687 | 0.706788 |
| SNHG12 | -0.06772579 | 0.080294 | -0.84347 | 0.398967 | 0.706788 |
| CTC-304I17.3 | 0.108340156 | 0.128999 | 0.839855 | 0.40099 | 0.70725 |
| RP1-101K10.6 | -0.083094614 | 0.098914 | -0.84007 | 0.40087 | 0.70725 |
| CTD-2196E14.9 | 0.052119841 | 0.062425 | 0.834914 | 0.403766 | 0.708661 |
| MAGI1-IT1 | -0.080044638 | 0.095632 | -0.83701 | 0.402588 | 0.708661 |
| NR2F1-AS1 | 0.053004492 | 0.063408 | 0.835928 | 0.403195 | 0.708661 |
| RP11-166D19.1 | -0.062034488 | 0.074425 | -0.83351 | 0.404554 | 0.708661 |
| RP11-30K9.6 | -0.080929564 | 0.09707 | -0.83372 | 0.404437 | 0.708661 |
| RP11-526I2.5 | 0.112061378 | 0.134267 | 0.834616 | 0.403934 | 0.708661 |
| RP11-977G19.5 | -0.06006715 | 0.071838 | -0.83615 | 0.403072 | 0.708661 |
| RP5-1061H20.4 | 0.075754577 | 0.09083 | 0.834026 | 0.404266 | 0.708661 |
| STARD13-IT1 | -0.088053194 | 0.10549 | -0.83471 | 0.403881 | 0.708661 |
| CTD-2538G9.5 | 0.082964232 | 0.099691 | 0.832211 | 0.40529 | 0.709411 |
| PRC1-AS1 | 0.07249724 | 0.087391 | 0.82957 | 0.406782 | 0.710944 |
| RP11-540B6.6 | -0.062929965 | 0.075839 | -0.82978 | 0.406661 | 0.710944 |
| CTA-217C2.2 | -0.08007398 | 0.096648 | -0.82851 | 0.407381 | 0.711302 |
| RP11-383C5.4 | -0.063415713 | 0.076578 | -0.82812 | 0.407603 | 0.711302 |
| HCG18 | 0.046272862 | 0.056225 | 0.822992 | 0.410513 | 0.712538 |
| LINC00839 | 0.096076761 | 0.116391 | 0.825466 | 0.409107 | 0.712538 |
| OSER1-AS1 | -0.054158729 | 0.06573 | -0.82396 | 0.409961 | 0.712538 |
| RP11-149P24.1 | 0.103338772 | 0.125465 | 0.823643 | 0.410142 | 0.712538 |
| RP11-151N17.1 | -0.077440874 | 0.09402 | -0.82366 | 0.410132 | 0.712538 |
| RP11-206L10.9 | 0.050231003 | 0.06107 | 0.822517 | 0.410782 | 0.712538 |
| RP11-2C24.4 | 0.059633102 | 0.072471 | 0.822853 | 0.410591 | 0.712538 |
| SOCS2-AS1 | 0.075941393 | 0.091976 | 0.825666 | 0.408994 | 0.712538 |
| AC012370.2 | -0.090291081 | 0.109898 | -0.82159 | 0.411311 | 0.71259 |
| FTX | -0.042447928 | 0.051697 | -0.82109 | 0.411593 | 0.71259 |
| RP11-244H3.1 | 0.069151352 | 0.084294 | 0.820358 | 0.412012 | 0.71259 |
| RP11-361F15.2 | 0.069635564 | 0.084891 | 0.820295 | 0.412048 | 0.71259 |
| EIF3J-AS1 | 0.041930175 | 0.051229 | 0.818479 | 0.413084 | 0.712778 |
| RP11-98I9.4 | -0.062279413 | 0.076027 | -0.81917 | 0.412688 | 0.712778 |
| RP4-756H11.3 | 0.084435954 | 0.103104 | 0.818943 | 0.412819 | 0.712778 |
| AC112229.7 | 0.088852383 | 0.108752 | 0.81702 | 0.413917 | 0.713079 |
| CTD-3138B18.5 | 0.05792283 | 0.07086 | 0.817423 | 0.413687 | 0.713079 |
| DPH6-AS1 | -0.077855616 | 0.095433 | -0.81581 | 0.414607 | 0.713079 |
| RP11-363E7.4 | 0.08277533 | 0.101574 | 0.81493 | 0.415112 | 0.713079 |
| RP11-727F15.12 | 0.073636918 | 0.090335 | 0.81515 | 0.414987 | 0.713079 |
| SENP3-EIF4A1 | 0.055902389 | 0.068527 | 0.815772 | 0.414631 | 0.713079 |
| AC005592.2 | 0.076254144 | 0.094131 | 0.810088 | 0.41789 | 0.716056 |
| CEBPA-AS1 | -0.074120529 | 0.091588 | -0.80928 | 0.418355 | 0.716056 |
| POLR2J4 | -0.05622713 | 0.069445 | -0.80966 | 0.418134 | 0.716056 |
| RP11-379H18.1 | -0.052845321 | 0.065305 | -0.8092 | 0.418398 | 0.716056 |
| RP11-445O16.3 | -0.075252129 | 0.092897 | -0.81006 | 0.417907 | 0.716056 |
| RP11-782C8.1 | -0.105990316 | 0.131183 | -0.80796 | 0.419116 | 0.716753 |
| RP11-384L8.1 | 0.09258445 | 0.114742 | 0.806892 | 0.419729 | 0.71727 |
| AC016725.4 | 0.076037676 | 0.09439 | 0.805573 | 0.420489 | 0.717506 |
| RP11-304L19.3 | 0.075862663 | 0.09416 | 0.80568 | 0.420428 | 0.717506 |
| CKMT2-AS1 | -0.05827934 | 0.07271 | -0.80153 | 0.422822 | 0.717978 |
| CPEB2-AS1 | -0.074412806 | 0.092862 | -0.80132 | 0.422944 | 0.717978 |
| KIAA1984-AS1 | 0.071477591 | 0.08917 | 0.801584 | 0.422793 | 0.717978 |
| RP1-20N2.6 | 0.049210954 | 0.061278 | 0.803078 | 0.42193 | 0.717978 |
| RP11-234K24.3 | 0.067012995 | 0.083558 | 0.801996 | 0.422555 | 0.717978 |
| RP11-6N17.4 | 0.059996103 | 0.074733 | 0.802804 | 0.422088 | 0.717978 |
| RP11-96H19.1 | -0.084158759 | 0.10465 | -0.80419 | 0.421286 | 0.717978 |
| AC090587.2 | 0.066256505 | 0.082773 | 0.800464 | 0.423442 | 0.718295 |
| SNHG7 | -0.068145298 | 0.085208 | -0.79975 | 0.423854 | 0.718465 |
| LINC00941 | 0.096000202 | 0.120782 | 0.79482 | 0.426718 | 0.718751 |
| LINC01024 | -0.073476752 | 0.092214 | -0.79681 | 0.425562 | 0.718751 |
| MEF2C-AS1 | 0.053238525 | 0.066943 | 0.795282 | 0.42645 | 0.718751 |
| MYLK-AS1 | -0.071754876 | 0.090018 | -0.79712 | 0.425382 | 0.718751 |
| RP11-444D3.1 | -0.055982765 | 0.070451 | -0.79463 | 0.426827 | 0.718751 |
| RP11-732A19.2 | 0.083863811 | 0.105519 | 0.794777 | 0.426743 | 0.718751 |
| RP11-95D17.1 | 0.054987656 | 0.069091 | 0.795875 | 0.426105 | 0.718751 |
| SLC25A5-AS1 | -0.064430335 | 0.080906 | -0.79636 | 0.425823 | 0.718751 |
| XXbac-BPG299F13.17 | 0.05024274 | 0.063087 | 0.796404 | 0.425797 | 0.718751 |
| Z84812.4 | 0.076854169 | 0.096802 | 0.793928 | 0.427237 | 0.718918 |
| RP11-793H13.8 | 0.045856865 | 0.057968 | 0.791072 | 0.428902 | 0.721193 |
| AC093642.3 | 0.081118216 | 0.102803 | 0.789068 | 0.430072 | 0.721567 |
| AC144521.1 | 0.062940699 | 0.079894 | 0.787802 | 0.430813 | 0.721567 |
| AJ271736.10 | 0.072287004 | 0.09189 | 0.786673 | 0.431473 | 0.721567 |
| FAM182A | -0.08746967 | 0.111176 | -0.78677 | 0.431418 | 0.721567 |
| RP11-21B21.4 | 0.076366913 | 0.096951 | 0.787683 | 0.430882 | 0.721567 |
| RP11-69E11.4 | -0.056041177 | 0.071262 | -0.78641 | 0.431626 | 0.721567 |
| RP13-216E22.4 | -0.066037805 | 0.083805 | -0.788 | 0.430699 | 0.721567 |
| TMEM9B-AS1 | -0.094452602 | 0.119714 | -0.78898 | 0.430122 | 0.721567 |
| AC018799.1 | 0.096018872 | 0.12235 | 0.784788 | 0.432578 | 0.722192 |
| AD000090.2 | -0.053040171 | 0.067731 | -0.7831 | 0.433566 | 0.722192 |
| CTD-2006H14.2 | -0.068486882 | 0.087329 | -0.78424 | 0.4329 | 0.722192 |
| LINC00887 | 0.052831969 | 0.067416 | 0.783672 | 0.433233 | 0.722192 |
| SNHG11 | 0.052855878 | 0.067451 | 0.78362 | 0.433263 | 0.722192 |
| CTD-2574D22.2 | -0.069041001 | 0.088259 | -0.78226 | 0.434064 | 0.722351 |
| RP11-46C24.7 | -0.062780356 | 0.080295 | -0.78188 | 0.434287 | 0.722351 |
| AC067961.1 | 0.0811991 | 0.10459 | 0.776354 | 0.43754 | 0.723394 |
| LINC00471 | 0.080560375 | 0.10336 | 0.779418 | 0.435733 | 0.723394 |
| RP1-153G14.4 | -0.074351301 | 0.095624 | -0.77754 | 0.43684 | 0.723394 |
| RP11-18H7.1 | -0.056363 | 0.072661 | -0.7757 | 0.437928 | 0.723394 |
| RP11-279F6.1 | 0.05698413 | 0.073457 | 0.775751 | 0.437896 | 0.723394 |
| RP11-284N8.3 | -0.064408042 | 0.083055 | -0.77549 | 0.43805 | 0.723394 |
| RP11-486G15.2 | -0.072350476 | 0.093096 | -0.77716 | 0.437066 | 0.723394 |
| RP11-53B2.6 | -0.081793522 | 0.105246 | -0.77717 | 0.43706 | 0.723394 |
| RP11-676J15.1 | 0.093722306 | 0.120658 | 0.77676 | 0.4373 | 0.723394 |
| RP4-798A10.7 | -0.079930119 | 0.102782 | -0.77767 | 0.436766 | 0.723394 |
| AC004951.6 | 0.067082325 | 0.086933 | 0.771652 | 0.44032 | 0.725517 |
| AP000320.7 | 0.051372737 | 0.06663 | 0.77102 | 0.440695 | 0.725517 |
| LINC00390 | 0.103740992 | 0.134459 | 0.771543 | 0.440385 | 0.725517 |
| RBFADN | 0.065198206 | 0.084447 | 0.772065 | 0.440076 | 0.725517 |
| RP11-767N6.7 | -0.06999186 | 0.090821 | -0.77066 | 0.440908 | 0.725517 |
| PVT1 | 0.067677935 | 0.087907 | 0.769883 | 0.441369 | 0.725758 |
| RP11-305L7.7 | -0.062060851 | 0.080779 | -0.76828 | 0.442319 | 0.726285 |
| TMEM44-AS1 | -0.085648231 | 0.111406 | -0.76879 | 0.442016 | 0.726285 |
| CTD-2377O17.1 | 0.066379397 | 0.086999 | 0.762988 | 0.445471 | 0.73094 |
| RP11-112J3.16 | 0.06472583 | 0.085014 | 0.761359 | 0.446443 | 0.731494 |
| RP11-400F19.6 | 0.053270792 | 0.069936 | 0.761712 | 0.446232 | 0.731494 |
| AC091133.1 | -0.065184482 | 0.085867 | -0.75913 | 0.447775 | 0.733156 |
| AC074363.1 | -0.065423316 | 0.08648 | -0.75651 | 0.449342 | 0.733415 |
| AGAP11 | -0.056927979 | 0.075083 | -0.7582 | 0.448333 | 0.733415 |
| RP11-110I1.12 | -0.061618189 | 0.081402 | -0.75696 | 0.449076 | 0.733415 |
| RP11-16N11.2 | -0.053035582 | 0.070098 | -0.75659 | 0.449294 | 0.733415 |
| RP11-2E11.9 | 0.08269574 | 0.109355 | 0.756211 | 0.449523 | 0.733415 |
| KCP | -0.068982069 | 0.091515 | -0.75378 | 0.450981 | 0.734415 |
| RP11-195F19.30 | 0.072350456 | 0.095989 | 0.753738 | 0.451007 | 0.734415 |
| RP11-690D19.3 | -0.049255583 | 0.065361 | -0.7536 | 0.45109 | 0.734415 |
| CBR3-AS1 | -0.055446954 | 0.073678 | -0.75256 | 0.451714 | 0.734652 |
| RP11-196E1.3 | 0.078522751 | 0.104377 | 0.752296 | 0.451873 | 0.734652 |
| TPT1-AS1 | -0.047858488 | 0.063735 | -0.7509 | 0.452714 | 0.735501 |
| LINC00925 | 0.040915811 | 0.054586 | 0.74956 | 0.45352 | 0.736095 |
| LINC00943 | -0.093687396 | 0.125133 | -0.7487 | 0.454037 | 0.736095 |
| RP11-567M16.1 | 0.101557813 | 0.135602 | 0.748942 | 0.453892 | 0.736095 |
| MIR663A | 0.081294933 | 0.108803 | 0.747173 | 0.454959 | 0.737072 |
| ERVK3-1 | 0.036900471 | 0.049437 | 0.746413 | 0.455418 | 0.737297 |
| RP11-220I1.1 | 0.034410715 | 0.046249 | 0.744038 | 0.456854 | 0.739087 |
| RP11-353B9.1 | 0.060932161 | 0.08195 | 0.743525 | 0.457164 | 0.739087 |
| KB-1517D11.4 | 0.082283903 | 0.110895 | 0.741999 | 0.458088 | 0.739633 |
| RP11-661A12.14 | -0.065806158 | 0.088698 | -0.74191 | 0.458143 | 0.739633 |
| STX18-AS1 | 0.040216613 | 0.05433 | 0.740224 | 0.459164 | 0.740763 |
| HCP5 | -0.077362098 | 0.104644 | -0.73929 | 0.459733 | 0.741162 |
| LINC00863 | -0.07499104 | 0.101867 | -0.73617 | 0.461628 | 0.743698 |
| MAP3K14 | 0.057204235 | 0.077774 | 0.735518 | 0.462024 | 0.743817 |
| AC113617.1 | -0.097562158 | 0.132924 | -0.73397 | 0.462966 | 0.744243 |
| RP1-265C24.8 | 0.077028294 | 0.105009 | 0.733537 | 0.463231 | 0.744243 |
| RP11-445H22.4 | 0.069483009 | 0.094729 | 0.733495 | 0.463257 | 0.744243 |
| RP11-618G20.1 | -0.058115459 | 0.079302 | -0.73284 | 0.463655 | 0.744366 |
| CTC-344H19.4 | 0.074437734 | 0.101708 | 0.731873 | 0.464246 | 0.744465 |
| RP5-1112D6.4 | -0.065809547 | 0.089943 | -0.73168 | 0.464363 | 0.744465 |
| AC092535.3 | 0.089670559 | 0.122991 | 0.729085 | 0.46595 | 0.745618 |
| HHIP-AS1 | -0.066199323 | 0.090884 | -0.72839 | 0.466375 | 0.745618 |
| RP11-398C13.6 | 0.068379297 | 0.09386 | 0.728521 | 0.466295 | 0.745618 |
| RP11-692C24.2 | 0.064377545 | 0.088351 | 0.728658 | 0.466211 | 0.745618 |
| CTC-498M16.2 | -0.090121078 | 0.124738 | -0.72248 | 0.469998 | 0.745748 |
| CTC-512J12.4 | -0.055332293 | 0.076549 | -0.72284 | 0.469781 | 0.745748 |
| CTD-3049M7.1 | 0.091812887 | 0.126346 | 0.726676 | 0.467424 | 0.745748 |
| CTD-3064H18.1 | -0.071054931 | 0.0981 | -0.72431 | 0.468875 | 0.745748 |
| MIR519A2 | -0.06553336 | 0.09066 | -0.72285 | 0.469772 | 0.745748 |
| RP11-1191J2.2 | 0.076140151 | 0.105087 | 0.724547 | 0.46873 | 0.745748 |
| RP11-397A16.1 | -0.071080631 | 0.09802 | -0.72516 | 0.468351 | 0.745748 |
| RP11-434P11.2 | -0.073894243 | 0.102282 | -0.72246 | 0.470012 | 0.745748 |
| RP5-1103G7.4 | 0.059870347 | 0.08275 | 0.72351 | 0.469367 | 0.745748 |
| SLX1A-SULT1A3 | -0.063196251 | 0.087165 | -0.72502 | 0.468438 | 0.745748 |
| Z83851.1 | 0.071864423 | 0.099165 | 0.724694 | 0.46864 | 0.745748 |
| C11orf95 | 0.060434406 | 0.083796 | 0.721211 | 0.47078 | 0.746452 |
| RP11-293M10.6 | 0.069348132 | 0.096318 | 0.719994 | 0.471529 | 0.747127 |
| AC144652.1 | 0.060530542 | 0.084217 | 0.718746 | 0.472298 | 0.747832 |
| CTD-2555O16.2 | -0.075323473 | 0.105059 | -0.71697 | 0.473395 | 0.749055 |
| SPPL2B | 0.052188656 | 0.072889 | 0.716004 | 0.473989 | 0.74948 |
| AC090044.1 | -0.082376027 | 0.11514 | -0.71544 | 0.474335 | 0.74951 |
| CAPN10-AS1 | 0.050364193 | 0.070447 | 0.714922 | 0.474657 | 0.74951 |
| LINC00667 | -0.054998397 | 0.077056 | -0.71374 | 0.475385 | 0.749633 |
| RP11-1000B6.3 | 0.068836841 | 0.096421 | 0.713919 | 0.475277 | 0.749633 |
| LINC00894 | -0.043191752 | 0.06069 | -0.71168 | 0.476662 | 0.751134 |
| LINC00173 | 0.068073706 | 0.095778 | 0.710743 | 0.477243 | 0.751536 |
| AC003665.1 | -0.058352183 | 0.082258 | -0.70938 | 0.478088 | 0.752286 |
| RP11-267N12.3 | -0.070742087 | 0.099788 | -0.70892 | 0.478372 | 0.752286 |
| RP11-580I1.2 | -0.088559674 | 0.125195 | -0.70738 | 0.479333 | 0.752772 |
| XXbac-BPG154L12.4 | 0.034045368 | 0.048125 | 0.707435 | 0.479296 | 0.752772 |
| RP11-104H15.10 | -0.085227792 | 0.120646 | -0.70643 | 0.479922 | 0.753184 |
| AF131215.9 | -0.05227649 | 0.074168 | -0.70484 | 0.480912 | 0.753685 |
| CTC-360G5.9 | -0.073028326 | 0.10358 | -0.70504 | 0.480782 | 0.753685 |
| FAM95B1 | 0.068894525 | 0.097814 | 0.704339 | 0.481222 | 0.753685 |
| LINC01007 | 0.092916159 | 0.132104 | 0.703357 | 0.481834 | 0.754132 |
| RP11-592B15.3 | 0.083478619 | 0.118906 | 0.702057 | 0.482644 | 0.754888 |
| RP11-1275H24.3 | 0.055638077 | 0.079405 | 0.70069 | 0.483497 | 0.755709 |
| LINC01013 | -0.067603635 | 0.097092 | -0.69629 | 0.48625 | 0.759498 |
| RP11-119F19.2 | 0.054023583 | 0.07798 | 0.692791 | 0.488441 | 0.762404 |
| RP11-690I21.2 | 0.051461346 | 0.074391 | 0.691769 | 0.489082 | 0.762889 |
| CTA-445C9.14 | -0.057610657 | 0.083406 | -0.69072 | 0.489739 | 0.763398 |
| AC092669.3 | 0.053621356 | 0.077751 | 0.689653 | 0.490412 | 0.763931 |
| RP11-138A9.1 | 0.062916875 | 0.091538 | 0.687334 | 0.491872 | 0.765659 |
| RP11-473M20.14 | 0.035600565 | 0.051843 | 0.686703 | 0.49227 | 0.765659 |
| RP11-77H9.2 | 0.05791467 | 0.084386 | 0.68631 | 0.492517 | 0.765659 |
| RMST | -0.06940855 | 0.101277 | -0.68533 | 0.493135 | 0.766103 |
| LINC00982 | -0.073809561 | 0.107909 | -0.684 | 0.493976 | 0.76626 |
| RP11-255P5.2 | 0.076715688 | 0.112269 | 0.683323 | 0.494403 | 0.76626 |
| RP11-383M4.6 | 0.07593966 | 0.111062 | 0.683758 | 0.494128 | 0.76626 |
| RP11-848P1.4 | 0.062666117 | 0.091742 | 0.683067 | 0.494565 | 0.76626 |
| AC004893.11 | -0.042066101 | 0.061658 | -0.68225 | 0.495079 | 0.766541 |
| RP11-342K6.1 | -0.046863556 | 0.068782 | -0.68134 | 0.495659 | 0.766926 |
| RP11-473I1.10 | -0.044412201 | 0.065273 | -0.68041 | 0.496245 | 0.767317 |
| RP11-552M11.4 | 0.039950287 | 0.058791 | 0.679533 | 0.4968 | 0.767661 |
| AC012309.5 | -0.079498067 | 0.117419 | -0.67704 | 0.498378 | 0.769584 |
| RP11-464D20.6 | -0.05874259 | 0.08693 | -0.67574 | 0.499204 | 0.770344 |
| CTD-3118D7.1 | 0.076065798 | 0.112696 | 0.674965 | 0.499698 | 0.77059 |
| ASMTL-AS1 | -0.0705533 | 0.104964 | -0.67217 | 0.501477 | 0.771271 |
| PABPC5-AS1 | 0.073135901 | 0.108696 | 0.672851 | 0.501042 | 0.771271 |
| RP1-59M18.2 | 0.056195077 | 0.083548 | 0.67261 | 0.501196 | 0.771271 |
| RP11-389G6.3 | -0.079296753 | 0.117772 | -0.67331 | 0.500751 | 0.771271 |
| PRKAR2A-AS1 | -0.054270274 | 0.080905 | -0.67079 | 0.502352 | 0.771588 |
| RP11-16E12.1 | 0.062317947 | 0.0929 | 0.670809 | 0.502342 | 0.771588 |
| RP11-125B21.2 | 0.052819808 | 0.078875 | 0.669669 | 0.503069 | 0.771861 |
| RP11-342K6.2 | -0.058796494 | 0.087826 | -0.66947 | 0.503198 | 0.771861 |
| RP11-316E14.6 | 0.059239125 | 0.088701 | 0.667849 | 0.50423 | 0.772416 |
| RP4-694B14.5 | -0.05961873 | 0.089233 | -0.66812 | 0.504054 | 0.772416 |
| RP11-225B17.2 | -0.053017125 | 0.079614 | -0.66592 | 0.50546 | 0.77276 |
| RP11-296O14.3 | -0.04663316 | 0.070021 | -0.66599 | 0.505418 | 0.77276 |
| RP11-725P16.2 | -0.081854213 | 0.12289 | -0.66608 | 0.505362 | 0.77276 |
| RP11-477D19.2 | -0.048959028 | 0.07368 | -0.66448 | 0.506384 | 0.773661 |
| CTD-2017D11.1 | -0.033836045 | 0.051061 | -0.66267 | 0.507545 | 0.774409 |
| RP11-469M7.1 | 0.054824682 | 0.082704 | 0.662904 | 0.507392 | 0.774409 |
| RP11-1072C15.4 | -0.049669107 | 0.075053 | -0.66179 | 0.508107 | 0.774755 |
| FAM66E | 0.06619812 | 0.100132 | 0.661107 | 0.508543 | 0.774907 |
| FTO-IT1 | 0.057306972 | 0.086984 | 0.658825 | 0.510008 | 0.775259 |
| RP11-255C15.3 | -0.073607721 | 0.111755 | -0.65865 | 0.510118 | 0.775259 |
| RP11-65J21.3 | 0.065723073 | 0.099575 | 0.660034 | 0.509232 | 0.775259 |
| RP5-1142A6.9 | 0.063733978 | 0.096695 | 0.659127 | 0.509814 | 0.775259 |
| AC093415.2 | 0.038403442 | 0.058447 | 0.657061 | 0.511142 | 0.776303 |
| RP11-158I13.2 | 0.039212289 | 0.059811 | 0.655601 | 0.512081 | 0.776706 |
| RP11-16E18.3 | -0.059333236 | 0.090452 | -0.65596 | 0.511849 | 0.776706 |
| AC005592.3 | 0.07309946 | 0.111812 | 0.65377 | 0.51326 | 0.777429 |
| RP11-769N19.2 | -0.073629921 | 0.112661 | -0.65355 | 0.5134 | 0.777429 |
| SH3BP5-AS1 | -0.048901367 | 0.074854 | -0.65329 | 0.513568 | 0.777429 |
| AP001469.9 | -0.038653643 | 0.059265 | -0.65222 | 0.514261 | 0.777458 |
| RP11-173A16.2 | -0.054993576 | 0.084288 | -0.65245 | 0.514111 | 0.777458 |
| RP13-514E23.2 | 0.072525359 | 0.111528 | 0.650291 | 0.515504 | 0.778317 |
| SCARNA2 | -0.05651381 | 0.086898 | -0.65035 | 0.515466 | 0.778317 |
| RP11-21J18.1 | 0.047295611 | 0.073008 | 0.647813 | 0.517106 | 0.779714 |
| RP11-561O23.5 | 0.088213814 | 0.136119 | 0.648062 | 0.516945 | 0.779714 |
| FAM85B | 0.068578639 | 0.105996 | 0.646991 | 0.517638 | 0.779994 |
| RP11-357C3.3 | 0.042414713 | 0.065609 | 0.646481 | 0.517968 | 0.779994 |
| RP11-1002K11.1 | 0.079540041 | 0.123235 | 0.645436 | 0.518645 | 0.780504 |
| C1RL-AS1 | 0.062594182 | 0.097203 | 0.64395 | 0.519608 | 0.780935 |
| RP11-141B14.1 | 0.065153706 | 0.101131 | 0.644253 | 0.519412 | 0.780935 |
| RP11-57H12.3 | -0.05629616 | 0.087647 | -0.6423 | 0.520676 | 0.782031 |
| CTB-181H17.1 | 0.063063321 | 0.098524 | 0.640082 | 0.52212 | 0.782671 |
| EXTL3-AS1 | -0.048809307 | 0.076234 | -0.64026 | 0.522004 | 0.782671 |
| RP11-298J20.4 | -0.043339801 | 0.067681 | -0.64035 | 0.521943 | 0.782671 |
| RP1-39G22.7 | 0.041990676 | 0.065809 | 0.638064 | 0.523432 | 0.783619 |
| RP11-311C24.1 | -0.04452303 | 0.069729 | -0.63851 | 0.52314 | 0.783619 |
| RP11-456K23.1 | -0.064486716 | 0.101297 | -0.63661 | 0.524378 | 0.784527 |
| AC007405.4 | -0.051925661 | 0.081922 | -0.63384 | 0.526183 | 0.785698 |
| ARL5B-AS1 | -0.042482461 | 0.06697 | -0.63435 | 0.525853 | 0.785698 |
| RP11-455F5.3 | 0.053463405 | 0.084278 | 0.634372 | 0.525838 | 0.785698 |
| RP11-78F17.1 | 0.061831693 | 0.098424 | 0.628219 | 0.529861 | 0.790678 |
| CTB-151G24.1 | -0.052942904 | 0.08449 | -0.62661 | 0.530912 | 0.791735 |
| AP001062.7 | -0.052357121 | 0.084426 | -0.62015 | 0.535156 | 0.793582 |
| CASC15 | 0.033856243 | 0.054428 | 0.622043 | 0.533914 | 0.793582 |
| CD27-AS1 | -0.046316698 | 0.074505 | -0.62166 | 0.534168 | 0.793582 |
| LINC00467 | -0.063424886 | 0.101743 | -0.62338 | 0.533032 | 0.793582 |
| LINC00996 | -0.084279586 | 0.135833 | -0.62046 | 0.534952 | 0.793582 |
| RP11-161M6.2 | 0.046290984 | 0.074271 | 0.623273 | 0.533105 | 0.793582 |
| RP11-212P7.2 | -0.038701975 | 0.062526 | -0.61897 | 0.535934 | 0.793582 |
| RP11-380O24.1 | -0.072452909 | 0.116786 | -0.62039 | 0.535002 | 0.793582 |
| RP11-582J16.5 | 0.055304508 | 0.089329 | 0.619107 | 0.535846 | 0.793582 |
| RP5-1070A16.1 | 0.068644728 | 0.110806 | 0.619506 | 0.535583 | 0.793582 |
| ZFAS1 | -0.05608841 | 0.090512 | -0.61968 | 0.53547 | 0.793582 |
| KIF9-AS1 | 0.034095475 | 0.055219 | 0.617459 | 0.536932 | 0.79404 |
| RP5-1068E13.7 | -0.061029827 | 0.09884 | -0.61746 | 0.536932 | 0.79404 |
| LINC00951 | 0.074784817 | 0.121378 | 0.616131 | 0.537808 | 0.794087 |
| PLCB1-IT1 | -0.06017872 | 0.09763 | -0.61639 | 0.537634 | 0.794087 |
| RP11-909M7.3 | 0.05299166 | 0.086047 | 0.615845 | 0.537997 | 0.794087 |
| GS1-358P8.4 | 0.05434638 | 0.088357 | 0.615076 | 0.538505 | 0.794329 |
| CTD-3220F14.2 | -0.053319161 | 0.086858 | -0.61387 | 0.539304 | 0.794999 |
| AC010127.3 | 0.066126664 | 0.108221 | 0.611035 | 0.541176 | 0.795525 |
| LINC00846 | 0.04832097 | 0.079121 | 0.61072 | 0.541385 | 0.795525 |
| RP11-432J22.2 | 0.050265792 | 0.082217 | 0.611377 | 0.54095 | 0.795525 |
| RP11-509E10.1 | 0.068917377 | 0.112691 | 0.611558 | 0.54083 | 0.795525 |
| RP11-649A18.7 | -0.054241073 | 0.088519 | -0.61276 | 0.540035 | 0.795525 |
| RP11-403I13.4 | 0.05620764 | 0.092194 | 0.609664 | 0.542084 | 0.79589 |
| RP11-82L18.2 | -0.049849608 | 0.08186 | -0.60896 | 0.542551 | 0.79589 |
| RP11-876N24.5 | -0.067969951 | 0.111649 | -0.60878 | 0.542668 | 0.79589 |
| FAM201A | 0.056287777 | 0.092565 | 0.608091 | 0.543127 | 0.796058 |
| AC034220.3 | 0.028517032 | 0.047042 | 0.606202 | 0.54438 | 0.796438 |
| RP11-156E6.1 | -0.037834678 | 0.062315 | -0.60715 | 0.543748 | 0.796438 |
| RP11-163N6.2 | -0.061530638 | 0.101512 | -0.60614 | 0.544422 | 0.796438 |
| CTB-96E2.3 | 0.044701673 | 0.073964 | 0.604368 | 0.545599 | 0.796964 |
| LINC01011 | 0.045640215 | 0.075568 | 0.603963 | 0.545868 | 0.796964 |
| LINC01057 | -0.063658418 | 0.105479 | -0.60352 | 0.546164 | 0.796964 |
| RP11-84A19.2 | 0.053684468 | 0.08874 | 0.604967 | 0.545201 | 0.796964 |
| RP11-169K16.9 | -0.044500214 | 0.073905 | -0.60213 | 0.547087 | 0.797776 |
| RP11-541N10.3 | 0.061157922 | 0.101652 | 0.601643 | 0.547412 | 0.797776 |
| ADAMTS9-AS2 | 0.056056235 | 0.093381 | 0.600295 | 0.54831 | 0.79858 |
| CTD-2015H6.3 | 0.048952969 | 0.081792 | 0.598507 | 0.549502 | 0.799811 |
| RP11-469N6.1 | 0.08148809 | 0.137175 | 0.594046 | 0.552481 | 0.803641 |
| AC005614.3 | -0.057363862 | 0.096657 | -0.59348 | 0.55286 | 0.803685 |
| RP11-750H9.7 | 0.056942918 | 0.096379 | 0.590824 | 0.554638 | 0.805255 |
| RP11-77P16.4 | -0.037619534 | 0.06367 | -0.59085 | 0.554621 | 0.805255 |
| RP11-108M9.4 | 0.071450872 | 0.121349 | 0.588803 | 0.555993 | 0.805802 |
| RP11-479O17.10 | -0.056350576 | 0.09572 | -0.5887 | 0.556063 | 0.805802 |
| RP3-462E2.5 | -0.047034249 | 0.079776 | -0.58958 | 0.555472 | 0.805802 |
| STAG3L5P-PVRIG2P-PILRB | 0.057529033 | 0.097851 | 0.587924 | 0.556583 | 0.80605 |
| LINC00106 | 0.058594503 | 0.099965 | 0.586148 | 0.557776 | 0.807271 |
| RP11-66N24.3 | 0.05772801 | 0.098628 | 0.585311 | 0.558339 | 0.807578 |
| AKT3-IT1 | -0.041443081 | 0.071015 | -0.58358 | 0.559504 | 0.807744 |
| MIR210HG | 0.077997972 | 0.133505 | 0.584234 | 0.559063 | 0.807744 |
| RP11-57H14.4 | 0.036853324 | 0.063141 | 0.583665 | 0.559445 | 0.807744 |
| AC007743.1 | -0.042049014 | 0.072221 | -0.58222 | 0.560415 | 0.808355 |
| RP11-782C8.5 | -0.078758203 | 0.135344 | -0.58191 | 0.560628 | 0.808355 |
| AC007383.3 | -0.055745927 | 0.096204 | -0.57946 | 0.562282 | 0.810009 |
| AC108142.1 | 0.051066766 | 0.088173 | 0.579166 | 0.562477 | 0.810009 |
| LINC00116 | 0.052342557 | 0.090588 | 0.577809 | 0.563393 | 0.810023 |
| RP11-1114A5.4 | -0.03476039 | 0.060236 | -0.57707 | 0.563891 | 0.810023 |
| RP11-13A1.1 | 0.06548587 | 0.113473 | 0.577106 | 0.563868 | 0.810023 |
| SNX29P2 | -0.032411553 | 0.056051 | -0.57825 | 0.563097 | 0.810023 |
| CECR7 | -0.04453078 | 0.077611 | -0.57377 | 0.566125 | 0.810343 |
| CTC-510F12.4 | -0.054852995 | 0.095798 | -0.57259 | 0.566924 | 0.810343 |
| FAM66D | 0.074259871 | 0.129532 | 0.573294 | 0.566445 | 0.810343 |
| HCG17 | 0.0561643 | 0.097768 | 0.574467 | 0.565652 | 0.810343 |
| LINC00499 | 0.076196844 | 0.133048 | 0.572701 | 0.566847 | 0.810343 |
| LINC00959 | 0.058530363 | 0.102006 | 0.573793 | 0.566108 | 0.810343 |
| RP1-197B17.3 | -0.062693801 | 0.108935 | -0.57551 | 0.564943 | 0.810343 |
| RP11-454P21.1 | 0.053988659 | 0.094043 | 0.574087 | 0.565909 | 0.810343 |
| LINC01004 | -0.047311102 | 0.082835 | -0.57115 | 0.567897 | 0.811231 |
| RP11-192H23.8 | 0.051325053 | 0.089955 | 0.570564 | 0.568295 | 0.811297 |
| CTD-2007H13.3 | -0.058792326 | 0.103154 | -0.56995 | 0.568713 | 0.811392 |
| WI2-81516E3.1 | 0.060773963 | 0.106818 | 0.568949 | 0.569391 | 0.811857 |
| U82695.10 | 0.039063153 | 0.068911 | 0.566864 | 0.570807 | 0.812871 |
| USP27X-AS1 | 0.055121855 | 0.097217 | 0.567 | 0.570714 | 0.812871 |
| RP11-230C9.2 | -0.047913257 | 0.084621 | -0.56621 | 0.571251 | 0.813003 |
| PWRN3 | -0.075881423 | 0.134215 | -0.56537 | 0.571821 | 0.813311 |
| AC093323.3 | 0.039459851 | 0.070462 | 0.560016 | 0.575469 | 0.81626 |
| AP000476.1 | -0.063016975 | 0.112415 | -0.56057 | 0.575089 | 0.81626 |
| RP11-355I22.7 | 0.054086803 | 0.096461 | 0.560712 | 0.574994 | 0.81626 |
| RP11-368I23.2 | 0.066743668 | 0.119076 | 0.560514 | 0.575129 | 0.81626 |
| ZNF346-IT1 | -0.043355328 | 0.077458 | -0.55973 | 0.575663 | 0.81626 |
| AC007389.3 | -0.036722922 | 0.066744 | -0.55021 | 0.582178 | 0.816885 |
| AC016745.3 | 0.061092107 | 0.110987 | 0.550445 | 0.582014 | 0.816885 |
| AC092295.7 | -0.044405656 | 0.080368 | -0.55253 | 0.580586 | 0.816885 |
| AC138430.4 | 0.068282834 | 0.122541 | 0.557225 | 0.577374 | 0.816885 |
| AL132709.8 | 0.05344412 | 0.09736 | 0.548932 | 0.583052 | 0.816885 |
| CTD-2023N9.3 | -0.063288541 | 0.115359 | -0.54862 | 0.583264 | 0.816885 |
| CTD-2334D19.1 | 0.058443207 | 0.105251 | 0.555273 | 0.578708 | 0.816885 |
| CYP17A1-AS1 | 0.055968847 | 0.10159 | 0.55093 | 0.581682 | 0.816885 |
| LINC00526 | -0.049603069 | 0.090182 | -0.55003 | 0.582296 | 0.816885 |
| RP1-179N16.6 | 0.056385536 | 0.101386 | 0.556149 | 0.578109 | 0.816885 |
| RP11-258F1.1 | -0.074041825 | 0.134178 | -0.55182 | 0.581074 | 0.816885 |
| RP11-315I14.2 | 0.057159001 | 0.103942 | 0.549913 | 0.582379 | 0.816885 |
| RP11-343H19.2 | -0.071859808 | 0.129578 | -0.55457 | 0.57919 | 0.816885 |
| RP11-359B12.2 | -0.035362748 | 0.064292 | -0.55003 | 0.582298 | 0.816885 |
| RP11-454K7.1 | 0.054584715 | 0.097886 | 0.557637 | 0.577092 | 0.816885 |
| RP11-457M11.5 | 0.049235011 | 0.089809 | 0.548221 | 0.58354 | 0.816885 |
| RP11-506O24.2 | -0.058884608 | 0.107012 | -0.55026 | 0.582138 | 0.816885 |
| RP11-509J21.3 | -0.062077834 | 0.112728 | -0.55069 | 0.581849 | 0.816885 |
| RP11-76N22.2 | -0.052262171 | 0.095305 | -0.54837 | 0.583438 | 0.816885 |
| RP6-24A23.7 | 0.060738815 | 0.109626 | 0.554054 | 0.579542 | 0.816885 |
| TMEM72-AS1 | 0.042690262 | 0.077018 | 0.554292 | 0.579379 | 0.816885 |
| AC064875.2 | -0.050724694 | 0.092773 | -0.54676 | 0.584541 | 0.817295 |
| SYNJ2BP-COX16 | -0.039270128 | 0.071806 | -0.54689 | 0.584452 | 0.817295 |
| AC079610.2 | -0.054838557 | 0.101059 | -0.54264 | 0.587377 | 0.819273 |
| COX10-AS1 | 0.031085073 | 0.057237 | 0.543095 | 0.587064 | 0.819273 |
| RP11-216M21.7 | 0.061259658 | 0.112585 | 0.544118 | 0.58636 | 0.819273 |
| RP11-573N10.1 | -0.052164292 | 0.096047 | -0.54311 | 0.587052 | 0.819273 |
| RP11-213G2.2 | -0.053723536 | 0.099307 | -0.54099 | 0.588517 | 0.82027 |
| RP11-869B15.1 | -0.045479778 | 0.084133 | -0.54057 | 0.588802 | 0.82027 |
| CTD-2135D7.2 | -0.054876427 | 0.101648 | -0.53987 | 0.589288 | 0.820452 |
| AC003102.3 | 0.038647649 | 0.071794 | 0.538314 | 0.59036 | 0.821173 |
| LINC00654 | -0.049646236 | 0.092457 | -0.53697 | 0.591291 | 0.821173 |
| RP11-438B23.2 | 0.0611619 | 0.113994 | 0.536538 | 0.591586 | 0.821173 |
| RP5-1063M23.1 | 0.068162317 | 0.126997 | 0.536724 | 0.591459 | 0.821173 |
| ZBED5-AS1 | -0.046531015 | 0.086629 | -0.53713 | 0.591178 | 0.821173 |
| JAZF1-AS1 | 0.058664348 | 0.109785 | 0.534357 | 0.593094 | 0.82129 |
| RP11-508N22.12 | 0.042879419 | 0.080083 | 0.535435 | 0.592349 | 0.82129 |
| RP11-61A14.2 | -0.046260762 | 0.086565 | -0.5344 | 0.593061 | 0.82129 |
| RP5-1158E12.3 | 0.054923629 | 0.1027 | 0.534796 | 0.592791 | 0.82129 |
| LINC00595 | -0.05138168 | 0.096542 | -0.53222 | 0.594573 | 0.822844 |
| RP11-155G14.6 | 0.072093091 | 0.135697 | 0.531281 | 0.595224 | 0.82325 |
| LINC00488 | 0.065974326 | 0.12458 | 0.529573 | 0.596408 | 0.824394 |
| RP11-214K3.19 | 0.050057253 | 0.094717 | 0.528491 | 0.597158 | 0.824937 |
| RP11-173D3.1 | 0.058162755 | 0.110248 | 0.527565 | 0.597801 | 0.825053 |
| RP5-886K2.3 | -0.041603731 | 0.078894 | -0.52734 | 0.597958 | 0.825053 |
| RP11-271K21.11 | 0.034452838 | 0.065557 | 0.525537 | 0.59921 | 0.826286 |
| MIR22HG | 0.05387867 | 0.102777 | 0.524228 | 0.60012 | 0.826553 |
| PTPRG-AS1 | 0.039473059 | 0.075249 | 0.524569 | 0.599883 | 0.826553 |
| LINC00900 | 0.047403956 | 0.090756 | 0.522323 | 0.601446 | 0.827885 |
| JHDM1D-AS1 | 0.050288444 | 0.096826 | 0.519371 | 0.603502 | 0.828829 |
| LINC00622 | 0.058733252 | 0.113106 | 0.519276 | 0.603568 | 0.828829 |
| RP11-1008C21.2 | -0.048644127 | 0.093669 | -0.51932 | 0.603537 | 0.828829 |
| RP11-290D2.3 | -0.053440098 | 0.102634 | -0.52068 | 0.602587 | 0.828829 |
| RP11-178L8.8 | -0.037502821 | 0.072453 | -0.51761 | 0.604728 | 0.829926 |
| CTC-297N7.8 | 0.038813918 | 0.075152 | 0.516476 | 0.605522 | 0.830336 |
| RP11-307P5.1 | -0.05966034 | 0.115586 | -0.51616 | 0.605745 | 0.830336 |
| UBA6-AS1 | 0.027875459 | 0.054063 | 0.515607 | 0.606129 | 0.830367 |
| AC007796.1 | 0.055905859 | 0.109107 | 0.512393 | 0.608376 | 0.83138 |
| AF131216.5 | 0.059966793 | 0.117135 | 0.511946 | 0.608688 | 0.83138 |
| LINC00665 | 0.022724525 | 0.044416 | 0.511626 | 0.608913 | 0.83138 |
| LINC00865 | -0.06376238 | 0.124456 | -0.51233 | 0.608422 | 0.83138 |
| RP11-49C24.1 | 0.045619753 | 0.089195 | 0.511458 | 0.60903 | 0.83138 |
| RP11-996F15.2 | -0.057359666 | 0.111945 | -0.51239 | 0.608377 | 0.83138 |
| CTC-471J1.8 | 0.039036273 | 0.076928 | 0.507439 | 0.611847 | 0.832165 |
| GS1-124K5.4 | -0.048262379 | 0.095462 | -0.50557 | 0.61316 | 0.832165 |
| RP11-1C8.4 | 0.045623739 | 0.089598 | 0.509204 | 0.610609 | 0.832165 |
| RP11-252A24.7 | 0.030100908 | 0.059548 | 0.505494 | 0.613212 | 0.832165 |
| RP11-379B18.5 | -0.036066534 | 0.070719 | -0.51 | 0.610053 | 0.832165 |
| RP11-398H6.1 | -0.040110496 | 0.079197 | -0.50646 | 0.612532 | 0.832165 |
| RP11-483P21.3 | 0.050440372 | 0.09945 | 0.507192 | 0.61202 | 0.832165 |
| RP11-707A18.1 | 0.056208627 | 0.110945 | 0.506635 | 0.612411 | 0.832165 |
| RP5-827C21.6 | 0.057742365 | 0.113831 | 0.507262 | 0.611971 | 0.832165 |
| ZNF667-AS1 | 0.041408465 | 0.081489 | 0.50815 | 0.611348 | 0.832165 |
| ILF3-AS1 | 0.032239457 | 0.063967 | 0.504001 | 0.614261 | 0.833098 |
| RP11-187C18.2 | -0.050720447 | 0.100821 | -0.50307 | 0.614913 | 0.833492 |
| LINC00987 | -0.051170751 | 0.102363 | -0.4999 | 0.617148 | 0.835461 |
| RP1-97G4.1 | -0.052541032 | 0.105067 | -0.50007 | 0.617024 | 0.835461 |
| RP11-539L10.3 | 0.046085822 | 0.09227 | 0.499466 | 0.617451 | 0.835461 |
| RP11-108P20.1 | -0.043804721 | 0.08794 | -0.49812 | 0.618398 | 0.83584 |
| RP11-392E22.11 | 0.056590892 | 0.113627 | 0.498039 | 0.618456 | 0.83584 |
| RP11-498P14.5 | 0.061861314 | 0.12438 | 0.497357 | 0.618937 | 0.836 |
| CTA-390C10.10 | -0.045366888 | 0.091566 | -0.49546 | 0.620279 | 0.836038 |
| LENG8-AS1 | -0.035044101 | 0.070754 | -0.49529 | 0.620393 | 0.836038 |
| RP11-482H16.1 | 0.04921643 | 0.099375 | 0.495262 | 0.620415 | 0.836038 |
| RP6-206I17.1 | 0.050600636 | 0.101919 | 0.496481 | 0.619555 | 0.836038 |
| ZNRF3-IT1 | -0.047774068 | 0.096672 | -0.49419 | 0.621174 | 0.836572 |
| AC005519.4 | 0.03329691 | 0.068053 | 0.489281 | 0.624643 | 0.837062 |
| CASC2 | -0.028719617 | 0.058387 | -0.49188 | 0.622802 | 0.837062 |
| HMGN3-AS1 | -0.041022781 | 0.083802 | -0.48952 | 0.624473 | 0.837062 |
| LINC00842 | 0.055989563 | 0.113922 | 0.491472 | 0.623092 | 0.837062 |
| LINC00852 | 0.048203342 | 0.098494 | 0.489406 | 0.624554 | 0.837062 |
| RP11-374A22.1 | -0.056047298 | 0.114003 | -0.49163 | 0.622982 | 0.837062 |
| RP11-617F23.1 | -0.039478585 | 0.080603 | -0.48979 | 0.624281 | 0.837062 |
| RP11-773D16.1 | -0.033387388 | 0.067776 | -0.49262 | 0.622284 | 0.837062 |
| RP4-561L24.3 | 0.03996972 | 0.081729 | 0.489054 | 0.624803 | 0.837062 |
| AC005154.6 | -0.024547782 | 0.050377 | -0.48728 | 0.626061 | 0.837497 |
| U73166.2 | 0.043203096 | 0.088702 | 0.487058 | 0.626217 | 0.837497 |
| XXbac-B476C20.13 | 0.048883775 | 0.100341 | 0.487176 | 0.626134 | 0.837497 |
| RP11-118K6.3 | -0.047659372 | 0.098041 | -0.48612 | 0.626884 | 0.837849 |
| RP3-523C21.2 | -0.052420994 | 0.107937 | -0.48566 | 0.627206 | 0.837849 |
| ARHGAP26-IT1 | -0.053432643 | 0.110433 | -0.48385 | 0.628495 | 0.839084 |
| AC018464.3 | 0.04729816 | 0.098106 | 0.482113 | 0.629725 | 0.839755 |
| AC135048.13 | -0.033813393 | 0.070062 | -0.48262 | 0.629367 | 0.839755 |
| AC105760.2 | -0.042843841 | 0.089848 | -0.47685 | 0.63347 | 0.843745 |
| JRK | 0.026579816 | 0.055775 | 0.476555 | 0.633679 | 0.843745 |
| RP11-87E22.2 | 0.063103546 | 0.132469 | 0.476365 | 0.633814 | 0.843745 |
| RP11-397A16.2 | -0.042187031 | 0.089009 | -0.47396 | 0.635527 | 0.845536 |
| CTD-3195I5.5 | -0.043181794 | 0.091709 | -0.47086 | 0.637742 | 0.846396 |
| RP11-1006G14.4 | 0.030044134 | 0.063927 | 0.469974 | 0.638374 | 0.846396 |
| RP11-16E23.3 | -0.053206206 | 0.113071 | -0.47056 | 0.637958 | 0.846396 |
| RP11-193F5.1 | 0.034232198 | 0.072496 | 0.472194 | 0.636788 | 0.846396 |
| RP11-85O21.2 | 0.050482209 | 0.107303 | 0.470462 | 0.638025 | 0.846396 |
| SMC5-AS1 | 0.038792237 | 0.082396 | 0.470805 | 0.63778 | 0.846396 |
| AC016683.6 | 0.062910161 | 0.134666 | 0.467157 | 0.640388 | 0.846633 |
| CTD-2554C21.3 | 0.038055504 | 0.081366 | 0.46771 | 0.639992 | 0.846633 |
| GDNF-AS1 | -0.049498058 | 0.105923 | -0.4673 | 0.640284 | 0.846633 |
| KANSL1-AS1 | 0.064006416 | 0.137009 | 0.46717 | 0.640378 | 0.846633 |
| MLLT4-AS1 | 0.046186161 | 0.098601 | 0.468415 | 0.639488 | 0.846633 |
| CTA-14H9.5 | -0.045520146 | 0.097666 | -0.46608 | 0.641158 | 0.847166 |
| LINC00883 | -0.039322488 | 0.08467 | -0.46442 | 0.642348 | 0.847823 |
| RP11-553A21.3 | 0.05000848 | 0.107694 | 0.464359 | 0.642391 | 0.847823 |
| PPP1R26-AS1 | 0.046783348 | 0.101125 | 0.462628 | 0.643631 | 0.848975 |
| RP11-705C15.5 | -0.045091498 | 0.097727 | -0.4614 | 0.644509 | 0.849647 |
| RASAL2-AS1 | 0.038987302 | 0.084617 | 0.460749 | 0.644979 | 0.849781 |
| AP000230.1 | 0.048856993 | 0.106379 | 0.459274 | 0.646038 | 0.850512 |
| HECW1-IT1 | -0.054105524 | 0.118101 | -0.45813 | 0.64686 | 0.850512 |
| LINC00339 | 0.044730108 | 0.097729 | 0.457693 | 0.647173 | 0.850512 |
| RP11-488C13.5 | 0.024796123 | 0.054271 | 0.456896 | 0.647746 | 0.850512 |
| RP11-677M14.7 | 0.042371795 | 0.092715 | 0.457012 | 0.647663 | 0.850512 |
| WARS2-IT1 | -0.052658825 | 0.115038 | -0.45775 | 0.64713 | 0.850512 |
| RP11-154H23.3 | -0.045200252 | 0.099117 | -0.45603 | 0.648368 | 0.850844 |
| RP11-297D21.4 | -0.037091352 | 0.081458 | -0.45535 | 0.64886 | 0.851007 |
| ENO1-IT1 | -0.050509972 | 0.111168 | -0.45436 | 0.649571 | 0.851017 |
| RP11-337C18.10 | -0.045009496 | 0.099174 | -0.45384 | 0.649941 | 0.851017 |
| RP11-672L10.2 | 0.059360575 | 0.130809 | 0.453797 | 0.649975 | 0.851017 |
| AP000662.4 | 0.039446565 | 0.087083 | 0.452975 | 0.650567 | 0.851309 |
| LINC00115 | 0.043736912 | 0.097113 | 0.450371 | 0.652443 | 0.85135 |
| LINC00843 | 0.031714688 | 0.070133 | 0.452207 | 0.65112 | 0.85135 |
| RP11-319G6.1 | -0.027823251 | 0.061762 | -0.45049 | 0.652358 | 0.85135 |
| RP11-543C4.1 | 0.041904453 | 0.092992 | 0.450623 | 0.652261 | 0.85135 |
| RP5-1159O4.2 | -0.045183326 | 0.100239 | -0.45075 | 0.652167 | 0.85135 |
| AC006272.1 | 0.051641578 | 0.11518 | 0.448355 | 0.653897 | 0.852765 |
| CTD-2349P21.9 | 0.032866001 | 0.073466 | 0.447362 | 0.654614 | 0.853217 |
| RP11-1096G20.5 | 0.040162401 | 0.09021 | 0.445209 | 0.656169 | 0.854761 |
| C17orf76-AS1 | -0.035195354 | 0.079531 | -0.44254 | 0.658102 | 0.855829 |
| RP11-109D24.1 | 0.056665932 | 0.127809 | 0.443364 | 0.657502 | 0.855829 |
| RP11-144G6.12 | 0.043575445 | 0.098435 | 0.442684 | 0.657995 | 0.855829 |
| RP11-890B15.3 | -0.030940708 | 0.070038 | -0.44177 | 0.658654 | 0.856065 |
| LINC00643 | -0.047131059 | 0.107239 | -0.4395 | 0.660302 | 0.857003 |
| RP11-197N18.2 | 0.034565767 | 0.078695 | 0.439236 | 0.660491 | 0.857003 |
| RP11-392E22.12 | 0.052901774 | 0.120209 | 0.440081 | 0.659879 | 0.857003 |
| BX255923.3 | 0.059363796 | 0.135867 | 0.436926 | 0.662165 | 0.858693 |
| LINC00969 | 0.042356321 | 0.097253 | 0.435527 | 0.66318 | 0.859209 |
| RP11-77P6.2 | -0.040035906 | 0.091962 | -0.43535 | 0.663308 | 0.859209 |
| KB-1507C5.4 | -0.044672229 | 0.10377 | -0.43049 | 0.666838 | 0.861364 |
| KB-226F1.1 | -0.046097707 | 0.106993 | -0.43085 | 0.66658 | 0.861364 |
| KCTD21-AS1 | -0.048222433 | 0.111911 | -0.4309 | 0.666541 | 0.861364 |
| LINC00271 | 0.036323601 | 0.084346 | 0.43065 | 0.666723 | 0.861364 |
| LINC00685 | 0.04266445 | 0.098771 | 0.431955 | 0.665774 | 0.861364 |
| CTC-260E6.6 | -0.040290973 | 0.094532 | -0.42621 | 0.669952 | 0.86297 |
| KB-318B8.7 | 0.034844911 | 0.081728 | 0.426353 | 0.669851 | 0.86297 |
| MAMDC2-AS1 | 0.032554728 | 0.076352 | 0.426377 | 0.669833 | 0.86297 |
| RP11-45M22.5 | 0.044793027 | 0.104672 | 0.427937 | 0.668697 | 0.86297 |
| RP11-715J22.2 | -0.032220805 | 0.075598 | -0.42621 | 0.669952 | 0.86297 |
| RP11-71H17.7 | 0.034087805 | 0.080239 | 0.424827 | 0.670962 | 0.863789 |
| AC012307.3 | 0.032319293 | 0.076945 | 0.420032 | 0.674462 | 0.866693 |
| AC068057.2 | -0.033326903 | 0.079704 | -0.41814 | 0.675848 | 0.866693 |
| LINC00907 | -0.033095723 | 0.079103 | -0.41839 | 0.675662 | 0.866693 |
| RP11-218E20.3 | -0.04549596 | 0.108437 | -0.41956 | 0.674807 | 0.866693 |
| RP11-504P24.4 | -0.044250125 | 0.105271 | -0.42035 | 0.674233 | 0.866693 |
| RP11-73K9.2 | 0.038743619 | 0.091984 | 0.4212 | 0.673609 | 0.866693 |
| RP11-797A18.4 | 0.040786382 | 0.097322 | 0.419089 | 0.675151 | 0.866693 |
| RP11-397O4.1 | 0.046624689 | 0.111794 | 0.417061 | 0.676634 | 0.867219 |
| RP11-339B21.15 | -0.042520732 | 0.102302 | -0.41564 | 0.677674 | 0.86807 |
| AC008746.12 | 0.043817166 | 0.105704 | 0.414527 | 0.678488 | 0.868631 |
| FAM157A | -0.049512176 | 0.119778 | -0.41337 | 0.679338 | 0.869236 |
| RP5-837J1.2 | 0.021924051 | 0.053109 | 0.412815 | 0.679742 | 0.869271 |
| AF131215.2 | -0.028801577 | 0.069863 | -0.41226 | 0.680149 | 0.86931 |
| AC079922.3 | 0.040710972 | 0.099405 | 0.409546 | 0.682139 | 0.869445 |
| CTD-2547L16.1 | 0.036528001 | 0.08911 | 0.409922 | 0.681863 | 0.869445 |
| RP11-435B5.5 | -0.056237508 | 0.13718 | -0.40995 | 0.681839 | 0.869445 |
| RP11-46A10.5 | 0.029872699 | 0.072625 | 0.411327 | 0.680833 | 0.869445 |
| ZNF582-AS1 | 0.030121632 | 0.073472 | 0.409975 | 0.681824 | 0.869445 |
| TSIX | -0.033118731 | 0.081148 | -0.40813 | 0.683179 | 0.870289 |
| CTC-459F4.1 | -0.044658078 | 0.109886 | -0.4064 | 0.684447 | 0.871423 |
| RP11-245J24.1 | -0.049415466 | 0.122453 | -0.40354 | 0.686547 | 0.872246 |
| RP11-333I13.1 | 0.038585467 | 0.095455 | 0.404226 | 0.686046 | 0.872246 |
| RP3-462E2.3 | -0.038944838 | 0.096244 | -0.40465 | 0.685736 | 0.872246 |
| RP5-1024G6.8 | 0.02656617 | 0.065845 | 0.403466 | 0.686605 | 0.872246 |
| RP11-195C7.1 | -0.037743884 | 0.093697 | -0.40283 | 0.687073 | 0.872359 |
| AC114877.3 | -0.04949806 | 0.124923 | -0.39623 | 0.691936 | 0.878051 |
| RP11-1C8.5 | -0.034785863 | 0.088252 | -0.39417 | 0.693459 | 0.879016 |
| RP11-359E3.4 | -0.02801842 | 0.070999 | -0.39463 | 0.693115 | 0.879016 |
| RP11-314B1.2 | 0.032660058 | 0.083035 | 0.393328 | 0.694077 | 0.879317 |
| RP11-103G8.2 | -0.0356035 | 0.090693 | -0.39257 | 0.694637 | 0.879543 |
| KLF7-IT1 | 0.040870886 | 0.104654 | 0.390534 | 0.696142 | 0.880444 |
| RP11-203J24.9 | 0.029301918 | 0.075122 | 0.390059 | 0.696493 | 0.880444 |
| RP11-535M15.1 | 0.036400098 | 0.093115 | 0.390914 | 0.695861 | 0.880444 |
| GAS5 | -0.032412194 | 0.083856 | -0.38652 | 0.69911 | 0.882928 |
| SNHG9 | -0.041678883 | 0.107873 | -0.38637 | 0.699223 | 0.882928 |
| RP11-467K18.2 | 0.039592199 | 0.102685 | 0.385568 | 0.699817 | 0.883193 |
| RP11-1134I14.8 | 0.025704929 | 0.066765 | 0.385005 | 0.700233 | 0.883236 |
| MIMT1 | 0.038018093 | 0.099034 | 0.383888 | 0.701061 | 0.883797 |
| LL22NC03-2H8.5 | 0.037507217 | 0.097873 | 0.383221 | 0.701556 | 0.883937 |
| RP11-727A23.11 | 0.037024903 | 0.097296 | 0.380538 | 0.703546 | 0.885961 |
| RP11-40F8.2 | 0.037800562 | 0.099559 | 0.379679 | 0.704184 | 0.886281 |
| RP11-797A18.6 | -0.034385882 | 0.091191 | -0.37707 | 0.706118 | 0.888231 |
| LA16c-380H5.5 | 0.043735042 | 0.116849 | 0.374285 | 0.708192 | 0.888791 |
| RP11-1260E13.4 | -0.045755506 | 0.122358 | -0.37395 | 0.708442 | 0.888791 |
| RP11-441F2.2 | -0.033096425 | 0.088228 | -0.37512 | 0.707569 | 0.888791 |
| RP11-448G15.3 | -0.044502684 | 0.119028 | -0.37388 | 0.70849 | 0.888791 |
| RP11-700J17.2 | -0.04376204 | 0.116907 | -0.37433 | 0.708157 | 0.888791 |
| AF131215.3 | -0.034061774 | 0.091844 | -0.37087 | 0.710738 | 0.889193 |
| LINC00507 | -0.049931377 | 0.134608 | -0.37094 | 0.710682 | 0.889193 |
| NARF-IT1 | -0.034972034 | 0.094023 | -0.37195 | 0.70993 | 0.889193 |
| RP11-286H14.6 | 0.030561816 | 0.082261 | 0.371521 | 0.710249 | 0.889193 |
| RP11-688I9.4 | 0.039399862 | 0.105885 | 0.3721 | 0.709819 | 0.889193 |
| RP11-1018N14.5 | -0.042220721 | 0.114232 | -0.36961 | 0.711676 | 0.889885 |
| RP11-439E19.10 | -0.030623926 | 0.083176 | -0.36818 | 0.712736 | 0.890727 |
| RP3-327A19.5 | -0.026915324 | 0.073333 | -0.36703 | 0.713599 | 0.891322 |
| RP11-136K7.2 | -0.038717963 | 0.105716 | -0.36624 | 0.714183 | 0.891569 |
| RP11-156P1.3 | 0.019318396 | 0.052967 | 0.364723 | 0.715318 | 0.892021 |
| RP11-666A20.4 | 0.037136409 | 0.101748 | 0.364984 | 0.715123 | 0.892021 |
| AC012146.7 | 0.035165965 | 0.097035 | 0.362407 | 0.717048 | 0.892731 |
| ADAMTS9-AS1 | -0.048988965 | 0.135131 | -0.36253 | 0.716956 | 0.892731 |
| RP11-452L6.1 | 0.029041831 | 0.080028 | 0.362898 | 0.716681 | 0.892731 |
| RP11-196G18.23 | -0.032512508 | 0.090197 | -0.36046 | 0.718504 | 0.894061 |
| RP11-475O6.1 | 0.041240666 | 0.114602 | 0.359859 | 0.718953 | 0.894137 |
| RP11-698N11.4 | -0.025330873 | 0.070559 | -0.359 | 0.719595 | 0.894453 |
| AL157871.2 | -0.024326224 | 0.068594 | -0.35464 | 0.722858 | 0.895154 |
| AP001372.2 | -0.022685807 | 0.063899 | -0.35503 | 0.722569 | 0.895154 |
| CTBP1-AS2 | 0.019174231 | 0.05407 | 0.354619 | 0.722875 | 0.895154 |
| CTD-2292M16.8 | -0.03079712 | 0.086723 | -0.35512 | 0.722499 | 0.895154 |
| RP11-376M2.2 | -0.040178857 | 0.112742 | -0.35638 | 0.721557 | 0.895154 |
| RP11-768G7.2 | -0.032163073 | 0.090111 | -0.35693 | 0.721147 | 0.895154 |
| SNHG15 | -0.031835007 | 0.089729 | -0.35479 | 0.722748 | 0.895154 |
| RP11-214K3.22 | 0.034520431 | 0.097706 | 0.35331 | 0.723856 | 0.895888 |
| RP1-228H13.5 | 0.02867232 | 0.081297 | 0.352687 | 0.724323 | 0.895986 |
| TOPORS-AS1 | -0.033716232 | 0.096149 | -0.35067 | 0.725838 | 0.897378 |
| DNAJC27-AS1 | -0.020322177 | 0.058228 | -0.34901 | 0.72708 | 0.898432 |
| RP11-1263C18.1 | 0.031755823 | 0.091599 | 0.346683 | 0.728829 | 0.898749 |
| RP11-390K5.6 | -0.030409037 | 0.087521 | -0.34745 | 0.728254 | 0.898749 |
| RP11-419I17.1 | 0.036302094 | 0.10463 | 0.346957 | 0.728623 | 0.898749 |
| RP11-844P9.2 | 0.039847854 | 0.114969 | 0.346597 | 0.728894 | 0.898749 |
| RP11-171I2.2 | -0.036031038 | 0.10442 | -0.34506 | 0.73005 | 0.899693 |
| AC091969.1 | 0.032667375 | 0.095349 | 0.34261 | 0.731892 | 0.899951 |
| MIR7-3HG | 0.038366823 | 0.111805 | 0.343158 | 0.73148 | 0.899951 |
| RP11-222A11.1 | -0.046371266 | 0.135514 | -0.34219 | 0.73221 | 0.899951 |
| RP11-673E1.1 | 0.034336825 | 0.099908 | 0.343683 | 0.731085 | 0.899951 |
| RP4-785G19.5 | 0.027476085 | 0.080112 | 0.342973 | 0.731619 | 0.899951 |
| CTD-2196E14.4 | -0.02091468 | 0.061545 | -0.33983 | 0.733988 | 0.90013 |
| RP11-407G23.4 | 0.032088318 | 0.09401 | 0.341329 | 0.732856 | 0.90013 |
| RP11-414J4.2 | -0.040802894 | 0.120231 | -0.33937 | 0.734329 | 0.90013 |
| RP11-429B14.4 | -0.037245898 | 0.109779 | -0.33928 | 0.734398 | 0.90013 |
| RP11-78O7.2 | 0.036433892 | 0.107511 | 0.338884 | 0.734697 | 0.90013 |
| RP5-902P8.10 | 0.03127564 | 0.092221 | 0.33914 | 0.734505 | 0.90013 |
| RP11-932O9.7 | -0.03144029 | 0.093007 | -0.33804 | 0.735331 | 0.90043 |
| RP11-133F8.2 | 0.029581394 | 0.087801 | 0.336913 | 0.736183 | 0.900516 |
| RP11-298P3.4 | 0.019699699 | 0.05846 | 0.336975 | 0.736136 | 0.900516 |
| 7SK | -0.034022027 | 0.101457 | -0.33533 | 0.737373 | 0.901494 |
| RP5-857K21.4 | 0.035906343 | 0.107476 | 0.334086 | 0.738315 | 0.902167 |
| CTC-471C19.1 | -0.040762099 | 0.122267 | -0.33339 | 0.738843 | 0.902335 |
| RP11-711K1.7 | 0.034815676 | 0.104698 | 0.332536 | 0.739485 | 0.902641 |
| RP11-421L21.3 | 0.031003015 | 0.093636 | 0.331102 | 0.740567 | 0.903429 |
| RP4-635E18.8 | -0.019226992 | 0.05815 | -0.33064 | 0.740914 | 0.903429 |
| LINC00664 | -0.039873823 | 0.12142 | -0.3284 | 0.742612 | 0.903718 |
| RP3-368A4.5 | -0.02008781 | 0.061059 | -0.32899 | 0.742164 | 0.903718 |
| RP4-639F20.1 | 0.02351912 | 0.071648 | 0.328257 | 0.742717 | 0.903718 |
| TSTD3 | -0.027432717 | 0.083361 | -0.32908 | 0.742094 | 0.903718 |
| RP11-111F5.4 | -0.029799123 | 0.090938 | -0.32769 | 0.743149 | 0.903766 |
| RP11-484O2.1 | -0.02810141 | 0.085895 | -0.32716 | 0.743546 | 0.903773 |
| RP11-380G5.2 | 0.031287035 | 0.095884 | 0.326302 | 0.744196 | 0.904086 |
| CTC-471J1.2 | 0.029556455 | 0.0908 | 0.325513 | 0.744793 | 0.904335 |
| AC009961.3 | 0.021314687 | 0.065747 | 0.324191 | 0.745793 | 0.90503 |
| RP11-24B19.4 | 0.033063339 | 0.102299 | 0.323202 | 0.746542 | 0.90503 |
| RP11-6O2.3 | 0.02930383 | 0.090561 | 0.323583 | 0.746254 | 0.90503 |
| AP000487.5 | -0.027655784 | 0.086333 | -0.32034 | 0.748712 | 0.907014 |
| MMP24-AS1 | 0.023801818 | 0.07454 | 0.319316 | 0.749487 | 0.907014 |
| RP11-480A16.1 | 0.024478603 | 0.076703 | 0.319135 | 0.749624 | 0.907014 |
| RP5-1160K1.3 | -0.028747405 | 0.090126 | -0.31897 | 0.749751 | 0.907014 |
| AC092415.1 | 0.028545677 | 0.089889 | 0.317566 | 0.750814 | 0.907824 |
| CTC-338M12.5 | 0.030721073 | 0.097365 | 0.315524 | 0.752364 | 0.908347 |
| LINC00391 | -0.040023089 | 0.127025 | -0.31508 | 0.752701 | 0.908347 |
| RP11-120K9.2 | 0.017399361 | 0.055285 | 0.314723 | 0.752972 | 0.908347 |
| RP4-622L5.7 | 0.023457907 | 0.074611 | 0.314402 | 0.753215 | 0.908347 |
| RP5-968J1.1 | -0.030334039 | 0.096174 | -0.31541 | 0.752451 | 0.908347 |
| RP11-18I14.10 | 0.018645531 | 0.059612 | 0.312783 | 0.754445 | 0.90888 |
| RP11-29P20.1 | 0.036764887 | 0.117527 | 0.31282 | 0.754418 | 0.90888 |
| RP11-521I2.3 | -0.031424346 | 0.100783 | -0.3118 | 0.755191 | 0.909303 |
| RP1-232L24.3 | 0.035770488 | 0.114988 | 0.31108 | 0.75574 | 0.90949 |
| C14orf169 | 0.019554731 | 0.06326 | 0.309119 | 0.757231 | 0.909655 |
| LINC00910 | -0.03103271 | 0.10001 | -0.3103 | 0.756335 | 0.909655 |
| RP11-389C8.2 | -0.04017939 | 0.130104 | -0.30883 | 0.757454 | 0.909655 |
| RP11-50E11.3 | 0.023402459 | 0.075562 | 0.309711 | 0.75678 | 0.909655 |
| AJ006998.2 | -0.038916156 | 0.128038 | -0.30394 | 0.761171 | 0.913643 |
| CTC-325H20.2 | -0.034911958 | 0.115086 | -0.30336 | 0.761619 | 0.913705 |
| RP13-514E23.1 | 0.027544874 | 0.091028 | 0.302596 | 0.762197 | 0.913924 |
| AC009506.1 | 0.026806963 | 0.089095 | 0.300879 | 0.763507 | 0.914429 |
| CTA-29F11.1 | 0.033761034 | 0.112581 | 0.299882 | 0.764267 | 0.914429 |
| LINC00176 | -0.025648805 | 0.085547 | -0.29982 | 0.764315 | 0.914429 |
| RP11-313C15.1 | 0.035551744 | 0.118932 | 0.298925 | 0.764997 | 0.914429 |
| RP3-428L16.2 | -0.02985751 | 0.099779 | -0.29924 | 0.764759 | 0.914429 |
| RP6-99M1.3 | -0.037783694 | 0.126183 | -0.29944 | 0.764607 | 0.914429 |
| CTD-3138B18.6 | -0.026963098 | 0.090535 | -0.29782 | 0.76584 | 0.914963 |
| RBM26-AS1 | -0.025839742 | 0.086937 | -0.29723 | 0.766295 | 0.915032 |
| CTD-2626G11.2 | -0.032395552 | 0.109249 | -0.29653 | 0.766826 | 0.915193 |
| ASH1L-AS1 | 0.022699857 | 0.077451 | 0.293088 | 0.769455 | 0.917855 |
| MAPKAPK5-AS1 | 0.02475531 | 0.084988 | 0.291281 | 0.770837 | 0.918926 |
| SEC22B | -0.016765623 | 0.057639 | -0.29087 | 0.771149 | 0.918926 |
| THRB-IT1 | 0.023546754 | 0.081649 | 0.288389 | 0.773049 | 0.920715 |
| FGF14-IT1 | -0.025684907 | 0.089922 | -0.28563 | 0.775158 | 0.921919 |
| RP11-295K2.3 | -0.027592501 | 0.096659 | -0.28546 | 0.775291 | 0.921919 |
| RP11-307E17.8 | -0.018164803 | 0.063512 | -0.28601 | 0.774873 | 0.921919 |
| RP11-547I7.2 | -0.029109027 | 0.102144 | -0.28498 | 0.775659 | 0.921919 |
| RP11-145G20.1 | 0.025554277 | 0.090332 | 0.282892 | 0.77726 | 0.923346 |
| RP11-701H24.7 | 0.019804242 | 0.070222 | 0.282022 | 0.777926 | 0.923662 |
| GRIP2 | 0.028695238 | 0.101951 | 0.281461 | 0.778357 | 0.923667 |
| LINC00205 | 0.032165716 | 0.114551 | 0.280799 | 0.778865 | 0.923667 |
| RP11-111M22.3 | 0.026430767 | 0.094351 | 0.280133 | 0.779376 | 0.923667 |
| RP11-473I1.9 | -0.018662358 | 0.066668 | -0.27993 | 0.779531 | 0.923667 |
| CTD-2044J15.2 | -0.018188781 | 0.065176 | -0.27907 | 0.78019 | 0.923972 |
| AL136419.6 | -0.023715662 | 0.08564 | -0.27692 | 0.781839 | 0.924501 |
| FAM155A-IT1 | 0.026123184 | 0.094233 | 0.277219 | 0.781612 | 0.924501 |
| RP11-46H11.3 | 0.027586494 | 0.099597 | 0.276981 | 0.781795 | 0.924501 |
| RP11-314D7.2 | -0.023921271 | 0.086932 | -0.27517 | 0.783185 | 0.925144 |
| RP11-31F15.1 | 0.019480048 | 0.070706 | 0.275507 | 0.782927 | 0.925144 |
| RP11-1094M14.11 | -0.021799566 | 0.0796 | -0.27386 | 0.78419 | 0.925384 |
| RP11-54O7.14 | 0.02208376 | 0.080554 | 0.27415 | 0.783969 | 0.925384 |
| RP11-415C15.2 | 0.029593414 | 0.108307 | 0.273238 | 0.784671 | 0.925478 |
| AC002398.5 | 0.029748776 | 0.10939 | 0.271951 | 0.785659 | 0.925524 |
| RP11-150O12.1 | 0.036558867 | 0.134252 | 0.272315 | 0.78538 | 0.925524 |
| RP11-611L7.1 | -0.016733039 | 0.061723 | -0.2711 | 0.786314 | 0.925524 |
| TEN1-CDK3 | 0.022353694 | 0.082315 | 0.271563 | 0.785958 | 0.925524 |
| AC007405.6 | -0.023604391 | 0.087776 | -0.26892 | 0.787995 | 0.9261 |
| CTC-340A15.2 | -0.019502809 | 0.072622 | -0.26855 | 0.788274 | 0.9261 |
| EAF1-AS1 | -0.016171426 | 0.060607 | -0.26683 | 0.789603 | 0.9261 |
| RP11-165J3.6 | 0.028598105 | 0.107184 | 0.266813 | 0.789613 | 0.9261 |
| RP11-385D13.3 | -0.021538487 | 0.080561 | -0.26736 | 0.789196 | 0.9261 |
| RP11-67L2.2 | 0.021187736 | 0.079375 | 0.266931 | 0.789523 | 0.9261 |
| RP11-782C8.2 | -0.035719876 | 0.132764 | -0.26905 | 0.787893 | 0.9261 |
| MBNL1-AS1 | -0.021643498 | 0.081547 | -0.26541 | 0.790693 | 0.92643 |
| RP11-88L24.4 | -0.028288937 | 0.106588 | -0.2654 | 0.790698 | 0.92643 |
| RP11-171I2.3 | -0.029055725 | 0.110112 | -0.26387 | 0.791877 | 0.92734 |
| AC009052.12 | -0.017619651 | 0.068118 | -0.25866 | 0.795894 | 0.927595 |
| PINK1-AS | 0.01776482 | 0.068159 | 0.260637 | 0.794373 | 0.927595 |
| RP11-142G1.3 | -0.020555081 | 0.07841 | -0.26215 | 0.793206 | 0.927595 |
| RP11-247L20.4 | 0.025665117 | 0.099332 | 0.258378 | 0.796115 | 0.927595 |
| RP11-282O18.6 | 0.020118588 | 0.076956 | 0.26143 | 0.793761 | 0.927595 |
| RP11-372K14.2 | 0.030179169 | 0.116547 | 0.258944 | 0.795679 | 0.927595 |
| RP11-527F13.1 | 0.028566027 | 0.109627 | 0.260575 | 0.79442 | 0.927595 |
| RP13-516M14.1 | -0.021998877 | 0.084052 | -0.26173 | 0.793529 | 0.927595 |
| SCARNA17 | -0.020781565 | 0.080283 | -0.25885 | 0.795749 | 0.927595 |
| SNORA71B | 0.02753481 | 0.106142 | 0.259415 | 0.795315 | 0.927595 |
| RP11-727A23.5 | 0.01903321 | 0.073833 | 0.257787 | 0.796571 | 0.927658 |
| CTD-2192J16.21 | -0.024836913 | 0.097824 | -0.25389 | 0.799577 | 0.930688 |
| CTD-2245F17.3 | -0.027347792 | 0.109433 | -0.24991 | 0.80266 | 0.930898 |
| CTD-2313N18.5 | 0.018938266 | 0.075898 | 0.249522 | 0.802957 | 0.930898 |
| RP1-90G24.10 | 0.03214818 | 0.128307 | 0.250556 | 0.802157 | 0.930898 |
| RP11-259O2.1 | 0.025835397 | 0.103385 | 0.249896 | 0.802668 | 0.930898 |
| RP11-52A20.2 | 0.024762942 | 0.098544 | 0.251287 | 0.801592 | 0.930898 |
| RP11-552M11.8 | 0.019967934 | 0.079798 | 0.250232 | 0.802408 | 0.930898 |
| RP11-600F24.7 | 0.019782126 | 0.079458 | 0.248964 | 0.803388 | 0.930898 |
| RP11-77K12.8 | -0.014158943 | 0.056729 | -0.24959 | 0.802904 | 0.930898 |
| RP11-981G7.1 | 0.025325902 | 0.101669 | 0.249101 | 0.803283 | 0.930898 |
| GS1-251I9.4 | -0.015966954 | 0.064821 | -0.24632 | 0.805433 | 0.930929 |
| RP11-334C17.5 | 0.023162073 | 0.093692 | 0.247214 | 0.804742 | 0.930929 |
| RP4-622L5.2 | 0.015609479 | 0.063277 | 0.246685 | 0.805152 | 0.930929 |
| RP5-1136G13.2 | 0.021872288 | 0.088744 | 0.246464 | 0.805323 | 0.930929 |
| SCARNA9 | -0.02184381 | 0.088003 | -0.24822 | 0.803967 | 0.930929 |
| CTD-3096M3.2 | 0.027117107 | 0.110407 | 0.24561 | 0.805984 | 0.9311 |
| RP11-458D21.1 | 0.021020344 | 0.08579 | 0.245021 | 0.80644 | 0.93116 |
| RP11-305E17.6 | -0.0243423 | 0.099695 | -0.24417 | 0.807102 | 0.931458 |
| AGBL4-IT1 | 0.027820437 | 0.115197 | 0.241503 | 0.809165 | 0.932264 |
| LA16c-306A4.2 | -0.024221371 | 0.100647 | -0.24066 | 0.809821 | 0.932264 |
| RNU12 | -0.02802993 | 0.115798 | -0.24206 | 0.808735 | 0.932264 |
| RP11-309L24.9 | -0.023518113 | 0.097619 | -0.24092 | 0.809619 | 0.932264 |
| RP11-509E16.1 | -0.016801591 | 0.069348 | -0.24228 | 0.808565 | 0.932264 |
| RP11-19N8.4 | -0.028800375 | 0.12074 | -0.23853 | 0.811468 | 0.933229 |
| RP11-297M9.2 | -0.024277719 | 0.101669 | -0.23879 | 0.811267 | 0.933229 |
| AC084219.4 | -0.019878282 | 0.084897 | -0.23414 | 0.814873 | 0.933812 |
| RP1-151F17.2 | -0.021335457 | 0.091292 | -0.23371 | 0.815213 | 0.933812 |
| RP1-90G24.6 | 0.030972635 | 0.131759 | 0.235071 | 0.814154 | 0.933812 |
| RP11-274B21.10 | 0.025528054 | 0.10907 | 0.234053 | 0.814944 | 0.933812 |
| RP11-434H6.7 | 0.020584516 | 0.08723 | 0.23598 | 0.813448 | 0.933812 |
| RP11-54A4.2 | 0.02271755 | 0.096315 | 0.235866 | 0.813537 | 0.933812 |
| RP4-545K15.5 | -0.0214567 | 0.091591 | -0.23427 | 0.814779 | 0.933812 |
| RP4-740C4.7 | 0.024268602 | 0.103129 | 0.235324 | 0.813958 | 0.933812 |
| AC007228.11 | 0.023559759 | 0.101764 | 0.231513 | 0.816916 | 0.934018 |
| AP006621.6 | -0.024310993 | 0.104886 | -0.23179 | 0.816704 | 0.934018 |
| RP11-510H23.1 | -0.020872086 | 0.089702 | -0.23268 | 0.816007 | 0.934018 |
| SNORD64 | -0.013633765 | 0.058921 | -0.23139 | 0.817012 | 0.934018 |
| RP11-24J23.2 | -0.027338465 | 0.118623 | -0.23047 | 0.81773 | 0.934375 |
| CTC-429P9.3 | -0.012712846 | 0.055421 | -0.22939 | 0.818569 | 0.934871 |
| RP11-353N4.1 | 0.028130079 | 0.122959 | 0.228776 | 0.819043 | 0.934949 |
| CTC-559E9.4 | -0.01795874 | 0.07965 | -0.22547 | 0.821613 | 0.937418 |
| AC139100.3 | 0.027894572 | 0.125822 | 0.221698 | 0.824549 | 0.940304 |
| RP11-7O11.3 | -0.023325804 | 0.105562 | -0.22097 | 0.825118 | 0.940487 |
| AC005013.5 | -0.026053705 | 0.118865 | -0.21919 | 0.826504 | 0.940771 |
| RP11-1017G21.5 | -0.019172125 | 0.087513 | -0.21908 | 0.82659 | 0.940771 |
| RP11-20I23.13 | 0.020740048 | 0.094497 | 0.219477 | 0.826278 | 0.940771 |
| RP11-352M15.1 | -0.025149901 | 0.115137 | -0.21843 | 0.827091 | 0.940877 |
| RP11-244O19.1 | 0.01596087 | 0.073993 | 0.215706 | 0.829217 | 0.942554 |
| RP5-882C2.2 | -0.019682299 | 0.091418 | -0.2153 | 0.829533 | 0.942554 |
| RP6-99M1.2 | 0.024475203 | 0.113854 | 0.21497 | 0.829791 | 0.942554 |
| AC009120.6 | -0.017957242 | 0.084591 | -0.21228 | 0.831887 | 0.942573 |
| AC091814.3 | 0.017788293 | 0.084026 | 0.2117 | 0.832341 | 0.942573 |
| FAM66B | 0.018106548 | 0.085204 | 0.212509 | 0.83171 | 0.942573 |
| RP11-1143G9.4 | 0.019113246 | 0.090688 | 0.210758 | 0.833076 | 0.942573 |
| RP11-252A24.3 | 0.023402376 | 0.110616 | 0.211564 | 0.832447 | 0.942573 |
| RP11-629O1.2 | -0.020109506 | 0.095283 | -0.21105 | 0.832848 | 0.942573 |
| RP11-886D15.1 | -0.026447303 | 0.125124 | -0.21137 | 0.8326 | 0.942573 |
| SNHG17 | 0.017741252 | 0.084077 | 0.211011 | 0.832879 | 0.942573 |
| CTC-365E16.1 | -0.012761099 | 0.060865 | -0.20966 | 0.833931 | 0.943078 |
| CTA-445C9.15 | 0.024710268 | 0.119476 | 0.206823 | 0.836148 | 0.943274 |
| DIO3OS | 0.023593183 | 0.113375 | 0.208099 | 0.835152 | 0.943274 |
| RP1-130H16.16 | 0.018087707 | 0.08686 | 0.208241 | 0.835041 | 0.943274 |
| RP11-266O8.1 | -0.018978728 | 0.091375 | -0.2077 | 0.835461 | 0.943274 |
| RP11-307O13.1 | -0.02446186 | 0.118193 | -0.20697 | 0.836037 | 0.943274 |
| RP11-532F6.4 | 0.019687827 | 0.095515 | 0.206123 | 0.836695 | 0.943429 |
| CTD-2651B20.1 | 0.018256559 | 0.088967 | 0.205206 | 0.837412 | 0.943775 |
| RP11-13N13.2 | -0.013706806 | 0.067074 | -0.20435 | 0.838078 | 0.944066 |
| RP11-22H5.2 | 0.021897476 | 0.107521 | 0.203657 | 0.838622 | 0.944217 |
| RP11-158H5.7 | -0.014829386 | 0.0731 | -0.20286 | 0.839241 | 0.944453 |
| MIR4458HG | 0.0187387 | 0.093096 | 0.201284 | 0.840476 | 0.944922 |
| RP11-89K21.1 | -0.0276192 | 0.137162 | -0.20136 | 0.840416 | 0.944922 |
| RP11-258C19.7 | -0.011873444 | 0.059461 | -0.19969 | 0.841727 | 0.945622 |
| RP11-793H13.11 | 0.019610532 | 0.098461 | 0.199171 | 0.842129 | 0.945622 |
| RP4-758J18.13 | -0.012257717 | 0.061623 | -0.19892 | 0.842329 | 0.945622 |
| CTD-2162K18.5 | 0.012862936 | 0.065799 | 0.195489 | 0.84501 | 0.94587 |
| RP11-384K6.6 | 0.016244596 | 0.082278 | 0.197437 | 0.843486 | 0.94587 |
| RP11-517C16.2 | -0.021225033 | 0.108573 | -0.19549 | 0.845009 | 0.94587 |
| RP11-713C5.1 | -0.016948948 | 0.085934 | -0.19723 | 0.843645 | 0.94587 |
| THAP7-AS1 | -0.021609941 | 0.110454 | -0.19565 | 0.844887 | 0.94587 |
| hsa-mir-6080 | 0.013002164 | 0.066159 | 0.196529 | 0.844196 | 0.94587 |
| CTD-2619J13.8 | -0.016843319 | 0.087328 | -0.19288 | 0.847057 | 0.946823 |
| DAPK1-IT1 | -0.01959452 | 0.101616 | -0.19283 | 0.847093 | 0.946823 |
| RP11-392E22.10 | 0.023302006 | 0.120285 | 0.193723 | 0.846393 | 0.946823 |
| AC074117.10 | 0.012006053 | 0.063454 | 0.18921 | 0.849928 | 0.947697 |
| RP11-530N7.3 | -0.017859998 | 0.09412 | -0.18976 | 0.849498 | 0.947697 |
| RP11-815I9.4 | -0.018230668 | 0.096055 | -0.18979 | 0.849471 | 0.947697 |
| SNHG8 | 0.019174613 | 0.101095 | 0.189669 | 0.849569 | 0.947697 |
| ZNF718 | 0.012769552 | 0.067111 | 0.190275 | 0.849093 | 0.947697 |
| RP4-548D19.3 | 0.016439236 | 0.087645 | 0.187565 | 0.851217 | 0.948676 |
| CTB-13L3.1 | 0.014671038 | 0.078584 | 0.186693 | 0.851902 | 0.94898 |
| RP11-544I20.2 | -0.015656211 | 0.084884 | -0.18444 | 0.853667 | 0.950487 |
| RP13-36G14.4 | 0.017923734 | 0.097516 | 0.183803 | 0.854168 | 0.950586 |
| CASP8AP2 | -0.009535025 | 0.052327 | -0.18222 | 0.855411 | 0.950902 |
| CTC-459I6.1 | -0.018885847 | 0.103521 | -0.18244 | 0.855241 | 0.950902 |
| CTD-2269F5.1 | -0.016857378 | 0.092952 | -0.18136 | 0.856089 | 0.950902 |
| RP11-819C21.1 | 0.0122189 | 0.067381 | 0.181341 | 0.8561 | 0.950902 |
| CTD-2037K23.2 | 0.015674516 | 0.088957 | 0.176204 | 0.860134 | 0.951871 |
| LINC00355 | 0.015450482 | 0.086723 | 0.178159 | 0.858598 | 0.951871 |
| LINC00403 | -0.016167767 | 0.091049 | -0.17757 | 0.859059 | 0.951871 |
| MYCBP2-AS2 | -0.018024853 | 0.101084 | -0.17832 | 0.858475 | 0.951871 |
| RP11-229C3.2 | -0.022677989 | 0.129389 | -0.17527 | 0.860868 | 0.951871 |
| RP11-386G11.10 | 0.017273989 | 0.098581 | 0.175227 | 0.860901 | 0.951871 |
| RP11-506N2.1 | 0.019028545 | 0.107563 | 0.176906 | 0.859582 | 0.951871 |
| RP11-679C8.2 | -0.017757875 | 0.09961 | -0.17827 | 0.858507 | 0.951871 |
| RP11-807G9.2 | 0.014928026 | 0.08583 | 0.173926 | 0.861924 | 0.951871 |
| RP5-1142A6.2 | -0.023270148 | 0.13278 | -0.17525 | 0.86088 | 0.951871 |
| RP5-991G20.4 | -0.013800164 | 0.079323 | -0.17397 | 0.861885 | 0.951871 |
| TOB1-AS1 | 0.013724321 | 0.078506 | 0.174819 | 0.861222 | 0.951871 |
| RP11-403B2.7 | -0.023114626 | 0.1339 | -0.17263 | 0.862946 | 0.952216 |
| RP11-431I8.1 | 0.018131469 | 0.105123 | 0.172478 | 0.863061 | 0.952216 |
| AC006262.6 | -0.015238131 | 0.089051 | -0.17112 | 0.864131 | 0.95294 |
| RP3-325F22.5 | 0.014421253 | 0.084697 | 0.170268 | 0.8648 | 0.953221 |
| MIR4519 | -0.016660411 | 0.098393 | -0.16933 | 0.86554 | 0.95323 |
| RP11-101E13.5 | -0.015139086 | 0.089498 | -0.16915 | 0.865675 | 0.95323 |
| RP5-1139I1.1 | -0.013751979 | 0.081526 | -0.16868 | 0.866047 | 0.95323 |
| RP11-588P7.1 | -0.018897284 | 0.112641 | -0.16776 | 0.866768 | 0.953331 |
| RP13-582O9.5 | 0.018452414 | 0.110154 | 0.167515 | 0.866965 | 0.953331 |
| RP11-119D9.1 | -0.016419414 | 0.098762 | -0.16625 | 0.867958 | 0.953514 |
| TTLL11-IT1 | 0.016037556 | 0.096382 | 0.166396 | 0.867845 | 0.953514 |
| RP11-1055B8.4 | -0.020845724 | 0.127144 | -0.16395 | 0.869768 | 0.954593 |
| RP11-708J19.1 | 0.014330646 | 0.087318 | 0.164121 | 0.869636 | 0.954593 |
| CTC-228N24.3 | -0.008311442 | 0.051544 | -0.16125 | 0.871897 | 0.954658 |
| RP11-33A14.1 | -0.021682002 | 0.134057 | -0.16174 | 0.871513 | 0.954658 |
| RP11-403I13.8 | 0.015052654 | 0.093083 | 0.161711 | 0.871533 | 0.954658 |
| RP3-405J10.3 | -0.010899716 | 0.067094 | -0.16245 | 0.870948 | 0.954658 |
| TTC28-AS1 | -0.00936527 | 0.057939 | -0.16164 | 0.871589 | 0.954658 |
| CTC-429P9.5 | -0.0102747 | 0.065347 | -0.15723 | 0.875062 | 0.957215 |
| RP3-525N10.2 | -0.00827768 | 0.052608 | -0.15735 | 0.874972 | 0.957215 |
| RP11-881M11.4 | -0.016596022 | 0.106174 | -0.15631 | 0.875789 | 0.957557 |
| RP11-452L6.5 | 0.008432584 | 0.054925 | 0.153528 | 0.877982 | 0.959243 |
| RP11-680F20.11 | 0.013858877 | 0.09046 | 0.153204 | 0.878237 | 0.959243 |
| SSPO | 0.015689866 | 0.102701 | 0.152772 | 0.878578 | 0.959243 |
| CTC-513N18.7 | 0.010362079 | 0.068654 | 0.150932 | 0.880029 | 0.959918 |
| PXN-AS1 | -0.014339603 | 0.09472 | -0.15139 | 0.879668 | 0.959918 |
| RP11-673E1.4 | -0.015553815 | 0.103598 | -0.15014 | 0.880657 | 0.960149 |
| CTD-2651B20.3 | 0.019918866 | 0.133304 | 0.149425 | 0.881218 | 0.960308 |
| AP001597.1 | 0.014850591 | 0.101421 | 0.146426 | 0.883585 | 0.962369 |
| RP5-1043L13.1 | -0.018084291 | 0.12389 | -0.14597 | 0.883945 | 0.962369 |
| KCNQ5-IT1 | 0.017161689 | 0.119144 | 0.144042 | 0.885468 | 0.963029 |
| RP11-111K18.2 | 0.013361033 | 0.093032 | 0.143617 | 0.885803 | 0.963029 |
| SPATA41 | 0.013904157 | 0.096408 | 0.144221 | 0.885326 | 0.963029 |
| AC005754.8 | -0.013594388 | 0.095354 | -0.14257 | 0.886632 | 0.963071 |
| CTD-2298J14.2 | 0.01579565 | 0.111183 | 0.142069 | 0.887025 | 0.963071 |
| RP11-425I13.3 | -0.013328996 | 0.093878 | -0.14198 | 0.887094 | 0.963071 |
| RP1-266L20.9 | 0.01461733 | 0.103896 | 0.140692 | 0.888113 | 0.963724 |
| LINC00963 | 0.008481205 | 0.060669 | 0.139795 | 0.888822 | 0.96404 |
| RP11-123O10.4 | 0.013228645 | 0.095847 | 0.138018 | 0.890226 | 0.964655 |
| RP11-227G15.9 | -0.014203498 | 0.102884 | -0.13805 | 0.890198 | 0.964655 |
| AP000525.9 | -0.014864837 | 0.108539 | -0.13695 | 0.891067 | 0.964661 |
| RP11-863P13.6 | 0.015157701 | 0.11055 | 0.137111 | 0.890943 | 0.964661 |
| RP11-139H15.1 | -0.009635759 | 0.071675 | -0.13444 | 0.893057 | 0.965782 |
| RP11-166B2.3 | -0.012214368 | 0.091114 | -0.13406 | 0.893359 | 0.965782 |
| RP11-554F20.1 | -0.012386135 | 0.092325 | -0.13416 | 0.893278 | 0.965782 |
| RP4-564M11.2 | 0.013497064 | 0.10143 | 0.133068 | 0.89414 | 0.966173 |
| C1orf132 | -0.009503719 | 0.072257 | -0.13153 | 0.895358 | 0.967037 |
| RP11-501E14.1 | -0.015414615 | 0.120276 | -0.12816 | 0.898022 | 0.96946 |
| LEPREL1-AS1 | -0.012575827 | 0.099151 | -0.12683 | 0.899071 | 0.970139 |
| AC093391.2 | -0.011629102 | 0.092614 | -0.12557 | 0.900076 | 0.970769 |
| RP13-439H18.4 | 0.012829641 | 0.103383 | 0.124098 | 0.901238 | 0.971114 |
| WDR11-AS1 | -0.01045255 | 0.083988 | -0.12445 | 0.900956 | 0.971114 |
| RP13-941N14.1 | -0.011559912 | 0.093707 | -0.12336 | 0.90182 | 0.971288 |
| AC005518.2 | -0.010568133 | 0.093476 | -0.11306 | 0.909985 | 0.972016 |
| AC005562.1 | 0.006516718 | 0.054562 | 0.119437 | 0.904929 | 0.972016 |
| AC017048.3 | 0.012653602 | 0.109708 | 0.115339 | 0.908177 | 0.972016 |
| AC092661.1 | 0.014841698 | 0.124014 | 0.119677 | 0.904739 | 0.972016 |
| AGAP1-IT1 | 0.010561664 | 0.09429 | 0.112012 | 0.910814 | 0.972016 |
| ATP6V0E2-AS1 | 0.008774861 | 0.074953 | 0.117071 | 0.906804 | 0.972016 |
| CTD-2323K18.1 | 0.007309244 | 0.063738 | 0.114675 | 0.908702 | 0.972016 |
| HCG22 | 0.013312369 | 0.118051 | 0.112768 | 0.910214 | 0.972016 |
| KB-431C1.4 | -0.010042126 | 0.088382 | -0.11362 | 0.909537 | 0.972016 |
| LIFR-AS1 | 0.007690816 | 0.064606 | 0.119042 | 0.905242 | 0.972016 |
| LINC00571 | 0.011890853 | 0.102837 | 0.115628 | 0.907947 | 0.972016 |
| MAPT-AS1 | 0.013552971 | 0.111284 | 0.121787 | 0.903067 | 0.972016 |
| RP11-102L12.2 | -0.011072688 | 0.096622 | -0.1146 | 0.908764 | 0.972016 |
| RP11-166O4.5 | 0.010552467 | 0.09 | 0.117249 | 0.906663 | 0.972016 |
| RP11-16P6.1 | -0.010149956 | 0.089705 | -0.11315 | 0.909913 | 0.972016 |
| RP11-227G15.3 | -0.008081367 | 0.068118 | -0.11864 | 0.905562 | 0.972016 |
| RP11-262H14.3 | 0.015773178 | 0.134526 | 0.11725 | 0.906662 | 0.972016 |
| RP11-264B17.3 | -0.007098608 | 0.061888 | -0.1147 | 0.908682 | 0.972016 |
| RP11-32B11.2 | -0.009466625 | 0.081138 | -0.11667 | 0.907119 | 0.972016 |
| RP11-638F5.1 | 0.015291029 | 0.13668 | 0.111874 | 0.910923 | 0.972016 |
| CTD-2410N18.4 | 0.00798279 | 0.072745 | 0.109737 | 0.912618 | 0.973375 |
| GHRLOS | -0.011601802 | 0.106546 | -0.10889 | 0.913289 | 0.973641 |
| KCNH1-IT1 | 0.012530665 | 0.118366 | 0.105863 | 0.915691 | 0.97555 |
| RP1-86D1.2 | 0.011219613 | 0.107106 | 0.104753 | 0.916572 | 0.97555 |
| RP11-468H14.2 | 0.010922769 | 0.103557 | 0.105476 | 0.915998 | 0.97555 |
| RP11-574K11.29 | -0.008233993 | 0.078793 | -0.1045 | 0.916771 | 0.97555 |
| RP11-356B19.11 | -0.008834421 | 0.088526 | -0.09979 | 0.920508 | 0.979074 |
| USP46-AS1 | -0.008808139 | 0.088952 | -0.09902 | 0.921122 | 0.979275 |
| CTD-2366F13.1 | 0.008111837 | 0.083455 | 0.0972 | 0.922567 | 0.979964 |
| MIR940 | 0.008794005 | 0.091368 | 0.096248 | 0.923324 | 0.979964 |
| RP11-212P7.3 | -0.007627236 | 0.080158 | -0.09515 | 0.924194 | 0.979964 |
| RP11-321E2.3 | 0.00956974 | 0.098719 | 0.096939 | 0.922775 | 0.979964 |
| RP11-378A13.1 | 0.009760276 | 0.10246 | 0.09526 | 0.924109 | 0.979964 |
| ZNF761 | 0.007006958 | 0.073761 | 0.094995 | 0.924318 | 0.979964 |
| AC005062.2 | -0.010531535 | 0.11579 | -0.09095 | 0.927529 | 0.982361 |
| CTD-2006C1.2 | -0.007873881 | 0.086651 | -0.09087 | 0.927597 | 0.982361 |
| RP11-382A20.2 | -0.009151638 | 0.101076 | -0.09054 | 0.927856 | 0.982361 |
| LINC00493 | 0.007266585 | 0.081686 | 0.088957 | 0.929116 | 0.983097 |
| UBL7-AS1 | 0.00758087 | 0.085568 | 0.088595 | 0.929404 | 0.983097 |
| AC012358.7 | -0.009485325 | 0.108932 | -0.08708 | 0.930611 | 0.983923 |
| PWAR6 | -0.006265593 | 0.073076 | -0.08574 | 0.931673 | 0.984594 |
| RP11-309L24.4 | -0.008893205 | 0.105809 | -0.08405 | 0.933017 | 0.984661 |
| RP11-404O13.5 | -0.007666015 | 0.0904 | -0.0848 | 0.93242 | 0.984661 |
| RP11-518L10.5 | 0.006259237 | 0.074222 | 0.084332 | 0.932793 | 0.984661 |
| RP11-781A6.1 | -0.008779963 | 0.105873 | -0.08293 | 0.933908 | 0.985151 |
| AC097662.2 | -0.005652774 | 0.072442 | -0.07803 | 0.937803 | 0.985922 |
| LINC00473 | -0.009172196 | 0.118029 | -0.07771 | 0.938058 | 0.985922 |
| RP11-158G18.1 | -0.008545205 | 0.109302 | -0.07818 | 0.937685 | 0.985922 |
| RP11-171I2.4 | -0.008800865 | 0.10806 | -0.08144 | 0.935089 | 0.985922 |
| RP11-214K3.24 | -0.007014464 | 0.089651 | -0.07824 | 0.937636 | 0.985922 |
| RP11-321L2.2 | -0.007514803 | 0.094653 | -0.07939 | 0.93672 | 0.985922 |
| RP5-998N21.10 | 0.009768594 | 0.125308 | 0.077957 | 0.937862 | 0.985922 |
| ZSCAN16-AS1 | -0.00641539 | 0.079532 | -0.08066 | 0.935709 | 0.985922 |
| RP13-1032I1.7 | 0.006315594 | 0.081912 | 0.077102 | 0.938543 | 0.985983 |
| RP11-355O1.11 | 0.006000886 | 0.07954 | 0.075445 | 0.939861 | 0.986469 |
| RP11-428J1.4 | 0.007876307 | 0.103788 | 0.075888 | 0.939508 | 0.986469 |
| CTD-2369P2.2 | -0.006519151 | 0.088983 | -0.07326 | 0.941597 | 0.98702 |
| RP11-416N2.4 | -0.00811514 | 0.111726 | -0.07263 | 0.942097 | 0.98702 |
| RP11-478C19.2 | 0.005213175 | 0.071127 | 0.073293 | 0.941573 | 0.98702 |
| RP13-137A17.4 | -0.008241526 | 0.113095 | -0.07287 | 0.941907 | 0.98702 |
| CTD-2527I21.4 | 0.005068547 | 0.070557 | 0.071836 | 0.942732 | 0.987237 |
| FLVCR1-AS1 | 0.007173376 | 0.10148 | 0.070687 | 0.943647 | 0.987298 |
| RP11-436D23.1 | 0.008236482 | 0.116362 | 0.070783 | 0.94357 | 0.987298 |
| RP11-685M7.3 | -0.005896809 | 0.085659 | -0.06884 | 0.945116 | 0.988388 |
| RP11-111A21.1 | -0.006435839 | 0.095583 | -0.06733 | 0.946317 | 0.988589 |
| RP11-566E18.3 | 0.00523907 | 0.078214 | 0.066984 | 0.946594 | 0.988589 |
| RP11-989E6.10 | -0.008135222 | 0.12114 | -0.06716 | 0.946458 | 0.988589 |
| AP001429.1 | 0.006225572 | 0.096328 | 0.064629 | 0.948469 | 0.990099 |
| AC131097.3 | 0.006217517 | 0.097472 | 0.063788 | 0.949139 | 0.99035 |
| CTD-2165H16.4 | -0.005416627 | 0.090359 | -0.05995 | 0.952199 | 0.991922 |
| RP11-223I10.1 | 0.006444763 | 0.106769 | 0.060362 | 0.951867 | 0.991922 |
| RP11-251G23.5 | 0.005749361 | 0.093955 | 0.061193 | 0.951206 | 0.991922 |
| SNORD109A | 0.004362984 | 0.073037 | 0.059736 | 0.952366 | 0.991922 |
| AC022007.5 | -0.004828976 | 0.0839 | -0.05756 | 0.954102 | 0.992568 |
| CTD-3074O7.12 | -0.00428055 | 0.07392 | -0.05791 | 0.953822 | 0.992568 |
| RP11-317P15.5 | 0.006473327 | 0.112899 | 0.057337 | 0.954277 | 0.992568 |
| CTD-2058B24.2 | -0.004922985 | 0.097245 | -0.05062 | 0.959625 | 0.993181 |
| CTD-2619J13.14 | -0.003484514 | 0.065854 | -0.05291 | 0.957801 | 0.993181 |
| GABPB1-AS1 | -0.004870712 | 0.088805 | -0.05485 | 0.95626 | 0.993181 |
| LINC00265 | -0.003546043 | 0.070678 | -0.05017 | 0.959985 | 0.993181 |
| NDUFA6-AS1 | -0.003512025 | 0.070864 | -0.04956 | 0.960473 | 0.993181 |
| PDXDC2P | -0.003594009 | 0.068042 | -0.05282 | 0.957875 | 0.993181 |
| PTCHD3P1 | 0.002142343 | 0.040751 | 0.052572 | 0.958073 | 0.993181 |
| RP11-140H17.1 | 0.00326651 | 0.066618 | 0.049033 | 0.960893 | 0.993181 |
| RP11-182I10.3 | -0.004367887 | 0.083591 | -0.05225 | 0.958327 | 0.993181 |
| RP11-284F21.9 | -0.005621797 | 0.111284 | -0.05052 | 0.95971 | 0.993181 |
| RP11-362K14.5 | -0.005007685 | 0.100053 | -0.05005 | 0.960082 | 0.993181 |
| RP11-44F14.2 | -0.00552032 | 0.103918 | -0.05312 | 0.957635 | 0.993181 |
| RP11-686O6.2 | -0.005106901 | 0.096605 | -0.05286 | 0.957841 | 0.993181 |
| RP11-822E23.8 | 0.004947523 | 0.088719 | 0.055766 | 0.955528 | 0.993181 |
| INE1 | 0.00354758 | 0.075776 | 0.046817 | 0.962659 | 0.994561 |
| RP11-620J15.3 | 0.003575529 | 0.077564 | 0.046098 | 0.963232 | 0.99459 |
| TRIM52-AS1 | 0.004119627 | 0.090144 | 0.0457 | 0.963549 | 0.99459 |
| RP11-53B2.2 | -0.003330037 | 0.076482 | -0.04354 | 0.965271 | 0.995921 |
| AC004257.3 | 0.003973345 | 0.111818 | 0.035534 | 0.971654 | 0.996276 |
| AC092835.2 | -0.002196183 | 0.061161 | -0.03591 | 0.971355 | 0.996276 |
| ERVK13-1 | 0.002222938 | 0.059804 | 0.03717 | 0.970349 | 0.996276 |
| LINC00476 | -0.002250005 | 0.063211 | -0.03559 | 0.971605 | 0.996276 |
| MORC2-AS1 | -0.003736598 | 0.09765 | -0.03827 | 0.969476 | 0.996276 |
| RP11-120K24.4 | -0.00446004 | 0.10822 | -0.04121 | 0.967126 | 0.996276 |
| RP11-152N13.16 | -0.003043027 | 0.082432 | -0.03692 | 0.970552 | 0.996276 |
| RP11-218M22.1 | 0.003869491 | 0.093604 | 0.041339 | 0.967026 | 0.996276 |
| RP11-250B2.6 | 0.002991163 | 0.084197 | 0.035526 | 0.97166 | 0.996276 |
| RP11-372E1.6 | 0.003258771 | 0.079931 | 0.04077 | 0.967479 | 0.996276 |
| RP11-46A10.4 | 0.003939616 | 0.098765 | 0.039889 | 0.968182 | 0.996276 |
| RP11-776H12.1 | -0.003280543 | 0.087108 | -0.03766 | 0.969958 | 0.996276 |
| RP11-90D4.3 | -0.004438618 | 0.112373 | -0.0395 | 0.968493 | 0.996276 |
| XXbac-BPG300A18.13 | 0.00249583 | 0.070166 | 0.035571 | 0.971625 | 0.996276 |
| GS1-18A18.1 | -0.0035799 | 0.102429 | -0.03495 | 0.97212 | 0.996304 |
| AC002467.7 | -0.003643614 | 0.106139 | -0.03433 | 0.972615 | 0.996369 |
| AC005224.2 | 0.002854031 | 0.085846 | 0.033246 | 0.973478 | 0.996811 |
| AC004053.1 | 0.001915401 | 0.075914 | 0.025231 | 0.97987 | 0.99716 |
| AC004540.5 | 0.003091503 | 0.109389 | 0.028262 | 0.977453 | 0.99716 |
| AC068039.4 | -0.002280604 | 0.085045 | -0.02682 | 0.978606 | 0.99716 |
| AP001062.8 | -0.002726393 | 0.086805 | -0.03141 | 0.974944 | 0.99716 |
| CTC-218H9.1 | -0.003383838 | 0.117023 | -0.02892 | 0.976931 | 0.99716 |
| CTD-2541M15.4 | -0.002329763 | 0.08963 | -0.02599 | 0.979263 | 0.99716 |
| FAM85A | 0.00263917 | 0.094554 | 0.027912 | 0.977732 | 0.99716 |
| LA16c-306E5.3 | 0.00159465 | 0.06214 | 0.025662 | 0.979527 | 0.99716 |
| LINC00310 | 0.002882509 | 0.092308 | 0.031227 | 0.975088 | 0.99716 |
| RP11-196O16.1 | -0.002091469 | 0.074558 | -0.02805 | 0.977621 | 0.99716 |
| RP11-395I6.3 | 0.002398125 | 0.091737 | 0.026141 | 0.979145 | 0.99716 |
| RP11-747H7.3 | -0.003406825 | 0.110048 | -0.03096 | 0.975303 | 0.99716 |
| RP5-1112D6.7 | 0.002602324 | 0.091679 | 0.028385 | 0.977355 | 0.99716 |
| SNORD116-20 | 0.002696289 | 0.104089 | 0.025904 | 0.979334 | 0.99716 |
| AC074391.1 | -0.002017516 | 0.095641 | -0.02109 | 0.98317 | 0.997285 |
| CTD-2287O16.5 | -0.001994312 | 0.09306 | -0.02143 | 0.982902 | 0.997285 |
| RP11-10L7.1 | -0.002777428 | 0.136621 | -0.02033 | 0.983781 | 0.997285 |
| RP11-214K3.20 | -0.002069388 | 0.097486 | -0.02123 | 0.983064 | 0.997285 |
| RP11-296K13.4 | 0.00198669 | 0.092648 | 0.021443 | 0.982892 | 0.997285 |
| RP11-348P10.2 | 0.001703726 | 0.078412 | 0.021728 | 0.982665 | 0.997285 |
| RP11-65L3.1 | 0.001770172 | 0.09005 | 0.019658 | 0.984316 | 0.997285 |
| RP13-578N3.3 | -0.002520786 | 0.103722 | -0.0243 | 0.980611 | 0.997285 |
| RP3-329A5.8 | 0.001737007 | 0.078501 | 0.022127 | 0.982347 | 0.997285 |
| SOS1-IT1 | -0.001502872 | 0.074598 | -0.02015 | 0.983927 | 0.997285 |
| RP13-192B19.2 | 0.002262692 | 0.119758 | 0.018894 | 0.984926 | 0.997464 |
| RP11-242F4.2 | 0.001783599 | 0.097277 | 0.018335 | 0.985371 | 0.997478 |
| AC092192.1 | 0.001876356 | 0.127692 | 0.014694 | 0.988276 | 0.998246 |
| AC123886.2 | 0.001631881 | 0.102298 | 0.015952 | 0.987273 | 0.998246 |
| INHBA-AS1 | 0.00179768 | 0.121765 | 0.014764 | 0.988221 | 0.998246 |
| RP11-197M22.2 | -0.001766296 | 0.108561 | -0.01627 | 0.987019 | 0.998246 |
| RP11-687F6.1 | -0.001172698 | 0.079927 | -0.01467 | 0.988294 | 0.998246 |
| AC007038.7 | 4.57E-05 | 0.070989 | 0.000644 | 0.999486 | 0.999669 |
| AC009950.2 | 0.000345454 | 0.099181 | 0.003483 | 0.997221 | 0.999669 |
| AC073343.13 | 0.000662074 | 0.093257 | 0.007099 | 0.994335 | 0.999669 |
| AL589743.1 | -0.000788073 | 0.129306 | -0.00609 | 0.995137 | 0.999669 |
| AP000350.5 | 0.000672772 | 0.123969 | 0.005427 | 0.99567 | 0.999669 |
| CTA-228A9.3 | -0.001237312 | 0.101547 | -0.01218 | 0.990278 | 0.999669 |
| CTB-78F1.2 | -0.001316974 | 0.124312 | -0.01059 | 0.991547 | 0.999669 |
| CTD-3184A7.4 | -0.00048774 | 0.093044 | -0.00524 | 0.995817 | 0.999669 |
| FAM66A | 0.001128596 | 0.135704 | 0.008317 | 0.993364 | 0.999669 |
| LINC00984 | 0.000272361 | 0.06645 | 0.004099 | 0.99673 | 0.999669 |
| LINC01003 | 0.000700152 | 0.094795 | 0.007386 | 0.994107 | 0.999669 |
| PWRN1 | -0.000753734 | 0.0952 | -0.00792 | 0.993683 | 0.999669 |
| RP11-194N12.2 | -0.000315908 | 0.109586 | -0.00288 | 0.9977 | 0.999669 |
| RP11-25K19.1 | -0.00065678 | 0.085126 | -0.00772 | 0.993844 | 0.999669 |
| RP11-272P10.2 | -8.59E-05 | 0.079631 | -0.00108 | 0.999139 | 0.999669 |
| RP11-410L14.2 | -0.000194738 | 0.088943 | -0.00219 | 0.998253 | 0.999669 |
| RP11-514P8.8 | -0.000623945 | 0.123777 | -0.00504 | 0.995978 | 0.999669 |
| RP11-517B11.7 | -0.000717212 | 0.079036 | -0.00907 | 0.99276 | 0.999669 |
| RP11-574K11.28 | -0.00020893 | 0.086581 | -0.00241 | 0.998075 | 0.999669 |
| RP4-605O3.4 | -3.53E-05 | 0.085056 | -0.00042 | 0.999669 | 0.999669 |
| RPS6KA2-IT1 | -0.000327483 | 0.103149 | -0.00317 | 0.997467 | 0.999669 |
| STXBP5-AS1 | -0.000105786 | 0.094469 | -0.00112 | 0.999107 | 0.999669 |
| TBC1D3P1-DHX40P1 | -0.000178416 | 0.075899 | -0.00235 | 0.998124 | 0.999669 |
| AB015752.3 | -0.067204951 | 0.105326 | -0.63807 | 0.523431 | NA |
| ABO | 0.322489272 | 0.124934 | 2.581278 | 0.009844 | NA |
| AC000068.5 | 0.065333827 | 0.105201 | 0.621037 | 0.534575 | NA |
| AC002310.12 | -0.180022955 | 0.097563 | -1.84519 | 0.06501 | NA |
| AC002480.3 | -0.222255666 | 0.12334 | -1.80197 | 0.07155 | NA |
| AC003003.5 | 0.046815146 | 0.123988 | 0.377579 | 0.705743 | NA |
| AC003991.3 | 0.025463663 | 0.102937 | 0.247371 | 0.804621 | NA |
| AC004158.2 | -0.179025044 | 0.119035 | -1.50398 | 0.132588 | NA |
| AC004463.6 | 0.04928581 | 0.110653 | 0.445411 | 0.656023 | NA |
| AC005235.1 | -0.06117074 | 0.107965 | -0.56658 | 0.570999 | NA |
| AC005306.3 | -0.140530512 | 0.127158 | -1.10516 | 0.269088 | NA |
| AC005537.2 | 0.148960967 | 0.109153 | 1.364699 | 0.172348 | NA |
| AC007036.4 | -0.129776764 | 0.101537 | -1.27813 | 0.201204 | NA |
| AC007064.24 | -0.111324987 | 0.10355 | -1.07509 | 0.282335 | NA |
| AC007255.8 | 0.215685007 | 0.125463 | 1.719115 | 0.085593 | NA |
| AC007277.3 | -0.013357367 | 0.112905 | -0.11831 | 0.905825 | NA |
| AC007292.6 | 0.077317291 | 0.096872 | 0.798139 | 0.42479 | NA |
| AC007557.3 | -0.0039938 | 0.131266 | -0.03043 | 0.975728 | NA |
| AC007563.3 | -0.021293696 | 0.118426 | -0.17981 | 0.857305 | NA |
| AC008074.3 | 0.027215974 | 0.1089 | 0.249917 | 0.802652 | NA |
| AC009502.4 | 0.041029064 | 0.106812 | 0.384124 | 0.700886 | NA |
| AC010525.7 | -0.017969738 | 0.109923 | -0.16348 | 0.870144 | NA |
| AC010761.6 | -0.047302679 | 0.105948 | -0.44647 | 0.655256 | NA |
| AC015936.3 | -0.321547284 | 0.136991 | -2.34722 | 0.018914 | NA |
| AC017002.2 | 0.035321499 | 0.120301 | 0.293609 | 0.769056 | NA |
| AC023481.1 | -0.338866876 | 0.128282 | -2.64157 | 0.008252 | NA |
| AC062021.1 | 0.085469199 | 0.120708 | 0.708066 | 0.478904 | NA |
| AC068718.1 | 0.230904511 | 0.122542 | 1.884284 | 0.059527 | NA |
| AC069513.4 | 0.140868552 | 0.105893 | 1.330297 | 0.18342 | NA |
| AC073316.2 | 0.022659658 | 0.105293 | 0.215206 | 0.829606 | NA |
| AC073342.12 | -0.383036838 | 0.136856 | -2.79884 | 0.005129 | NA |
| AC092667.2 | 0.213229224 | 0.117083 | 1.821183 | 0.068579 | NA |
| AC093590.1 | -0.246815972 | 0.130755 | -1.88762 | 0.059077 | NA |
| AC095067.1 | -0.007526759 | 0.10566 | -0.07124 | 0.94321 | NA |
| AC099668.5 | 0.060950323 | 0.113499 | 0.537012 | 0.591259 | NA |
| AC099684.1 | -0.179560193 | 0.133287 | -1.34717 | 0.177926 | NA |
| AC099850.1 | 0.040678196 | 0.103366 | 0.393534 | 0.693925 | NA |
| AC104653.1 | 0.179776145 | 0.107381 | 1.674187 | 0.094094 | NA |
| AC104655.3 | 0.054635708 | 0.104859 | 0.521038 | 0.60234 | NA |
| AC108488.4 | -0.118507714 | 0.112127 | -1.05691 | 0.290554 | NA |
| AC113607.1 | 0.025857836 | 0.12085 | 0.213966 | 0.830574 | NA |
| AC114730.3 | -0.026471261 | 0.131051 | -0.20199 | 0.839924 | NA |
| AC115522.3 | -0.012278764 | 0.109995 | -0.11163 | 0.911117 | NA |
| AC144449.1 | 0.026033192 | 0.112209 | 0.232006 | 0.816533 | NA |
| AC144831.1 | 0.066367853 | 0.110021 | 0.603229 | 0.546357 | NA |
| AC226119.5 | 0.285519019 | 0.109978 | 2.596152 | 0.009427 | NA |
| ACVR2B-AS1 | -0.162775473 | 0.104119 | -1.56336 | 0.117968 | NA |
| AF064858.11 | 0.122142823 | 0.114498 | 1.066768 | 0.286077 | NA |
| AFAP1-AS1 | 0.196404812 | 0.131817 | 1.489986 | 0.136228 | NA |
| AJ239322.1 | 0.204596533 | 0.127491 | 1.604797 | 0.108538 | NA |
| AL121578.2 | 0.000901739 | 0.115213 | 0.007827 | 0.993755 | NA |
| ARHGEF7-IT1 | 0.250107271 | 0.11588 | 2.158336 | 0.030902 | NA |
| BACH1-IT1 | -0.198182732 | 0.113636 | -1.74402 | 0.081156 | NA |
| BHLHE40-AS1 | -0.029602826 | 0.107939 | -0.27426 | 0.783889 | NA |
| CASC14 | 0.181515018 | 0.108274 | 1.676438 | 0.093652 | NA |
| CHKB-AS1 | 0.070583182 | 0.103823 | 0.679841 | 0.496605 | NA |
| CTB-113P19.4 | 0.166333684 | 0.126408 | 1.315847 | 0.188225 | NA |
| CTB-25B13.13 | -0.058669126 | 0.107882 | -0.54383 | 0.586561 | NA |
| CTB-35F21.1 | -0.038092883 | 0.12407 | -0.30703 | 0.758823 | NA |
| CTB-5E10.3 | -0.012515714 | 0.106875 | -0.11711 | 0.906776 | NA |
| CTB-78F1.1 | 0.020045086 | 0.119868 | 0.167226 | 0.867192 | NA |
| CTC-244M17.1 | -0.11884268 | 0.102812 | -1.15592 | 0.247713 | NA |
| CTC-273B12.10 | 0.12759037 | 0.106943 | 1.193069 | 0.232842 | NA |
| CTC-303L1.2 | -0.084347292 | 0.124444 | -0.67779 | 0.497903 | NA |
| CTC-308K20.1 | -0.003633191 | 0.099519 | -0.03651 | 0.970878 | NA |
| CTC-366B18.4 | 0.00874493 | 0.111114 | 0.078703 | 0.937269 | NA |
| CTC-444N24.6 | -0.086281686 | 0.113114 | -0.76279 | 0.445591 | NA |
| CTC-451A6.4 | -0.033084375 | 0.113228 | -0.29219 | 0.77014 | NA |
| CTC-550B14.7 | 0.019077716 | 0.135244 | 0.141062 | 0.887821 | NA |
| CTD-2006C1.12 | -0.052765666 | 0.099456 | -0.53055 | 0.595734 | NA |
| CTD-2012K14.7 | -0.043417266 | 0.105117 | -0.41304 | 0.679579 | NA |
| CTD-2031P19.3 | -0.037165255 | 0.104585 | -0.35536 | 0.722319 | NA |
| CTD-2085J24.4 | 0.054620868 | 0.100339 | 0.544362 | 0.586193 | NA |
| CTD-2132N18.2 | -0.022948906 | 0.107946 | -0.2126 | 0.831641 | NA |
| CTD-2196E14.6 | -0.195873378 | 0.102265 | -1.91535 | 0.055448 | NA |
| CTD-2228A4.1 | 0.068100927 | 0.129662 | 0.525219 | 0.599431 | NA |
| CTD-2246P4.1 | 0.023012273 | 0.118622 | 0.193997 | 0.846178 | NA |
| CTD-2256P15.2 | 0.107245845 | 0.106086 | 1.010936 | 0.312047 | NA |
| CTD-2297D10.2 | 0.284608931 | 0.130283 | 2.184539 | 0.028923 | NA |
| CTD-2341M24.1 | -0.00241473 | 0.116743 | -0.02068 | 0.983498 | NA |
| CTD-2380F24.1 | -0.278383996 | 0.126932 | -2.19317 | 0.028295 | NA |
| CTD-2517O10.6 | -0.132020632 | 0.099076 | -1.33252 | 0.182688 | NA |
| CTD-2538C1.2 | 0.08847271 | 0.113401 | 0.780177 | 0.435286 | NA |
| CTD-2541M15.3 | -0.027334431 | 0.114641 | -0.23844 | 0.811544 | NA |
| CTD-2545M3.8 | -0.223477511 | 0.120126 | -1.86036 | 0.062835 | NA |
| CTD-2587M2.1 | 0.01882688 | 0.118537 | 0.158827 | 0.873806 | NA |
| CTD-2619J13.16 | 0.001984925 | 0.106633 | 0.018614 | 0.985149 | NA |
| CTD-3247F14.2 | -0.051097895 | 0.125807 | -0.40616 | 0.684624 | NA |
| DACT3-AS1 | 0.155527914 | 0.102485 | 1.517571 | 0.129123 | NA |
| DDR1-AS1 | -0.059775994 | 0.106438 | -0.56161 | 0.574385 | NA |
| DLEU7-AS1 | -0.114085526 | 0.136573 | -0.83535 | 0.403523 | NA |
| DLG5-AS1 | 0.229566319 | 0.108922 | 2.107628 | 0.035063 | NA |
| DNM1P35 | 0.08421367 | 0.102764 | 0.819483 | 0.412511 | NA |
| DNM3-IT1 | -0.281872615 | 0.115893 | -2.43217 | 0.015009 | NA |
| DOCK9-AS2 | 0.25323333 | 0.118197 | 2.142467 | 0.032156 | NA |
| DPYD-IT1 | -0.031728461 | 0.09587 | -0.33095 | 0.740679 | NA |
| DSCR9 | 0.247176854 | 0.115216 | 2.145339 | 0.031926 | NA |
| FER1L6-AS2 | 0.162289575 | 0.123207 | 1.317207 | 0.187769 | NA |
| FGF14-AS2 | -0.220677439 | 0.125399 | -1.75981 | 0.07844 | NA |
| FRY-AS1 | 0.09629745 | 0.106565 | 0.903649 | 0.366181 | NA |
| GS1-115G20.1 | -0.118088283 | 0.107234 | -1.10122 | 0.270801 | NA |
| GS1-259H13.2 | -0.028293889 | 0.114514 | -0.24708 | 0.804848 | NA |
| GS1-57L11.1 | 0.25564659 | 0.136941 | 1.866841 | 0.061924 | NA |
| HEXA-AS1 | -0.01237114 | 0.108189 | -0.11435 | 0.908963 | NA |
| HLCS-IT1 | 0.121420775 | 0.105831 | 1.14731 | 0.251253 | NA |
| HM13-IT1 | 0.178735562 | 0.103161 | 1.732585 | 0.083169 | NA |
| HPN-AS1 | -0.271702324 | 0.132253 | -2.05441 | 0.039936 | NA |
| IL21R-AS1 | 0.167155146 | 0.136115 | 1.228045 | 0.21943 | NA |
| ITGB2-AS1 | -0.256094567 | 0.135229 | -1.89378 | 0.058254 | NA |
| ITPR1-AS1 | 0.098273351 | 0.115906 | 0.847872 | 0.396509 | NA |
| KB-1125A3.11 | 0.20835006 | 0.10161 | 2.050485 | 0.040317 | NA |
| KB-1572G7.2 | -0.037824488 | 0.100558 | -0.37615 | 0.706808 | NA |
| KB-1836B5.1 | 0.092289434 | 0.102538 | 0.900051 | 0.368093 | NA |
| KB-208E9.1 | 0.340879717 | 0.124506 | 2.737851 | 0.006184 | NA |
| KLHL7-AS1 | 0.249047859 | 0.13237 | 1.881456 | 0.05991 | NA |
| KMT2E-AS1 | 0.033380388 | 0.111596 | 0.299118 | 0.76485 | NA |
| LA16c-381G6.1 | 0.224551859 | 0.132851 | 1.690258 | 0.090979 | NA |
| LIMD1-AS1 | 0.034088425 | 0.109065 | 0.312552 | 0.754621 | NA |
| LINC00237 | -0.092368252 | 0.113104 | -0.81667 | 0.414118 | NA |
| LINC00277 | 0.220231419 | 0.114675 | 1.920488 | 0.054796 | NA |
| LINC00384 | 0.115207723 | 0.117881 | 0.977322 | 0.32841 | NA |
| LINC00562 | -0.033304861 | 0.101886 | -0.32688 | 0.743756 | NA |
| LINC00568 | -0.010358114 | 0.099896 | -0.10369 | 0.917417 | NA |
| LINC00954 | -0.00705522 | 0.101534 | -0.06949 | 0.944602 | NA |
| LINC00958 | -0.281717407 | 0.111366 | -2.52966 | 0.011417 | NA |
| LINC01085 | 0.186443827 | 0.111358 | 1.674268 | 0.094078 | NA |
| LL22NC03-N27C7.1 | -0.111947938 | 0.102909 | -1.08784 | 0.276667 | NA |
| MACROD2-IT1 | 0.065391633 | 0.115821 | 0.564593 | 0.572351 | NA |
| MIR17HG | -0.228271777 | 0.12707 | -1.79643 | 0.072427 | NA |
| NEGR1-IT1 | -0.122242163 | 0.109832 | -1.11299 | 0.265711 | NA |
| NPHP3-AS1 | -0.00422594 | 0.12269 | -0.03444 | 0.972523 | NA |
| PCED1B-AS1 | -0.143991377 | 0.126479 | -1.13846 | 0.254928 | NA |
| PHEX-AS1 | 0.050508581 | 0.112081 | 0.450642 | 0.652247 | NA |
| RC3H1-IT1 | -0.15642222 | 0.097179 | -1.60962 | 0.10748 | NA |
| RN7SL832P | -0.036342453 | 0.10491 | -0.34642 | 0.72903 | NA |
| RP1-156L9.1 | 0.229124507 | 0.134677 | 1.701292 | 0.088888 | NA |
| RP1-167G20.1 | -0.026339505 | 0.137154 | -0.19204 | 0.847708 | NA |
| RP1-168P16.2 | 0.002786855 | 0.112457 | 0.024782 | 0.980229 | NA |
| RP1-1J6.2 | -0.063411319 | 0.111897 | -0.56669 | 0.570923 | NA |
| RP1-234P15.4 | -0.19777481 | 0.102918 | -1.92167 | 0.054647 | NA |
| RP1-293L6.1 | 0.026117224 | 0.110099 | 0.237215 | 0.81249 | NA |
| RP1-30E17.2 | 0.048989837 | 0.10031 | 0.488384 | 0.625278 | NA |
| RP1-310O13.7 | 0.172824566 | 0.118946 | 1.452965 | 0.146234 | NA |
| RP1-35C21.2 | -0.035392339 | 0.123453 | -0.28669 | 0.774352 | NA |
| RP1-80N2.3 | -0.165651692 | 0.10731 | -1.54367 | 0.122668 | NA |
| RP1-93H18.1 | -0.199206928 | 0.095876 | -2.07775 | 0.037733 | NA |
| RP11-1017G21.6 | 0.08414655 | 0.100198 | 0.839799 | 0.401021 | NA |
| RP11-1018N14.2 | -0.351377552 | 0.135387 | -2.59537 | 0.009449 | NA |
| RP11-1081L13.4 | 0.003169211 | 0.107111 | 0.029588 | 0.976396 | NA |
| RP11-1084I9.1 | 0.444330045 | 0.122697 | 3.621372 | 0.000293 | NA |
| RP11-109A6.3 | 0.272239454 | 0.109872 | 2.477782 | 0.01322 | NA |
| RP11-10N23.2 | -0.122944695 | 0.104799 | -1.17315 | 0.240734 | NA |
| RP11-1152H14.1 | 0.073339508 | 0.108015 | 0.678978 | 0.497152 | NA |
| RP11-117L5.4 | 0.05226127 | 0.12487 | 0.418526 | 0.675563 | NA |
| RP11-118B22.4 | 0.204056562 | 0.120763 | 1.689732 | 0.091079 | NA |
| RP11-1191J2.5 | 0.089117944 | 0.105042 | 0.848402 | 0.396214 | NA |
| RP11-124N2.1 | 0.161230142 | 0.098315 | 1.639939 | 0.101018 | NA |
| RP11-1275H24.2 | -0.206707022 | 0.103765 | -1.99206 | 0.046364 | NA |
| RP11-1277A3.1 | 0.085742755 | 0.105803 | 0.810403 | 0.417708 | NA |
| RP11-130F10.1 | 0.010572687 | 0.133027 | 0.079478 | 0.936653 | NA |
| RP11-131K5.2 | -0.114266012 | 0.122878 | -0.92992 | 0.352414 | NA |
| RP11-134K13.4 | -0.086430665 | 0.109218 | -0.79136 | 0.428733 | NA |
| RP11-135J2.4 | -0.008309378 | 0.118173 | -0.07032 | 0.943943 | NA |
| RP11-152N13.5 | -0.126203465 | 0.102858 | -1.22697 | 0.219834 | NA |
| RP11-156L14.1 | -0.2975083 | 0.133814 | -2.2233 | 0.026195 | NA |
| RP11-158I9.7 | -0.09128295 | 0.101819 | -0.89652 | 0.369976 | NA |
| RP11-158J3.2 | -0.091077097 | 0.121608 | -0.74894 | 0.453894 | NA |
| RP11-15A1.7 | -0.158760068 | 0.098159 | -1.61738 | 0.105797 | NA |
| RP11-166P13.3 | -0.02474908 | 0.112352 | -0.22028 | 0.825651 | NA |
| RP11-171A24.3 | -0.009925491 | 0.132356 | -0.07499 | 0.940222 | NA |
| RP11-173B14.4 | 0.06791612 | 0.116819 | 0.581378 | 0.560986 | NA |
| RP11-179B2.2 | -0.08272877 | 0.107996 | -0.76603 | 0.443657 | NA |
| RP11-17A1.3 | 7.72E-06 | 0.105184 | 7.34E-05 | 0.999941 | NA |
| RP11-181B11.1 | 0.138966965 | 0.102173 | 1.360117 | 0.173793 | NA |
| RP11-184A2.3 | 0.189762608 | 0.123136 | 1.541086 | 0.123296 | NA |
| RP11-193H5.1 | -0.093869953 | 0.136835 | -0.68601 | 0.492707 | NA |
| RP11-209D14.2 | 0.19220792 | 0.121419 | 1.58301 | 0.113419 | NA |
| RP11-20I23.6 | 0.066323961 | 0.120733 | 0.549343 | 0.58277 | NA |
| RP11-212D3.4 | -0.142970708 | 0.113598 | -1.25857 | 0.208185 | NA |
| RP11-215N21.1 | 0.061340357 | 0.122996 | 0.498717 | 0.617979 | NA |
| RP11-216B9.6 | -0.083406051 | 0.098979 | -0.84267 | 0.399415 | NA |
| RP11-216P16.2 | 0.017542901 | 0.108268 | 0.162032 | 0.871281 | NA |
| RP11-22P6.2 | -0.035890852 | 0.102827 | -0.34904 | 0.727059 | NA |
| RP11-231I16.1 | 0.029095498 | 0.119405 | 0.24367 | 0.807486 | NA |
| RP11-236J17.5 | 0.062845806 | 0.114397 | 0.549366 | 0.582754 | NA |
| RP11-23E19.2 | 0.042298855 | 0.104161 | 0.40609 | 0.684676 | NA |
| RP11-247A12.2 | -0.017346722 | 0.107145 | -0.1619 | 0.871385 | NA |
| RP11-248J18.2 | -0.238497296 | 0.129031 | -1.84837 | 0.064549 | NA |
| RP11-24C3.2 | 0.110364252 | 0.109976 | 1.003533 | 0.315604 | NA |
| RP11-253I19.3 | -0.002286124 | 0.110461 | -0.0207 | 0.983488 | NA |
| RP11-263C24.1 | -0.018144751 | 0.108678 | -0.16696 | 0.867403 | NA |
| RP11-264L1.3 | -0.226164194 | 0.110469 | -2.04731 | 0.040628 | NA |
| RP11-265N6.1 | -0.04914322 | 0.099909 | -0.49188 | 0.622806 | NA |
| RP11-26J3.1 | -0.068723803 | 0.120627 | -0.56972 | 0.568866 | NA |
| RP11-274B21.8 | 0.0595866 | 0.111416 | 0.534814 | 0.592778 | NA |
| RP11-284M14.1 | -0.338885561 | 0.106865 | -3.17116 | 0.001518 | NA |
| RP11-285J16.1 | -0.203029147 | 0.117563 | -1.72698 | 0.084172 | NA |
| RP11-286E11.2 | -0.239242682 | 0.116304 | -2.05704 | 0.039683 | NA |
| RP11-290F5.1 | -0.236298879 | 0.104515 | -2.2609 | 0.023765 | NA |
| RP11-295D4.1 | 0.133851901 | 0.117824 | 1.136033 | 0.255943 | NA |
| RP11-295M3.2 | -0.179946956 | 0.102631 | -1.75334 | 0.079543 | NA |
| RP11-298D21.1 | -0.150280982 | 0.129564 | -1.1599 | 0.246089 | NA |
| RP11-298E9.6 | 0.258689144 | 0.124714 | 2.074257 | 0.038055 | NA |
| RP11-299G20.2 | 0.062476269 | 0.097995 | 0.637545 | 0.52377 | NA |
| RP11-300A12.2 | 0.104710968 | 0.117456 | 0.891493 | 0.372665 | NA |
| RP11-301G7.1 | -0.100466602 | 0.115893 | -0.86689 | 0.386 | NA |
| RP11-304F15.4 | 0.101881885 | 0.130688 | 0.77958 | 0.435638 | NA |
| RP11-305F18.1 | 0.004647182 | 0.114105 | 0.040727 | 0.967513 | NA |
| RP11-30K9.4 | -0.145501135 | 0.107617 | -1.35203 | 0.176367 | NA |
| RP11-310E22.4 | 0.252473827 | 0.120658 | 2.092481 | 0.036395 | NA |
| RP11-317N8.3 | 0.079429231 | 0.097246 | 0.816788 | 0.41405 | NA |
| RP11-318C24.2 | -0.129953948 | 0.103434 | -1.2564 | 0.208971 | NA |
| RP11-320M2.1 | 0.116336359 | 0.096113 | 1.210414 | 0.22612 | NA |
| RP11-320P7.1 | 0.162147181 | 0.110868 | 1.462524 | 0.143598 | NA |
| RP11-321C24.1 | 0.053427783 | 0.105262 | 0.507569 | 0.611756 | NA |
| RP11-323I15.5 | -0.182156921 | 0.116898 | -1.55826 | 0.119172 | NA |
| RP11-325K4.3 | -0.089977748 | 0.115275 | -0.78055 | 0.435069 | NA |
| RP11-329B9.4 | -0.17459115 | 0.114705 | -1.52209 | 0.127987 | NA |
| RP11-332H18.3 | 0.132977093 | 0.109218 | 1.217541 | 0.223398 | NA |
| RP11-339B21.11 | -0.046773886 | 0.103293 | -0.45283 | 0.650674 | NA |
| RP11-341G23.4 | -0.072561168 | 0.126333 | -0.57437 | 0.56572 | NA |
| RP11-348N5.7 | -0.213119291 | 0.106057 | -2.00948 | 0.044486 | NA |
| RP11-356C4.3 | -0.033981471 | 0.110229 | -0.30828 | 0.757868 | NA |
| RP11-35G9.3 | -0.25607978 | 0.104363 | -2.45374 | 0.014138 | NA |
| RP11-363E6.3 | 0.034374337 | 0.100376 | 0.342456 | 0.732008 | NA |
| RP11-367N14.3 | -0.053151163 | 0.111417 | -0.47705 | 0.633328 | NA |
| RP11-368L12.1 | 0.085188429 | 0.125592 | 0.678296 | 0.497584 | NA |
| RP11-369E15.3 | 0.187862107 | 0.125113 | 1.501542 | 0.133216 | NA |
| RP11-378A12.1 | -0.262304153 | 0.13536 | -1.93782 | 0.052645 | NA |
| RP11-378J18.3 | -0.112671244 | 0.111715 | -1.00856 | 0.313186 | NA |
| RP11-379B18.6 | -0.037769909 | 0.12233 | -0.30875 | 0.757508 | NA |
| RP11-379F4.8 | 0.042577197 | 0.121317 | 0.35096 | 0.725619 | NA |
| RP11-381K20.5 | -0.033259345 | 0.110591 | -0.30074 | 0.763612 | NA |
| RP11-389O22.1 | -0.15100487 | 0.11393 | -1.32541 | 0.185034 | NA |
| RP11-38L15.2 | -0.108475424 | 0.108104 | -1.00343 | 0.315653 | NA |
| RP11-3B12.2 | -0.103101293 | 0.111668 | -0.92328 | 0.35586 | NA |
| RP11-400F19.8 | -0.038729594 | 0.109146 | -0.35484 | 0.722709 | NA |
| RP11-403H13.1 | -0.097216617 | 0.111071 | -0.87526 | 0.381431 | NA |
| RP11-407B7.1 | 0.088706037 | 0.102066 | 0.869103 | 0.384791 | NA |
| RP11-415J8.7 | 0.060342796 | 0.104328 | 0.578395 | 0.562998 | NA |
| RP11-417J8.6 | -0.067247058 | 0.134125 | -0.50138 | 0.616106 | NA |
| RP11-417O11.5 | 0.071927723 | 0.10611 | 0.677859 | 0.497861 | NA |
| RP11-420G6.4 | -0.212113584 | 0.127885 | -1.65862 | 0.097191 | NA |
| RP11-420L9.5 | -0.097720978 | 0.117571 | -0.83117 | 0.405879 | NA |
| RP11-422P24.11 | 0.030117729 | 0.094572 | 0.318462 | 0.750135 | NA |
| RP11-425A6.5 | 0.028472781 | 0.100138 | 0.284334 | 0.776154 | NA |
| RP11-429E11.2 | 0.107334467 | 0.133045 | 0.806754 | 0.419808 | NA |
| RP11-430H10.1 | 0.126256403 | 0.112379 | 1.123486 | 0.261231 | NA |
| RP11-438L19.1 | 0.048782279 | 0.110685 | 0.440733 | 0.659407 | NA |
| RP11-439M11.1 | 0.041890392 | 0.114985 | 0.364312 | 0.715625 | NA |
| RP11-440D17.4 | -0.136507104 | 0.106577 | -1.28083 | 0.200252 | NA |
| RP11-449P15.1 | 0.195057568 | 0.112411 | 1.735214 | 0.082703 | NA |
| RP11-44N22.3 | 0.175540485 | 0.107775 | 1.628765 | 0.103363 | NA |
| RP11-461L13.3 | -0.204108216 | 0.121306 | -1.68259 | 0.092454 | NA |
| RP11-467H10.2 | -0.112387212 | 0.100368 | -1.11975 | 0.262821 | NA |
| RP11-474G23.3 | -0.021541997 | 0.101192 | -0.21288 | 0.831418 | NA |
| RP11-481G8.2 | 0.21706572 | 0.115949 | 1.872087 | 0.061195 | NA |
| RP11-483I13.5 | 0.143179325 | 0.116165 | 1.232556 | 0.217742 | NA |
| RP11-484D2.4 | -0.064791425 | 0.099939 | -0.64831 | 0.516783 | NA |
| RP11-484P15.1 | 0.003870782 | 0.11821 | 0.032745 | 0.973878 | NA |
| RP11-486O12.2 | -0.036745302 | 0.104506 | -0.35161 | 0.72513 | NA |
| RP11-486O13.4 | 0.160863169 | 0.111822 | 1.438564 | 0.150274 | NA |
| RP11-488L18.8 | -0.055839106 | 0.108642 | -0.51397 | 0.60727 | NA |
| RP11-496D24.2 | -0.397068448 | 0.136715 | -2.90434 | 0.00368 | NA |
| RP11-499E18.1 | -0.167856389 | 0.097855 | -1.71535 | 0.086281 | NA |
| RP11-49O14.2 | -0.216590756 | 0.111085 | -1.94977 | 0.051204 | NA |
| RP11-506M12.1 | 0.091967107 | 0.109679 | 0.838515 | 0.401741 | NA |
| RP11-507K2.3 | -0.005366217 | 0.105089 | -0.05106 | 0.959275 | NA |
| RP11-51J9.5 | 0.094258443 | 0.108127 | 0.871742 | 0.383349 | NA |
| RP11-529K1.2 | -0.370440681 | 0.117084 | -3.16389 | 0.001557 | NA |
| RP11-547D23.1 | 0.04202189 | 0.104008 | 0.404026 | 0.686193 | NA |
| RP11-547D24.1 | -0.012835858 | 0.131464 | -0.09764 | 0.92222 | NA |
| RP11-552M14.1 | -0.055857411 | 0.128306 | -0.43535 | 0.663311 | NA |
| RP11-554J4.1 | 0.024715777 | 0.105934 | 0.233314 | 0.815518 | NA |
| RP11-568N6.1 | 0.056882538 | 0.113418 | 0.501531 | 0.615998 | NA |
| RP11-572M11.4 | -0.022297295 | 0.103001 | -0.21648 | 0.828616 | NA |
| RP11-574O16.1 | 0.202060368 | 0.127913 | 1.579675 | 0.114181 | NA |
| RP11-579E24.1 | 0.016637759 | 0.119998 | 0.13865 | 0.889727 | NA |
| RP11-57A19.5 | 0.175240446 | 0.137178 | 1.27747 | 0.201436 | NA |
| RP11-580I16.2 | -0.1207632 | 0.111659 | -1.08154 | 0.279459 | NA |
| RP11-589M4.1 | -0.299030283 | 0.11762 | -2.54235 | 0.011011 | NA |
| RP11-589P10.5 | -0.073071805 | 0.109071 | -0.66994 | 0.502893 | NA |
| RP11-5C23.1 | -0.251376536 | 0.125924 | -1.99626 | 0.045906 | NA |
| RP11-5N11.2 | -0.055166845 | 0.116107 | -0.47514 | 0.634688 | NA |
| RP11-605F22.2 | 0.093017487 | 0.110234 | 0.84382 | 0.39877 | NA |
| RP11-60A8.1 | 0.005510203 | 0.134144 | 0.041077 | 0.967235 | NA |
| RP11-613F22.8 | -0.017883435 | 0.101665 | -0.17591 | 0.860368 | NA |
| RP11-638I2.2 | -0.183491691 | 0.098377 | -1.86518 | 0.062156 | NA |
| RP11-667K14.3 | -0.014938282 | 0.135333 | -0.11038 | 0.912107 | NA |
| RP11-674N23.4 | 0.149711156 | 0.119754 | 1.250156 | 0.211243 | NA |
| RP11-675F6.3 | 0.183721869 | 0.115748 | 1.587261 | 0.112453 | NA |
| RP11-677O4.6 | 0.118781621 | 0.122503 | 0.969623 | 0.332234 | NA |
| RP11-68I3.10 | 0.162528782 | 0.107278 | 1.51503 | 0.129765 | NA |
| RP11-68I3.11 | -0.129673321 | 0.102505 | -1.26505 | 0.205854 | NA |
| RP11-6N17.6 | -0.207259697 | 0.114983 | -1.80252 | 0.071464 | NA |
| RP11-706O15.5 | 0.00277583 | 0.07798 | 0.035597 | 0.971604 | NA |
| RP11-70J12.1 | -0.260553337 | 0.126472 | -2.06017 | 0.039383 | NA |
| RP11-714G18.1 | -0.063745176 | 0.111849 | -0.56992 | 0.568731 | NA |
| RP11-715J22.4 | 0.1221821 | 0.109594 | 1.114863 | 0.264909 | NA |
| RP11-71E19.1 | 0.196437437 | 0.11201 | 1.753755 | 0.079472 | NA |
| RP11-71H17.9 | -0.12211909 | 0.106121 | -1.15075 | 0.249836 | NA |
| RP11-728E14.3 | 0.005285582 | 0.11762 | 0.044938 | 0.964157 | NA |
| RP11-72M17.1 | -0.005849823 | 0.101551 | -0.0576 | 0.954063 | NA |
| RP11-731C17.2 | -0.198001634 | 0.11216 | -1.76535 | 0.077506 | NA |
| RP11-74E22.3 | -0.060450676 | 0.101229 | -0.59717 | 0.550397 | NA |
| RP11-761I4.3 | 0.174469638 | 0.108256 | 1.611643 | 0.10704 | NA |
| RP11-768F21.1 | 0.169910481 | 0.111027 | 1.530352 | 0.12593 | NA |
| RP11-769N22.1 | -0.104776465 | 0.099367 | -1.05443 | 0.291684 | NA |
| RP11-76I14.1 | -0.208954751 | 0.108459 | -1.92658 | 0.054032 | NA |
| RP11-77K12.3 | -0.106750759 | 0.099534 | -1.0725 | 0.283495 | NA |
| RP11-799D4.4 | 0.029407223 | 0.105223 | 0.279476 | 0.779879 | NA |
| RP11-799M12.2 | 0.014859161 | 0.118205 | 0.125707 | 0.899964 | NA |
| RP11-802O23.3 | -0.294722304 | 0.128105 | -2.30063 | 0.021412 | NA |
| RP11-809O17.1 | -0.027957851 | 0.11345 | -0.24643 | 0.805346 | NA |
| RP11-81H3.2 | -0.257298161 | 0.136909 | -1.87933 | 0.060199 | NA |
| RP11-849F2.8 | -0.087741734 | 0.105584 | -0.83101 | 0.405966 | NA |
| RP11-84G21.1 | -0.147684771 | 0.097986 | -1.50721 | 0.131757 | NA |
| RP11-85F14.5 | -0.245306111 | 0.117276 | -2.09169 | 0.036466 | NA |
| RP11-863P13.5 | 0.183053225 | 0.118416 | 1.545843 | 0.122142 | NA |
| RP11-867G2.2 | 0.184351525 | 0.114225 | 1.613934 | 0.106542 | NA |
| RP11-86H7.7 | -0.000504285 | 0.116938 | -0.00431 | 0.996559 | NA |
| RP11-87H9.3 | -0.313852043 | 0.131816 | -2.38098 | 0.017267 | NA |
| RP11-923I11.4 | 0.199292942 | 0.110457 | 1.804254 | 0.071192 | NA |
| RP11-93L9.1 | -0.269863137 | 0.12148 | -2.22145 | 0.02632 | NA |
| RP11-95P2.1 | -0.097179325 | 0.100844 | -0.96366 | 0.335218 | NA |
| RP11-96D1.3 | 0.191547878 | 0.099673 | 1.921764 | 0.054635 | NA |
| RP11-982M15.8 | -0.175875881 | 0.115561 | -1.52194 | 0.128025 | NA |
| RP11-983P16.2 | -0.030395198 | 0.10427 | -0.2915 | 0.770665 | NA |
| RP11-9L18.3 | 0.188649924 | 0.109257 | 1.726656 | 0.084229 | NA |
| RP13-270P17.3 | -0.185236823 | 0.115817 | -1.5994 | 0.109732 | NA |
| RP3-323P24.3 | 0.11259717 | 0.097375 | 1.156326 | 0.247548 | NA |
| RP3-326L13.3 | -0.308118055 | 0.126031 | -2.44477 | 0.014494 | NA |
| RP3-388M5.9 | -0.056777335 | 0.10177 | -0.5579 | 0.576915 | NA |
| RP3-466P17.1 | -0.112587883 | 0.107523 | -1.04711 | 0.295049 | NA |
| RP3-467L1.6 | -0.245778133 | 0.106022 | -2.31818 | 0.020439 | NA |
| RP3-522D1.1 | 0.087300952 | 0.095558 | 0.913591 | 0.360932 | NA |
| RP4-593C16.3 | -0.056150909 | 0.10966 | -0.51204 | 0.60862 | NA |
| RP4-633I8.4 | -0.171902564 | 0.105221 | -1.63373 | 0.102315 | NA |
| RP4-734G22.3 | 0.296363404 | 0.12349 | 2.399897 | 0.0164 | NA |
| RP4-794H19.2 | -0.088692043 | 0.104868 | -0.84575 | 0.397693 | NA |
| RP5-1092A3.4 | 0.020540732 | 0.115185 | 0.178329 | 0.858465 | NA |
| RP5-1198O20.4 | -0.152589191 | 0.11255 | -1.35574 | 0.175181 | NA |
| RP5-837J1.4 | -0.205005943 | 0.129776 | -1.57969 | 0.114179 | NA |
| RP5-867C24.4 | 0.031137376 | 0.110764 | 0.281115 | 0.778622 | NA |
| RP5-884C9.2 | 0.154508331 | 0.10535 | 1.466622 | 0.142479 | NA |
| RP5-903G2.2 | 0.052845811 | 0.113586 | 0.465249 | 0.641753 | NA |
| RP6-74O6.6 | -0.006886957 | 0.114204 | -0.0603 | 0.951913 | NA |
| SLIT2-IT1 | -0.003496897 | 0.113068 | -0.03093 | 0.975327 | NA |
| SMYD3-IT1 | 0.040252501 | 0.112601 | 0.35748 | 0.720732 | NA |
| SNORA40 | -0.0011863 | 0.104044 | -0.0114 | 0.990903 | NA |
| SNORA59B | -0.031153004 | 0.10906 | -0.28565 | 0.775146 | NA |
| SPTY2D1-AS1 | -0.238658567 | 0.104393 | -2.28615 | 0.022245 | NA |
| ST6GAL2-IT1 | 0.063076461 | 0.116008 | 0.543727 | 0.58663 | NA |
| TAPSAR1 | -0.245098663 | 0.12155 | -2.01644 | 0.043754 | NA |
| TEX41 | 0.009241134 | 0.111501 | 0.08288 | 0.933947 | NA |
| TNR-IT1 | 0.07479497 | 0.113357 | 0.659815 | 0.509372 | NA |
| TSSC1-IT1 | 0.197918497 | 0.103349 | 1.915055 | 0.055485 | NA |
| U95743.1 | 0.188866582 | 0.118269 | 1.596918 | 0.110284 | NA |
| XXbac-B476C20.14 | -0.003421119 | 0.110536 | -0.03095 | 0.975309 | NA |
| XXcos-LUCA11.4 | 0.008383416 | 0.101547 | 0.082557 | 0.934204 | NA |
| XXyac-YX65C7_A.2 | 0.020813111 | 0.11531 | 0.180497 | 0.856763 | NA |
| YEATS2-AS1 | -0.211009408 | 0.135861 | -1.55313 | 0.120392 | NA |
| ZNF503-AS2 | 0.22548672 | 0.100657 | 2.240153 | 0.025081 | NA |
