# Supplementary material for: Global long non-coding RNA expression in the rostral anterior cingulate cortex of depressed suicides
Source: Transl Psychiatry. 2018 Oct 18;8:224. doi: 10.1038/s41398-018-0267-7 (PMC6193959; doi:10.1038/s41398-018-0267-7)

**Supplementary Figure 5 – A)** Top 10 Molecular Functions GO terms, **B)** top 10 Biological Processes GO terms, and **C)** top 10 Cellular Components GO terms enriched in the Brown module.

**Supplementary Figure 6 – A)** Top 10 Molecular Functions GO terms, **B)** top 10 Biological Processes GO terms, and **C)** top 10 Cellular Components GO terms enriched in the Blue module.

## Supplementary Figure 5 -

**A)**

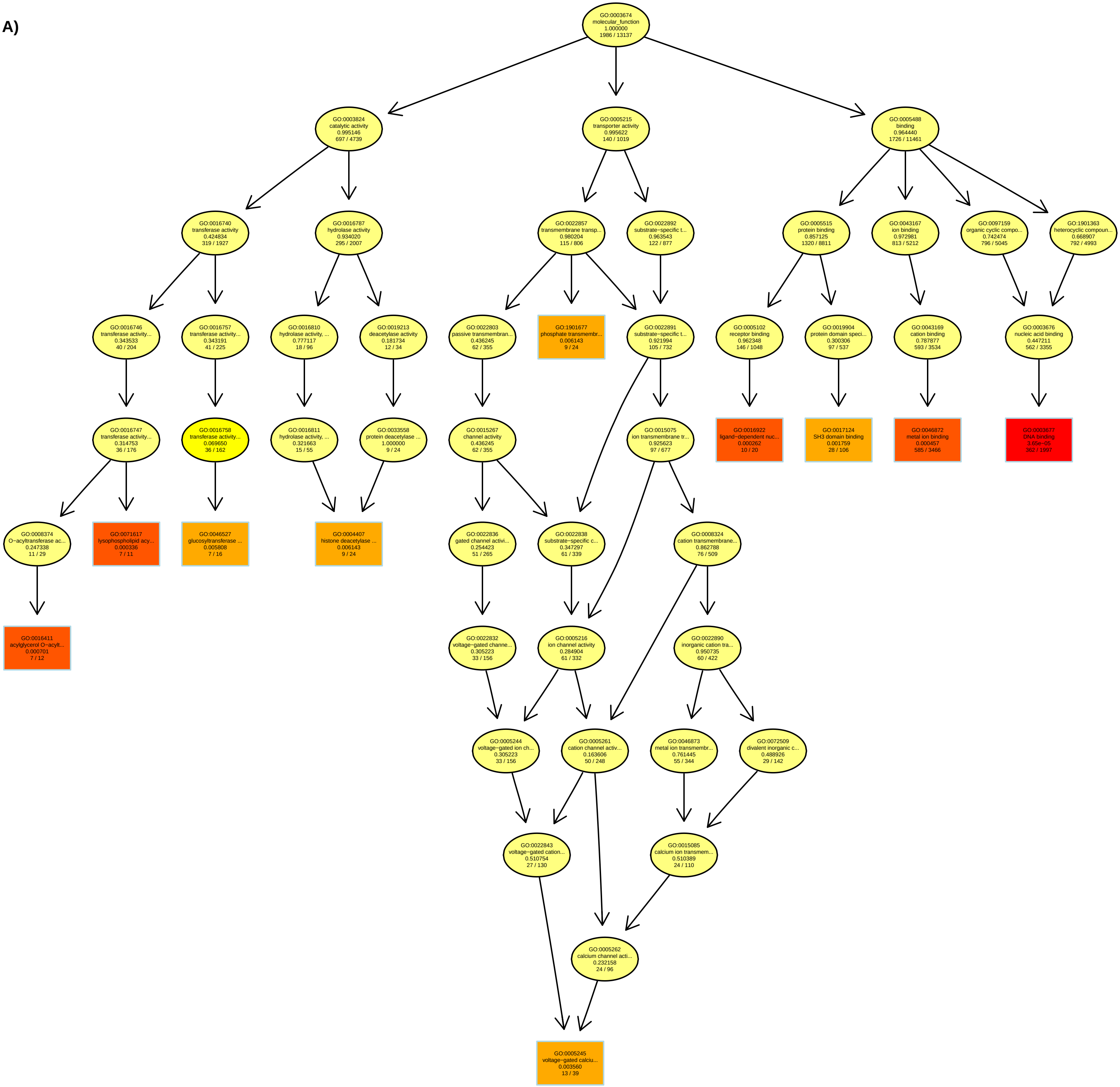

**Supplementary Figure 5 -**

**B)**

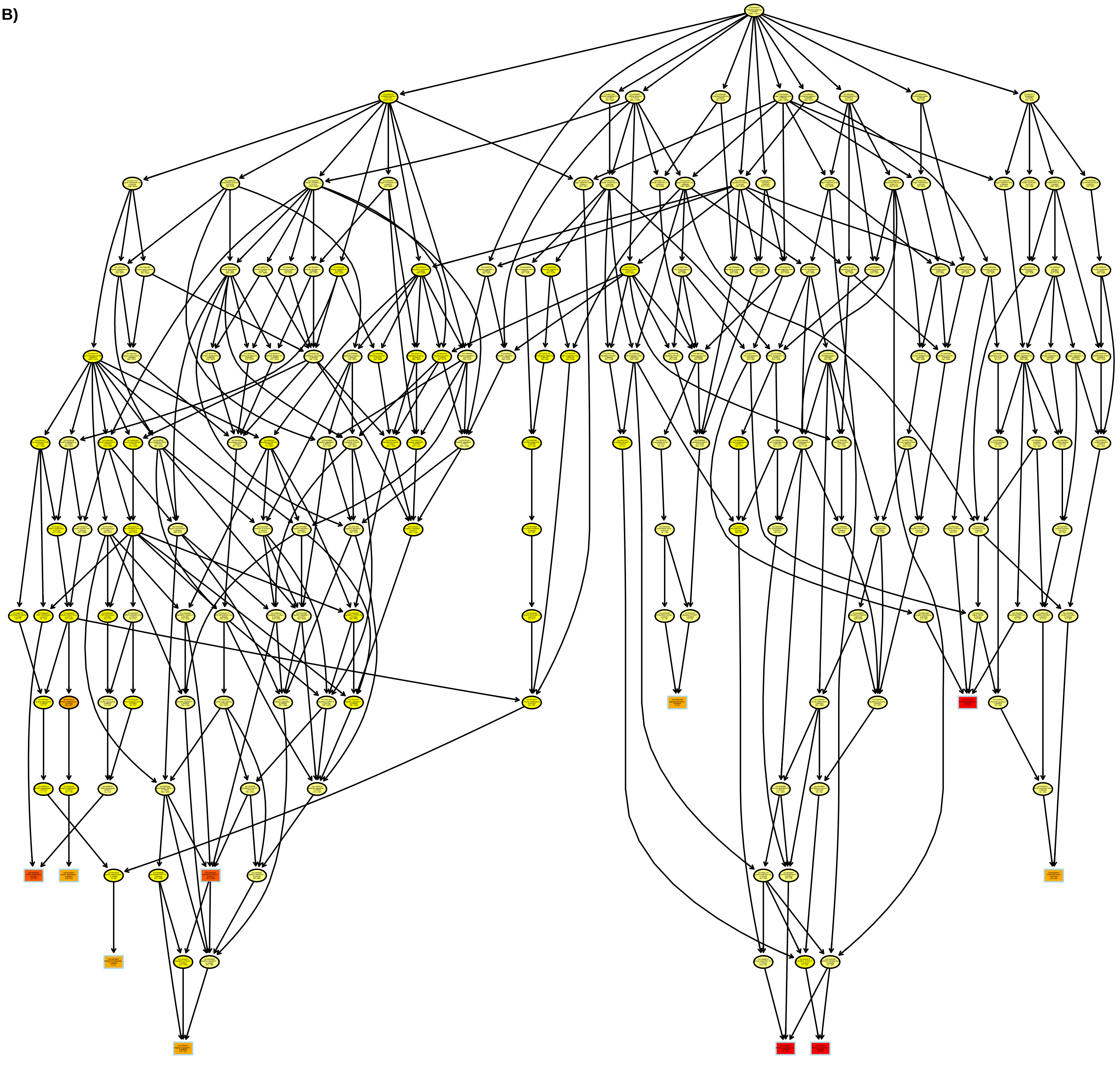

**Supplementary 5 -**

**C)**

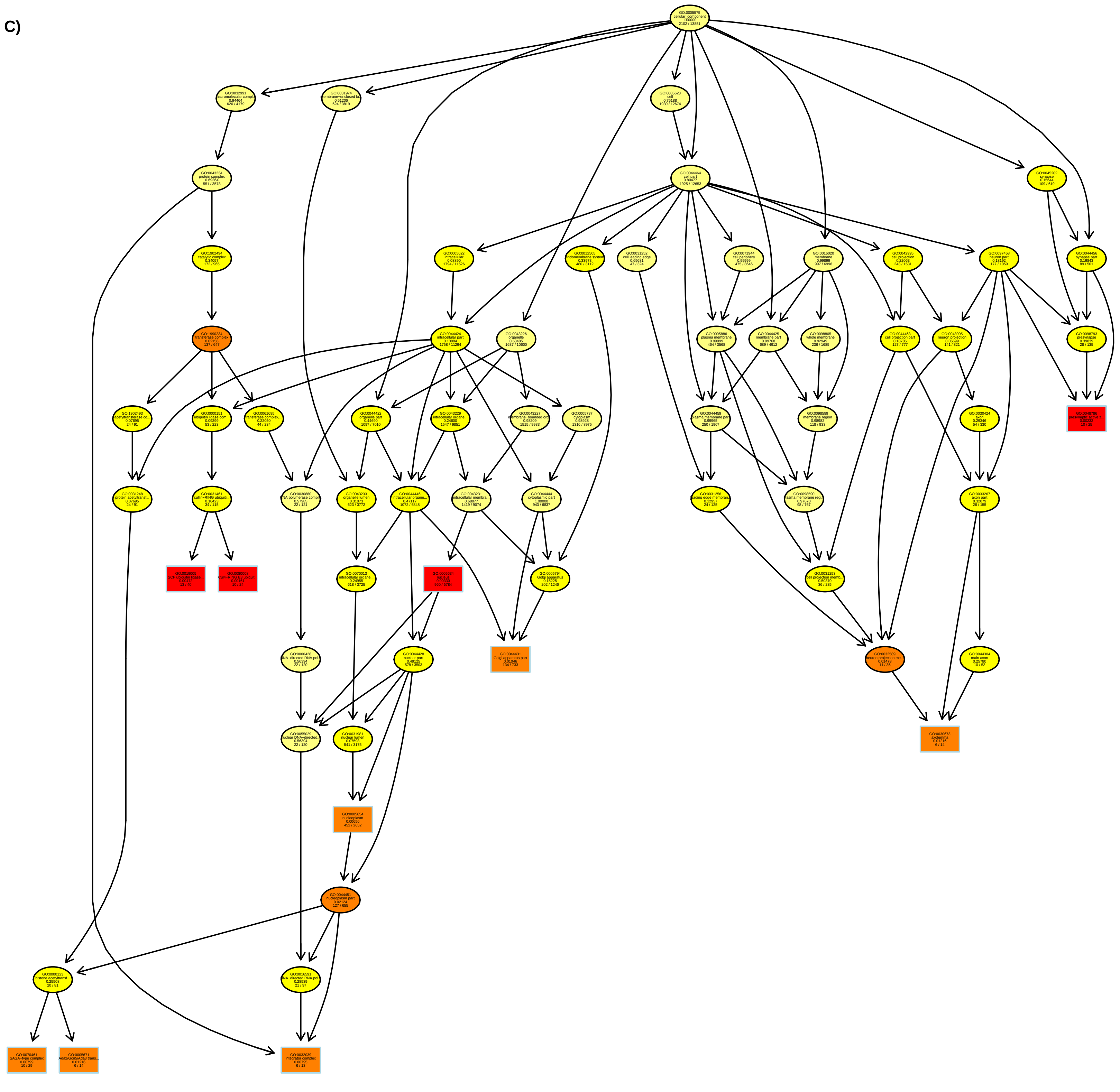

**Supplementary Figure 6 -**

**A)**

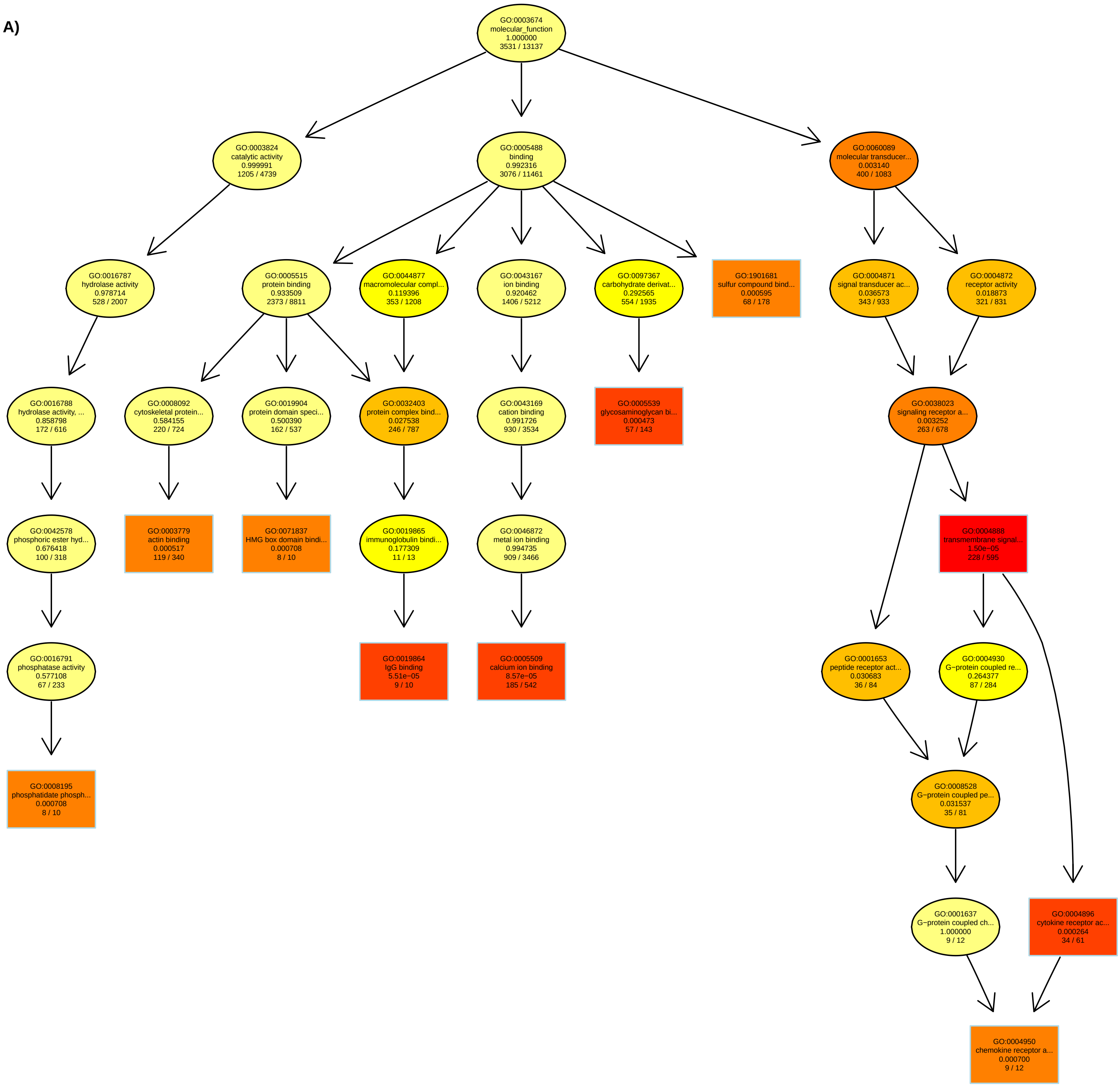

**Supplementary Figure 6 -**

**B)**

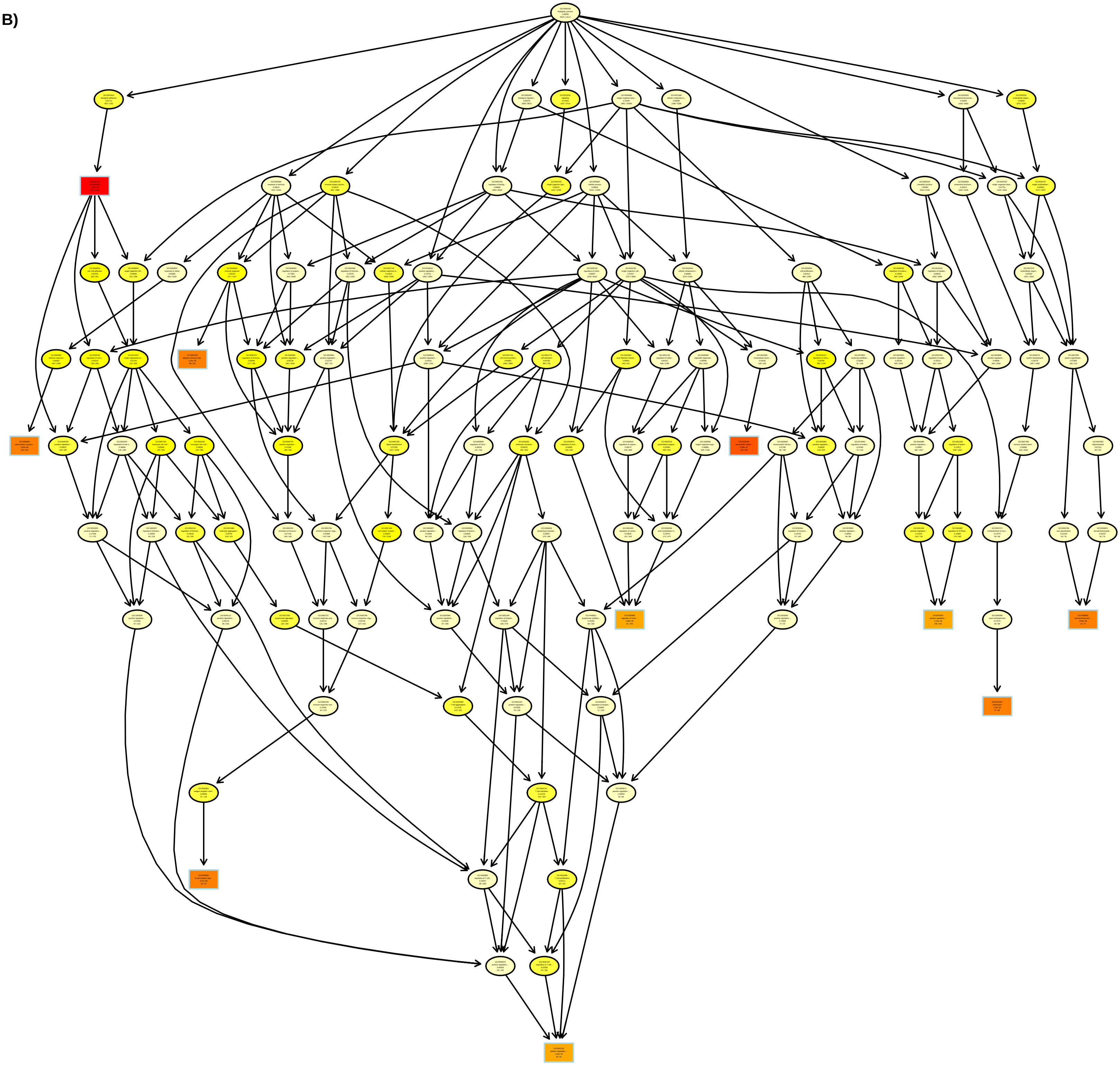

## Supplementary Figure 6 -

## C)

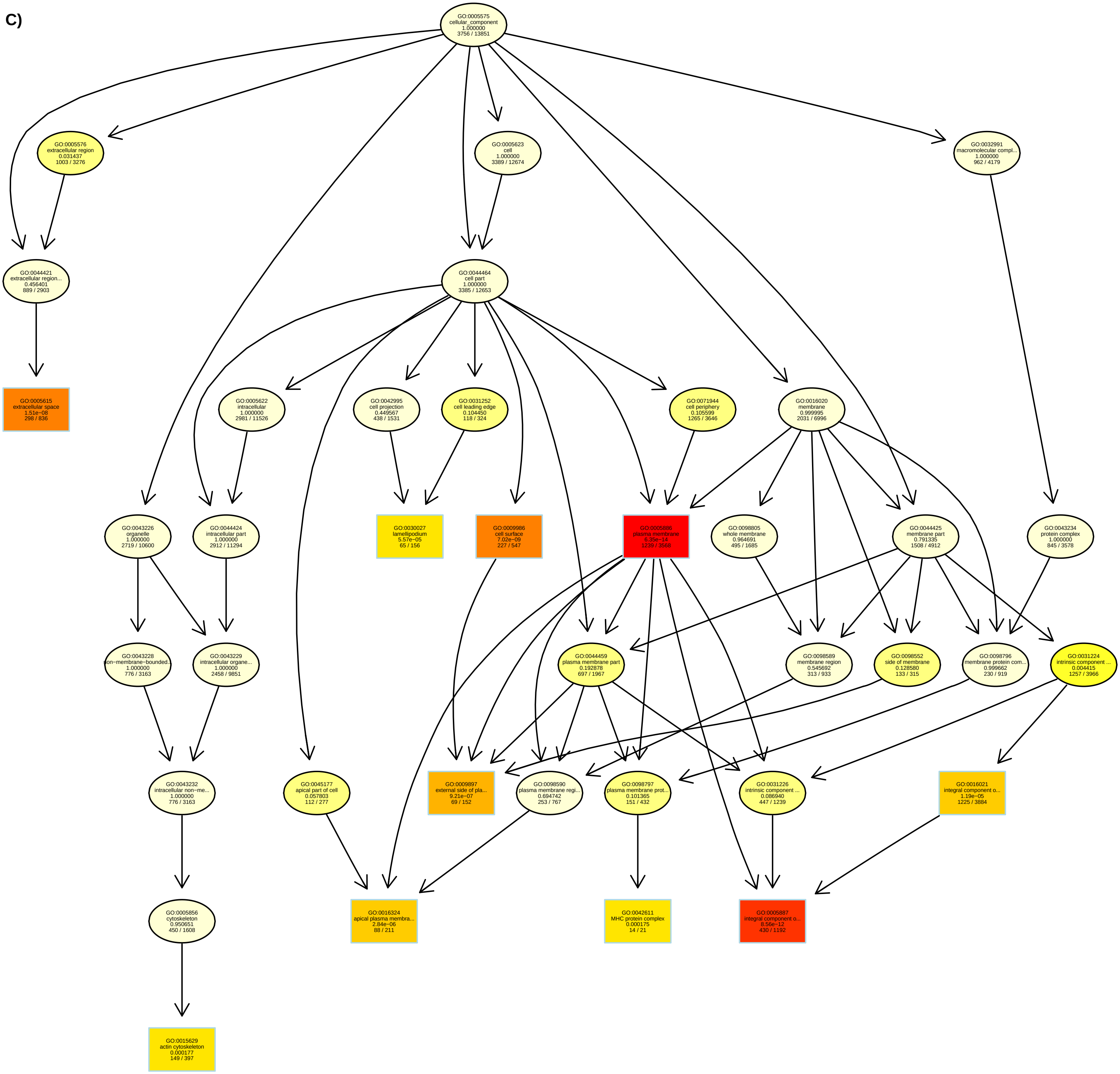

Supplement: Supplementary file 2 — Supplementary Materials Cont'd [file 41398_2018_267_MOESM2_ESM.pdf]
